# The choanoflagellate pore-forming lectin SaroL-1 punches holes in cancer cells by targeting the tumor-related glycosphingolipid Gb3

**DOI:** 10.1038/s42003-022-03869-w

**Published:** 2022-09-12

**Authors:** Simona Notova, François Bonnardel, Francesca Rosato, Lina Siukstaite, Jessica Schwaiger, Jia Hui Lim, Nicolai Bovin, Annabelle Varrot, Yu Ogawa, Winfried Römer, Frédérique Lisacek, Anne Imberty

**Affiliations:** 1 Univ. Grenoble Alpes, CNRS, CERMAV, 38000 Grenoble, France; 2 Swiss Institute of Bioinformatics, CH-1227 Geneva, Switzerland; 3 Computer Science Department, UniGe, CH-1227 Geneva, Switzerland; 4 Faculty of Biology, University of Freiburg, 79104 Freiburg, Germany; 5 Signalling Research Centers BIOSS and CIBSS, University of Freiburg, 79104 Freiburg, Germany; 6 Shemyakin-Ovchinnikov Institute of Bioorganic Chemistry, Russian Academy of Science, Moscow, 117997 Russian Federation; 7 Freiburg Institute for Advanced Studies (FRIAS), University of Freiburg, 79104 Freiburg, Germany; 8 Section of Biology, UniGe, CH-1205 Geneva, Switzerland

**Keywords:** Glycobiology, X-ray crystallography

## Abstract

Choanoflagellates are primitive protozoa used as models for animal evolution. They express a large variety of multi-domain proteins contributing to adhesion and cell communication, thereby providing a rich repertoire of molecules for biotechnology. Adhesion often involves proteins adopting a β-trefoil fold with carbohydrate-binding properties therefore classified as lectins. Sequence database screening with a dedicated method resulted in TrefLec, a database of 44714 β-trefoil candidate lectins across 4497 species. TrefLec was searched for original domain combinations, which led to single out SaroL-1 in the choanoflagellate *Salpingoeca rosetta*, that contains both β-trefoil and aerolysin-like pore-forming domains. Recombinant SaroL-1 is shown to bind galactose and derivatives, with a stronger affinity for cancer-related α-galactosylated epitopes such as the glycosphingolipid Gb3, when embedded in giant unilamellar vesicles or cell membranes. Crystal structures of complexes with Gb3 trisaccharide and GalNAc provided the basis for building a model of the oligomeric pore. Finally, recognition of the αGal epitope on glycolipids required for hemolysis of rabbit erythrocytes suggests that toxicity on cancer cells is achieved through carbohydrate-dependent pore-formation.

## Abstract (web summary)

A curated lectin database, structural characterization, and in vitro assays show that choanoflagellate lectin SaroL-1 binds to cancer-related α-galactosylated epitopes and can be toxic to cancer cells through a carbohydrate-dependent pore-formation mechanism.

## Introduction

Lectins are protein receptors that bind complex carbohydrates without modifying them, and therefore participating in the signaling function of the glycocode encoded in glycoconjugates such as glycolipids and glycoproteins at the cell surface^1,2^. Lectins participate in multiple biological processes, such as embryonic development, cell growth and immunomodulation, and are crucial for the interactions between microorganisms and host cells (pathogenicity, symbiosis). Lectin domains are often associated with other functional proteins such as enzymes or toxins. Life-threatening examples are ricin^3^ or cholera toxin^4^, in which the lectin domain is responsible for the specificity and adhesion to cell surface glycans, prior to the cellular uptake of the toxin that interferes with metabolism.

A different mode of action is observed in pore-forming toxins (PFTs) that oligomerise and create holes into membranes of bacteria or host cells^5,6^. The specific β-pore forming toxins (β-PFTs) form pores fully lined by β-strands and include the aerolysin family^7,8^. These proteins contain a conserved aerolysin *C*-terminal domain and *N*-terminal domain that adopts different topologies targeting the cell surface, some of them with a lectin-like fold. Aerolysin from *Aeromonas hydrophila* binds to glycoconjugates through a Pertussis toxin domain^9^, while cytolysin from *Vibrio cholerae* presents both β-prism and β-trefoil lectin domains^10^. In eukaryotes, lectin-dependent β-PFTs have been described in fishes, i.e., natterin-like protein from zebrafish^11^ and lamprey^12^, in sea cucumber^13^ and in fungi^14^. Such modular proteins are of high interest since the lectin specificity can be employed to induce the cytotoxicity, for example, in cancer cells, as tested with the lamprey lectin^12^. Among other strategies, identifying novel pore-forming lectins can be a valuable tool for research and therapy.

A new software has been recently developed for the identification and annotation of lectins in proteomes, thereby allowing the search of β-PFT-containing lectins. This tool takes advantage of a structure-based classification of lectins proposed in UniLectin3D, a database of manually curated and classified lectin 3D structures including their oligomeric status and carbohydrate-binding sites^15^. Tandem repeats are common in lectins, such as β-trefoils or β-propellers, and notoriously challenge conventional sequence motif finding methods. Nonetheless, detection is improved by considering each repeat independently with precise delineation based on the 3D shape. This approach was validated with β-propellers^16^ and is extended here to β-trefoils.

**Fig. 1:**
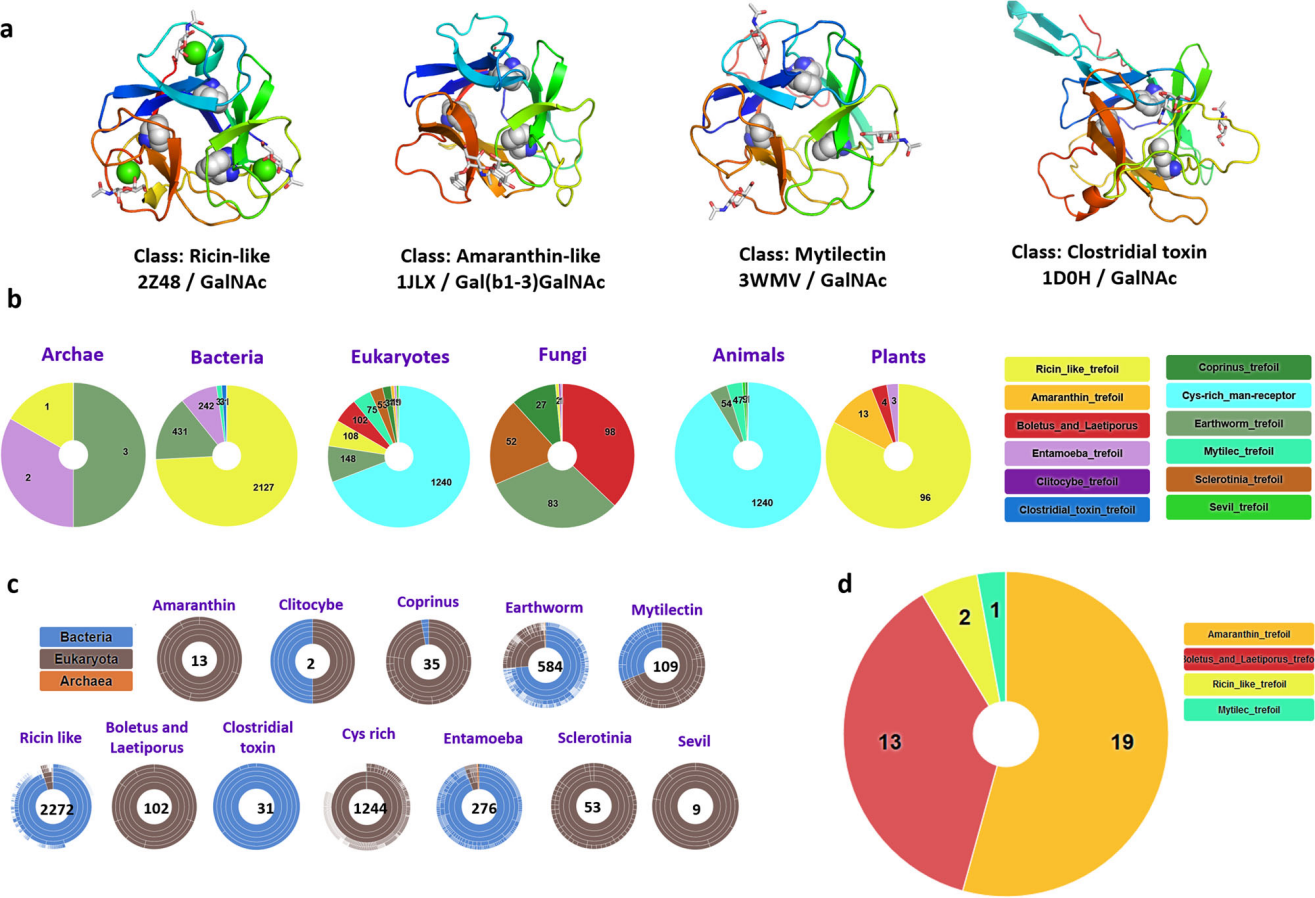
Classification of β-trefoil lectins according to the TrefLec database. **a** Selected examples of β-trefoil lectins from different classes. The binding peptide is represented by a rainbow-colored ribbon, the bound sugar by sticks, and the conserved hydrophobic core-forming amino acids by spheres. **b** Sunburst statistics for predicted β-trefoil lectins in different classes, in selected domains of life. **c** Classification of predicted β-trefoil lectins and distribution of sequences in the TrefLec database. **d** Prediction of β-trefoil lectins with an aerolysin domain based on the corresponding CATH domain (CATH entry 2.170.15.10).

β-trefoil lectins are small proteins consisting of three repeats of the same conserved binding motif, and this fold is widely distributed in nature^17^. They have been identified in bacteria, fungi, plants and animals, with a broad variety of sequences and local conformations but with conserved aromatic amino acids that create a common central hydrophobic core (Fig. 1a). β-trefoil lectins are popular in protein engineering for designing new scaffolds with high symmetry that can be associated with other domains. This functional and modular versatility is resourceful in synthetic biology, as recently reviewed^18^. Also, β-trefoil lectins bind efficiently to glycosylated surfaces and target specific cell types. For example, the Mytilec family initially identified in mussels binds to globotriaosyl ceramide (Gb3) that is overexpressed in some metastatic cancer^19–23^. Engineered Mytilec with perfectly symmetrical β-trefoil such as Mitsuba (three-leaf in Japanese) successfully demonstrates the potential of these small and symmetrical modules for recognizing cancer cells^24^. In this article, we introduce a new database of the UniLectin3D platform. TrefLec detects and classifies β-trefoil lectins resulting from screening translated genomes. TrefLec was then used to search for the occurrence of β-trefoils with an associated aerolysin domain to discover new β-PFT-lectins. We identified a candidate of interest in *Salpingoeca rosetta*, a single-cell and colony-forming micro-eukaryotic marine organism belonging to the choanoflagellates^25^, a group with unusual high diversity of multi-domain proteins involved in cell adhesion and communication^26^. We present here the structural characterization of a pore-forming protein together with its unique functionality that depends on adhesion on glycan epitope related to cancer cells.

## Results

### The TrefLec database for the prediction of β-trefoil lectins in genomes

The UniLectin3D classification spans 109 classes defined upon a 20% amino acid sequence identity cut-off. UniLectin3D contains 212 X-ray structures of β-trefoil lectins (i.e., 9% of the database content) corresponding to 63 distinct proteins. Although all structures share the same fold where hydrophobic amino acids form the β-trefoil core, they are spread across 12 different classes. The Ricin-like class is the most populated, with 123 crystal structures presenting a very conserved fold in all kingdoms of life. Other types of β-trefoil are observed in invertebrates and fungi or can be involved in botulism and tetanus infection.

A new database, named TrefLec database (https://unilectin.eu/trefoil/) was built for prediction of β-trefoil lectins. The 12 classes of β-trefoil lectins defined in UniLectin3D were used as the basis for its construction. The classes served for the identification of conserved motifs. Sequence alignment was performed at the “lobe” level for each class, i.e., using the three repeats for each sequence. Twelve characteristic Hidden Markov Model (HMM) profiles that capture amino acid variations were built, one for each class (Supplementary Fig. 1). The QxW signature that was observed earlier in Ricin-like lectins^27^ is common to most classes, although with some degeneration, confirming an evolutionary link in most β-trefoils. In some classes, such as Amaranthin or Mytilec, the classic QxW signature is absent, but the same topology is preserved for the hydrophobic core.

The 12 motifs were used to search UniProt-trEMBL, UniProt-SwissProt and NCBI-nr protein databases for all kingdoms, including bacteria, viruses, archaea and eukarya with animals, fungi, and plants, comprising 197.232.239 proteins in 108.257 species for the RefSeq release of May 2021. This resulted in the identification of 4830 filtered sequences of putative lectins using a score cut-off of 0.25 (44714 unfiltered) in 1660 species (4497 unfiltered) (Figs. 1b, c). Sequence similarity of lectins between different classes created some overlap, i.e. a small number of sequences were predicted to occur in several classes, but the proportion remained low and was calculated, for example, to be <2% across the Ricin-like, Entamoeba and earthworm classes (Supplementary Fig. 2).

The majority of sequences belong to the Ricin-like class (48%), the Cys-rich man receptor (27%) and the Earthworm-lectin (12%). All classes are not represented equally in the different kingdoms. The large Ricin-like class is over-represented in plants and bacteria. Fungi span the most extensive diversity of classes. Some classes are specific to one kingdom with for example Amaranthin-type lectins predicted to occur only in eukaryotes, specifically in plants^28^. Similarly, Coprinus β-trefoils are primarily identified in genomes of basidiomycetes fungi. Cys-rich lectins are sub-domains of membrane receptors occurring in vertebrates^29^. Clostridial toxins are only predicted in clostridial bacteria. In contrast, Mytilecs, known for their binding to cancer cells and cytolytic activity^22,30^ have been structurally characterized in mollusks and now predicted to occur in several invertebrates and bacterial species.

### Search for new β-PFT-lectins in the TrefLec database

The TrefLec database (https://unilectin.eu/trefoil/) provides information on additional domains associated with the β-trefoil domain and predicted various enzymatic or toxic functions (Supplementary Table 1). The β-trefoils of the Ricin-like class are associated with glycosyl hydrolases, lipases or other enzymes. The β-trefoil Cys-rich domain is part of the macrophage mannose receptor that also contains fibronectin and multiple C-type protein domains^29^. The web interface can be used to search for specific domains defined in reference data sources such as CATH, the Protein Structure Classification database^31^. When searching for aerolysin or proaerolysin (CATH domain 2.170.15.10), 35 lectin-containing sequences were identified (Fig. 1d). Twelve sequences from fungi contain a “Boletus_and_Laetiporus_trefoil”, all with strong similarity with the well described β-PFT-lectin LSL from *Laetiporus sulphureus*^14^. A related sequence is observed in the genome of the primitive plant *Marchantia polymorpha*. Only one β-PFT-lectin related to Ricin-type trefoil is predicted in bacteria *(Minicystis rosea*). Nineteen sequences from plants are predicted to contain an Amaranthin-type β-trefoil. Such plant toxins have not been structurally characterized, but their phylogeny has been recently reviewed and a role in the stress response was proposed^28^. In the remaining four sequences, a eukaryotic Mytilec domain was identified in the genome of *Salpingoeca rosetta*, a single-cell and colony-forming micro-eukaryotic marine organism belonging to the choanoflagellates group^25^. Lectins of the Mytilec-like class have been demonstrated to bind the αGal1-4Gal epitope on glycosphingolipid Gb3 from cancer cells^23^.

**Fig. 2:**
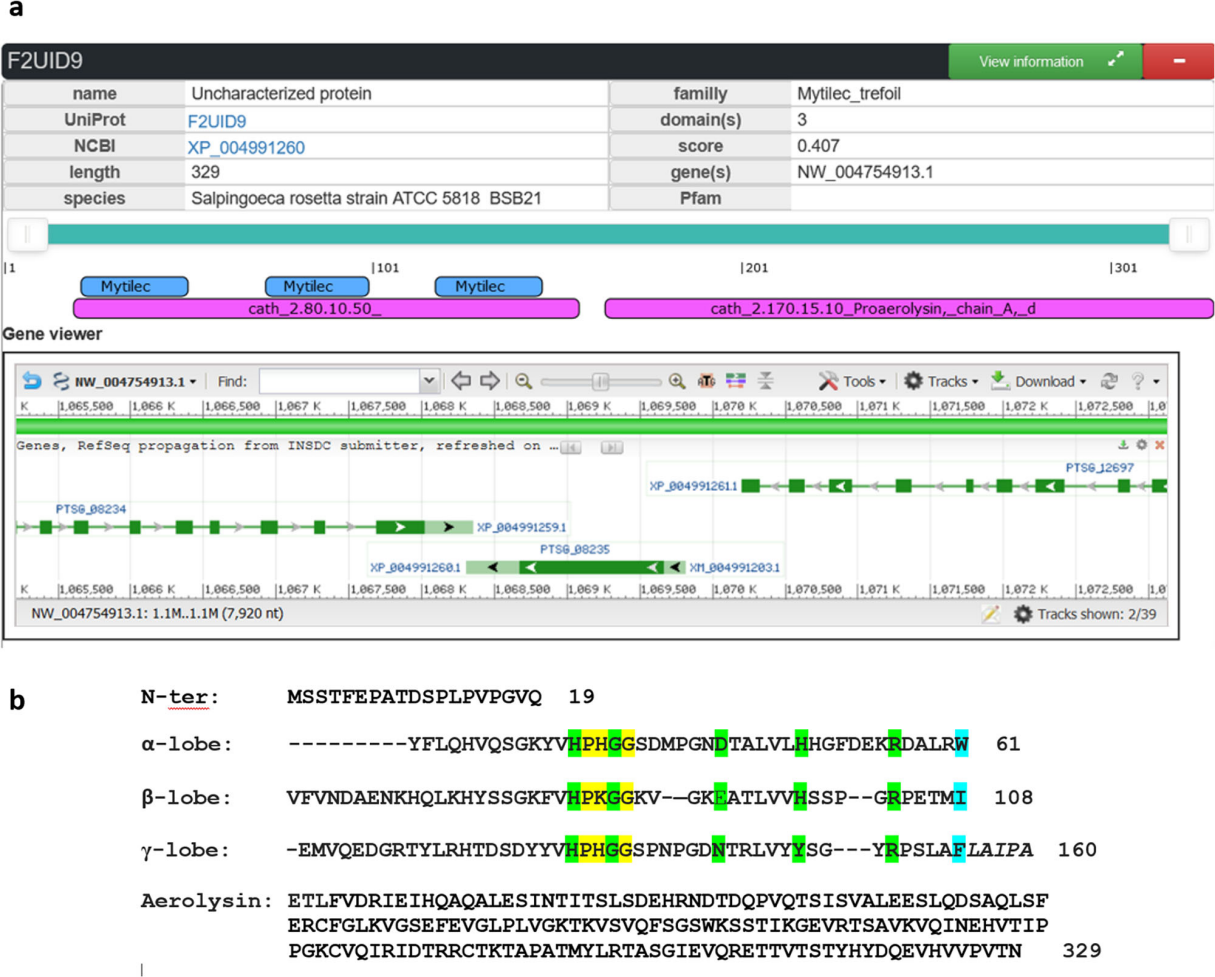
SaroL-1 sequence information. **a** Exerpt of the TrefLec page of the predicted lectin from *Salpingoeca rosetta* with information about the protein, the domains and the gene. **b** Peptide sequence of SaroL-1 with separation of the domains and alignment of lobes for the β-trefoil domain. Amino acids corresponding to the signature of Mytilec-like class are highlighted in yellow, amino acids predicted to be involved in carbohydrate binding in green, and amino acids involved in the hydrophobic core in blue.

Figure 2 depicts the TrefLec entry of the protein identified in *Salpingoeca rosetta* and referred here as SaroL-1. The sequence (F2UID9 in UniProt) consists of 329 amino acids. The 166 amino acid sequence at the *C*-terminus displays 30% identity with aerolysin domains in various organisms. Three repeats are located in the *N*-terminal region with 41% identity with the artificial Mytilec Mitsuba^24^ and 33% identity with other members of the Mytilec-like family from the Crenomytilus or Mytilus genera, with apparent conservation of the amino acids involved in carbohydrate binding.

### Binding of SaroL-1 lectin domain to αGal-containing glycoconjugates

The SaroL-1 gene was designed and fused with a 6-His-tag sequence and a cleavage site for Tobacco Etch Virus (TEV) protease at the *N*-terminus. The SaroL-1 protein was expressed in a soluble form in the BL21(DE3) strain of *Escherichia coli* and purified using immobilized metal ion affinity and size exclusion chromatography (Supplementary Fig. 3). Data provided by SEC-MALS and SDS PAGE analysis showed that SaroL-1 appears to be monomeric in solution with an estimated molecular weight of 36.86 ± 0.76 kDa.

**Fig. 3:**
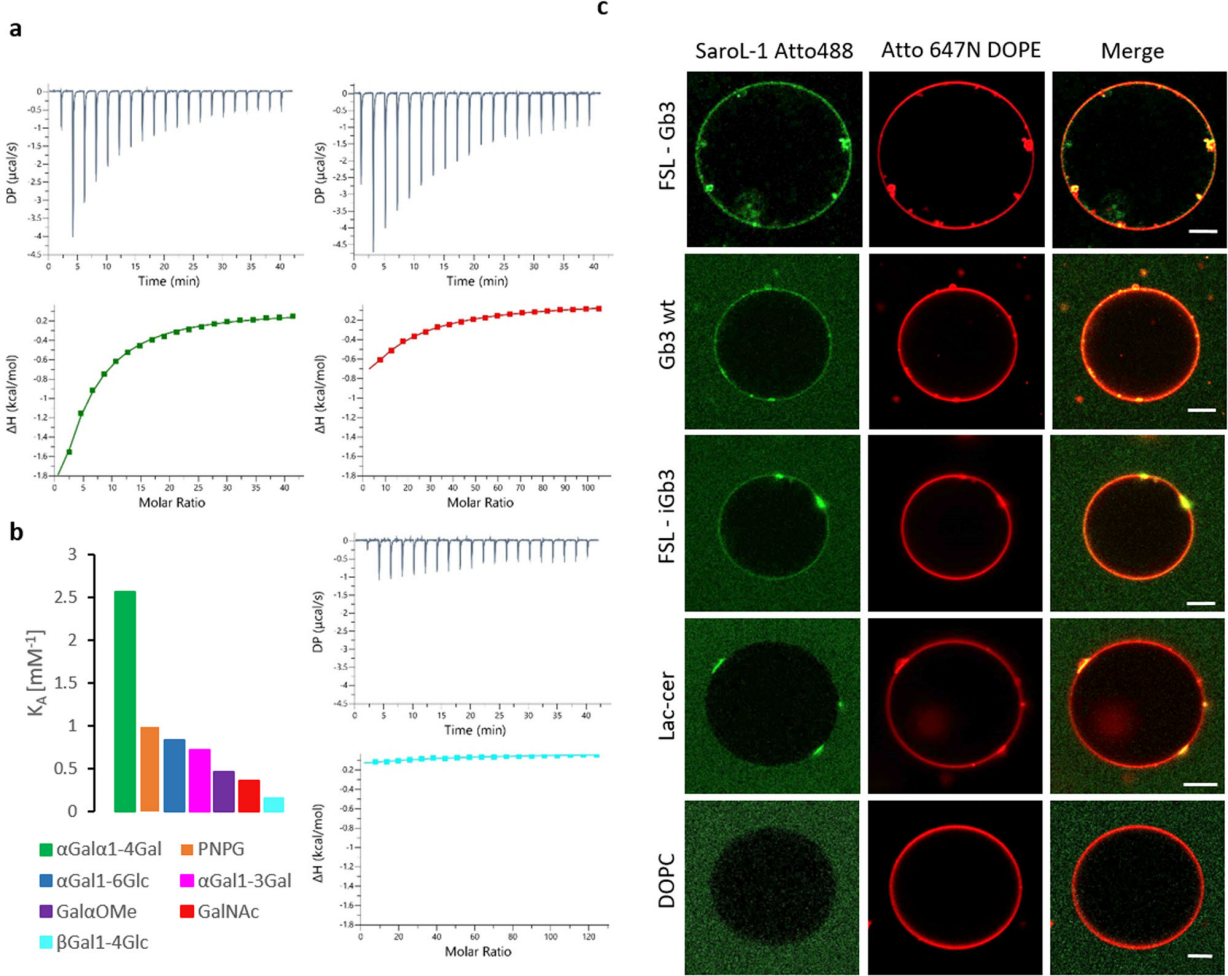
SaroL-1 recognizes αGal-containing ligands. **a** Representative ITC isotherms of SaroL-1 with αGal1-4Gal (green), GalNAc (red), and lactose (βGal1-4Glc) (cyan), **b** Comparison of K_A_ values of various binding partners for SaroL-1, **c** 200 nM of SaroL-1 (green) binds to GUVs (red; fluorescent lipid Atto 647 N) functionalised with either FSL-Gb3, Gb3 wt, FSL-iGb3, and lactosylceramide (Lac-cer). SaroL-1 induces tubular membrane invaginations in some cases, as visible for FSL-Gb3 GUVs and SaroL-1 clustering on GUVs, as visible for Gb3 wt, FSL-iGb3 and Lac-Cer GUVs. GUVs without functional group (DOPC) serves as negative control and show no binding of SaroL-1. The GUVs were composed of DOPC, cholesterol, glycolipid of choice, and membrane dye to the molar ratio of 64.7:30:5:0.3, respectively. Scale bars are 10 μm.

The binding of SaroL-1 to different galactosyl-ligands was assessed in solution by isothermal titration calorimetry (ITC). The monosaccharides *N*-acetylgalactosamine (GalNAc) and α-methyl galactoside (GalαOMe) displayed a similar millimolar affinity with a Kd of 2.2 and 2.8 mM, respectively. All tested αGal disaccharides and the p-nitrophenyl-α-D-galactopyranoside (PNPG) derivative bound with affinities twice as strong, with a Kd close to 1 mM, except for αGal1-4Gal, the terminal disaccharide of the globoside Gb3, that was characterized as the highest affinity ligand (Kd = 390 ± 0.20 μM). Lactose that contains βGal displayed very weak binding, being 20 times less efficient than αGal1-4Gal, confirming the preference for the αGal epitope (Fig. 3a, b). Affinity values and thermodynamics parameters are listed in Supplementary Table 2. ITC isotherms are shown in Fig. 3a and Supplementary Fig. 4.

The affinity of SaroL-1 to oligosaccharides in solution is not very strong but the avidity effect may result in much stronger binding to glycosylated surfaces. The ability of fluorescent-labelled SaroL-1 to bind multivalently to giant unilamellar vesicles (GUVs) with dimensions matching those of human cells^32^ was then evaluated by confocal imaging, using a fluorescent lipid as a membrane marker. The GUVs were functionalized with diverse naturally occurring glycolipids, including wild-type Gb3 mixture from porcine and lactosylceramide (Lac-cer), and several synthetic analogs consisting of the oligosaccharide attached to the phospholipid DOPE through a spacer molecule (Function-Spacer-Lipid, FSL). Strong binding of SaroL-1 was observed with GUVs containing FSL-Gb3 presenting the αGal1-4βGal1-4Glc trisaccharide (Fig. 3c). The binding of fluorescent SaroL-1 was of the same order as the one observed on GUVs containing natural wild-type Gb3 mixture from porcine, indicating the absence of effect of the artificial linker (Fig. 3c). In addition to binding to the surface of GUVs, SaroL-1 formed clusters, probably through multivalent recruitment of glycolipids, and membrane invaginations were observed in association with these clusters. These observations corroborate previous findings in other systems of multivalent lectins and glyco-decorated GUVs^33–36^ in which invaginations were proposed to be caused by glycolipid dynamics induced by the clustering of sugar heads. On the other hand, membrane invagination has been observed for other PFT, such as perforin, as a consequence of protein-protein interactions during the oligomerization process^37^.

SaroL-1 bound to a lesser extent to GUVs containing FSL-iGb3 that presents the αGal1-3βGal1-4Glc epitope. In this case, some clustering of SaroL-1 was also observed at the surface of the GUVs, but no membrane invaginations were formed. Finally, only very weak binding of labelled SaroL-1 was observed on lactosylceramide-containing GUVs (Fig. 3c) that present a βGal terminal sugar. No binding of SaroL-1 was observed to negative controls, namely DOPC GUVs without glycolipids (Fig. 3c) nor to GUVs decorated with the fucosylated oligosaccharides FSL-A and FSL-B, containing the blood group A and B trisaccharide, respectively (Supplementary Fig. 5). These oligosaccharides do have αGalNAc and αGal, respectively, but the presence of neighboring fucose prevented the binding by SaroL-1.

### Binding of SaroL-1 to H1299 cells

**Fig. 4:**
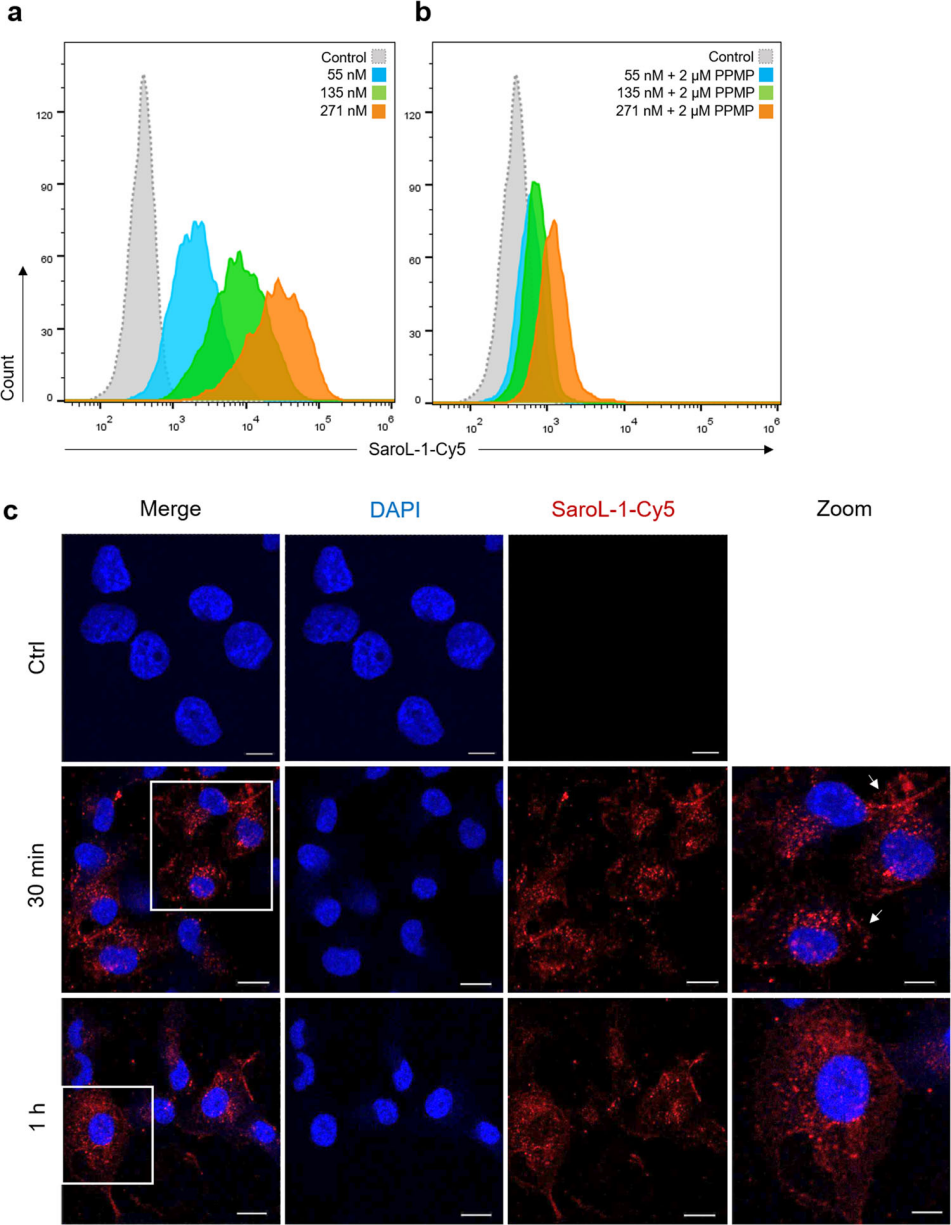
SaroL-1 shows dose-dependent binding and intracellular uptake into H1299 cells. **a** Histograms of fluorescence intensity of gated living H1299 cells incubated for 30 min at 4 °C with increasing concentrations of SaroL-1-Cy5 (grey, dotted histogram: negative control, blue: 55 nM, green: 135 nM, orange: 271 nm). Shifts in fluorescence intensity indicated that SaroL-1 binds to the H1299 cell surface in a dose-dependent manner. **b** Histogram of fluorescence intensity of gated living H1299 cells pre-treated for 72 h with the GSL synthesis inhibitor PPMP and incubated for 30 min at 4 °C with increasing concentrations of SaroL-1-Cy5 (grey, dotted histogram: negative control, blue: 55 nM, green: 135 nM, orange: 271 nm). In the absence of glucosylceramide-based GSLs, including Gb3, the binding of SaroL-1 to the plasma membrane was remarkably reduced. **c** Confocal imaging of H1299 human lung epithelial cells incubated with 271 nM Cy5-conjugated SaroL-1 (red) for indicated time points at 37 °C. The fluorescent signals accumulate partially at the plasma membrane and in the intracellular space of treated cells. Nuclei were counterstained by DAPI. Scale bars represent 10 μm.

The interactions of SaroL-1 with human cells were investigated on the human lung epithelial cell line H1299, a non-small cell lung cancer (NSCLC) characterized by increased cell surface Gb3 expression^38^. We monitored the binding of Cy5-labeled SaroL-1 (SaroL-1-Cy5) to cell surface receptors by flow cytometry (Fig. 4a, b) and its intracellular uptake by confocal imaging (Fig. 4c). In flow cytometry analysis, three different concentrations of SaroL-1 were applied (55, 135 and 271 nM). Cells were incubated with SaroL-1-Cy5 for 30 min on ice, then the unbound lectin was washed away to reduce unspecific signal, and fluorescence intensity was measured directly with FACS Gallios. The flow cytometry analysis revealed a strong binding of the protein to the cell surface in a dose-dependent manner. Figure 4a shows a remarkable shift in fluorescence intensity for the samples treated with all concentrations of SaroL-1 (blue, green and orange histograms) compared to the negative control (grey, dotted histogram) after 30 min of incubation without reaching signal saturation.

To inhibit the conversion of ceramide to glucosylceramide and accordingly the subsequent biosynthesis of Gb3, H1299 were incubated with PPMP, a chemical inhibitor of glucosylceramide synthase (GCS) activity, used to deplete Gb3 expression. H1299 cells were incubated with 2 µM PPMP for 72 h before flow cytometry analysis. Subsequently, cells were treated with 55 nM, 135 nM or 271 nM of fluorescently labeled SaroL-1 (SaroL-1-Cy5) for 30 min on ice (Fig. 4b) and samples were analyzed as described above. Histograms of fluorescence intensities revealed a significant reduction in SaroL-1 binding to the plasma membrane compared to Fig. 4a for all tested concentrations. These results suggest a crucial role of the glycosphingolipid Gb3 as a cell surface receptor for SaroL-1.

Subsequently, H1299 cells were imaged with confocal microscopy to investigate the intracellular uptake of SaroL-1. For these experiments, the concentration of SaroL-1 was set to 271 nM to improve fluorescence signal intensity and image quality. After 30 min and 1 h of incubation at 37 °C (Fig. 4c), the internalization of SaroL-1-Cy5 into H1299 cells became visible, as shown by the fluorescent signal in red. SaroL-1 seemed widely distributed in the intracellular environment and at the plasma membrane (as indicated by white arrows) at both time points. In conclusion, our observations confirm that SaroL-1 1 binds to Gb3 receptors exposed at the plasma membrane and induces its intracellular uptake.

### Crystal structure of SaroL-1 in complex with ligands

Crystallization experiments were performed with SaroL-1 in complex with different αGal- and GalNAc-containing mono-, di- and tri-saccharides. Several crystals were obtained, those of the complex SaroL-1/GalNAc and SaroL-1/Gb3 trisaccharide showed suitable diffraction and datasets were collected at 1.7 Å and 1.8 Å, respectively (Table 1). Attempts to solve the crystal structures by molecular replacement were unsuccessful. Thus the selenium-methionine variant of SaroL-1 was expressed, purified, and crystallized for experimental determination of the phases. A multi-wavelength anomalous diffraction (MAD) dataset was collected at 2.3 Å and used to solve and refine the structure of SeMet SaroL-1 (Table 1) (PDB code 7QE3). The latter was subsequently used to solve the structure of the complexes of SaroL-1 with GalNAc (PDB code 7QE4) and the Gb3 trisaccharide (PDB code 7R55) by molecular replacement.

**Table 1:** Data collection and refinement statistics for SaroL-1/GalNAc, SaroL-1/Gb3 and Se-M SaroL-1/GalNAc.

Protein name	SaroL-1/GalNAc		SaroL-1/Gb3		Se-M-SaroL-1	
Data collection						
Beamline	Soleil Synchrotron Proxima-2		Soleil Synchrotron Proxima-1		Soleil Synchrotron Proxima-2	
Wavelength	0.980107		0.978565		0.979415	
Space group	P2_1_2_1_2_1_		P2_1_2_1_2_1_		P2_1_	
Unit cell dimensions	57.16 59.25 209.61 90.00 90.00 90.00		57.34 58.99 210.30 90.00 90.00 90.00		44.99 58.58 166.42 90.00 90.68 90.00	
Resolution (Å)	45.19 – 1.70		45.18 – 1.84		39.78 – 2.20	
*R*_merge_	0.078 (0.616)		0.093 (1.034)		0.095 (0.503)	
*R*_*pim*_	0.041 (0.339)		0.050 (0.572)		0.072 (0.383)	
*Mean I*/σ*I*	14.6 (3.1)		11.8 (1.7)		11.2 (3.0)	
Completeness (%)	100 (100)		99.8 (96.9)		99.5 (98.5)	
Redundancy	8.5 (8.3)		8.2 (7.8)		4.9 (5.0)	
CC1/2	0.999 (0.890)		0.999 (0.792)		0.996 (0.852)	
Nb reflections	678912 (34686)		515983 (28809)		215732 (18811)	
Nb unique reflections	79471 (4185)		62904 (3696)		44177 (3794)	
Refinement						
Resolution (Å)	44.28 – 1.70		45.14 – 1.84		39.77 - 2.20	
No. reflections No. free reflections	75465 3914		59836 2982		42022 2095	
*R*_work_/*R*_free_	0.169/0.206		0.184/0.240		0.189/0.231	
R.m.s Bond lengths (Å)	0.008		0.018		0.014	
Rmsd Bond angles (°)	1.433		2.201		1.920	
Rmsd Chiral (Å^3^)	0.007		0.109		0.011	
Clashscore	2		2		4	
No. atoms/Bfac (Å^2^) Protein Sugar Water	Chain A 5095/20.7 120/18.9 486/31.3	Chain B 5115/19.0 120/21.0 542/30.0	Chain A 2543/28.7 68/34.2 331/35.9	Chain B 2527/30.9 68/33.5 249/36.3	Chain A 2481/38.0 15/42.58 191/40.1	Chain B 2482/29.5 - 280/34.3
Ramachandran Allowed/Favored/Outliers (%)	98.6 97.8 0.00		98.1 97.0 0.16		97.9 96.4 0.00	
PDB Code	7QE4		7R55		7QE3	

*Values in brackets are for highest-resolution shell.

**Riding hydrogen atoms were added to the coordinate file of 7QE4.

**Fig. 5:**
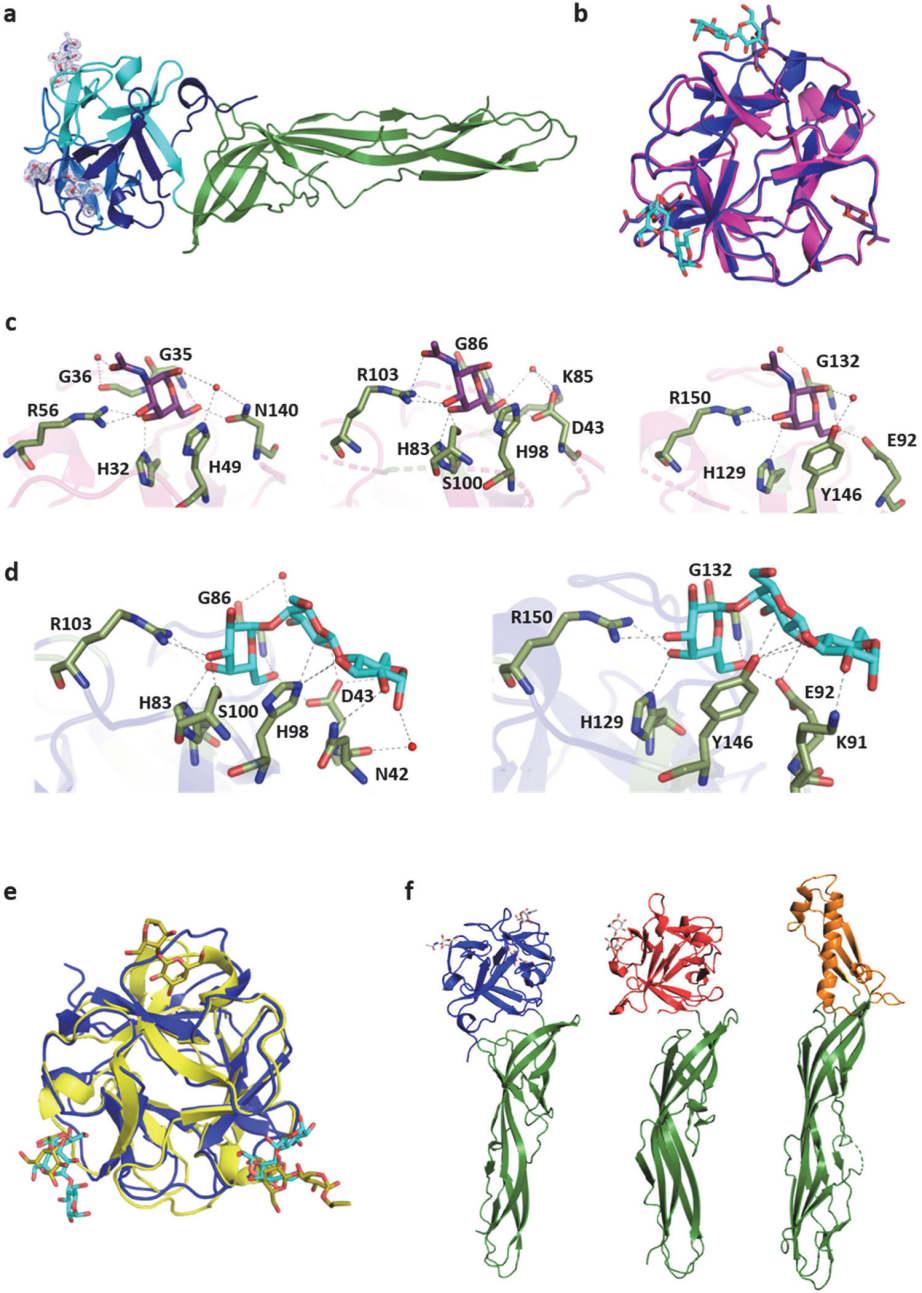
Crystal structure of SaroL-1. **a** Cartoon representation of monomeric SaroL-1 in complex with GalNAc. β-trefoil-domain colored in blue and aerolysin domain in green. The GalNAc ligands are displayed in their electron density map as sticks. **b** Superimposition of β-trefoil lectin domains, (7QE4, light magenta), (7R55, blue) in complex with 3 molecules of GalNAc (violet) and 2 molecules of Gb3 (cyan). **c** Zoom on α, β and γ binding sites with GalNAc (violet) polar contacts are represented as dashed lines and bridging water molecules as red spheres. **d** Zoom on the interactions with Gb3 (cyan) in binding β and γ sites, polar contacts are represented as dashed lines and bridging water molecules as red spheres. **e** Overlay of β-trefoil domains of SaroL-1 (blue) in complex with Gb3 (cyan) and of monomeric CGL (5F90) (yellow) in complex with Gb3 and αGal1-4Gal (yellow). **f** Comparison of the structures of monomeric SaroL-1, pore-forming lectin LSL (1W3A) and ε-toxin (1UYJ) from left to right. The pro-aerolysin domain is colored in green and the membrane-binding domain in blue (SaroL-1), red (LSL) and orange (ε-toxin).

The overall structure of SaroL-1/GalNAc consists of two monomers in the asymmetric unit. They are highly similar (RMSD = 0.52 Å) and do not present extensive contact, confirming the monomeric state of the lectin in solution. SaroL-1 is composed of two domains, a β-trefoil domain at the *N*-terminal region shown in blue and an elongated domain (green) consisting of seven β-strands forming a twisted β-sheet (Fig. 5a). Clear electron density for three GalNAc monosaccharides is observed in the lectin domain, corresponding to the three sites α, β and γ, classically observed in the β-trefoil structure (Supplementary Fig. 6). In all sites, both α- and β-anomers of the GalNAc monosaccharides are present, with stronger occupancy for the α anomer. The three binding sites share strong sequence similarities, albeit with some variations in the amino acids and contacts (Fig. 5c and Supplementary Table 3). The axial O4 of αGalNAc establishes hydrogen bonds with the side chain of conserved His and Arg residues in all sites. This Arg also interacts with the O3 hydroxyl establishing fork-like contacts with two adjacent oxygens of the sugar ring. The O6 hydroxyl is hydrogen-bonded to the main chain of a conserved Gly residue and to the side chain oxygen of a more variable Asn/Asp/Glu residue. Several water molecules are also involved in bridging the protein and the carbohydrate. Moreover, each GalNAc is stabilized in the binding pocket by a C-π-stacking interaction between its hydrophobic face and the aromatic ring of His (site α and β) or Tyr (site γ). The N-acetyl group of GalNAc establishes mostly water-mediated contacts, confirming that it is not crucial for affinity.

The complex of SaroL-1 with the Gb3 trisaccharide presents the same packing as the one with GalNAc. The protein structure is also similar in both complexes albeit with a variation in the fold of the aerolysin β-sheets that present a small kink in its medium region, resulting in an angular deviation of ~17° (Supplementary Fig. 7e). The terminal α-galactose of the trisaccharide occupies the exact location and establishes the same contact as GalNAc in the other complex. Electron density was observed in binding sites β and γ only (Fig. 5b and Supplementary Fig. 6). Site α is not occupied, presumably because of close proximity with the neighboring monomer in the crystal. This packing effect also explains the small kink observed in the aerolysin β-sheet. The presence of ligand in site α in the complex with GalNAc, but not in the complex with Gb3, created steric hindrance with the close neighboring monomer, therefore inducing a slightly different conformation (Supplementary Fig. 7f). The second galactose residue (βGal) is perpendicular to His98 in site β and Tyr146 in site γ with hydrogen bonds involving ring oxygen O5 of galactose (Fig. 5d). In both cases, an acidic amino acid (Asp43 in site β and Glu92 in site γ) bridges between the two Gal residues by establishing two strong hydrogen bonds between O6 of αGal and O2 of βGal. Finally, the reducing Glc also participates in the hydrogen bond network through hydroxyl O2 interacting with Asn42 (site β) or Lys91 (site γ). Several bridging water molecules are also involved in the binding network.

The β-trefoil domain of SaroL-1 belongs to the Mytilec-like class of lectins and the above-cited His and Gly belong to the HPXGG sequence motif conserved in this class (Fig. 2b)^23^. The *N*-terminal domain of SaroL-1 demonstrates strong structural similarity with the β-trefoil of the lectins from *Mytilus galloprovincialis* (Mytilec)^23^, *Crenomytilus grayanus* (CGL)^22^ and synthetic construct Mitsuba^24^ with a sequence identity of 34%, 33% and 41%, and RMSD = 0.66 Å, 0.69 Å and 0.81 Å, respectively (Fig. 5e and Supplementary Fig. 7a–d). The CGL structure has been obtained in a complex with Gb3 with the trisaccharide fully visible only in one of the three binding sites. The location of the terminal αGal is similar in CGL and SaroL-1, while the other part of the trisaccharide shows a significant variation as demonstrated in the superimposition of SaroL-1 and CGL complexes (Fig. 5e).

Although the sequence of the *C*-terminal elongated β-sheet domain of SaroL-1 has no similarity to those from known structures, its structure is very similar to the pore-forming region of the aerolysin-type β-PFTs, such as LSL a *Laetiporus sulphureus* lectin^14^ and the ε-toxin of *Clostridium perfringens*^39,40^ (Fig. 5f). However, sequence identities are about 20%. In its pro-aerolysin state, i.e., the solution state, the β-PFT fold is characterized by an extended shape consisting of long and short β-strands, creating two main domains^41^.

### Pore-forming property of SaroL-1

**Fig. 6:**
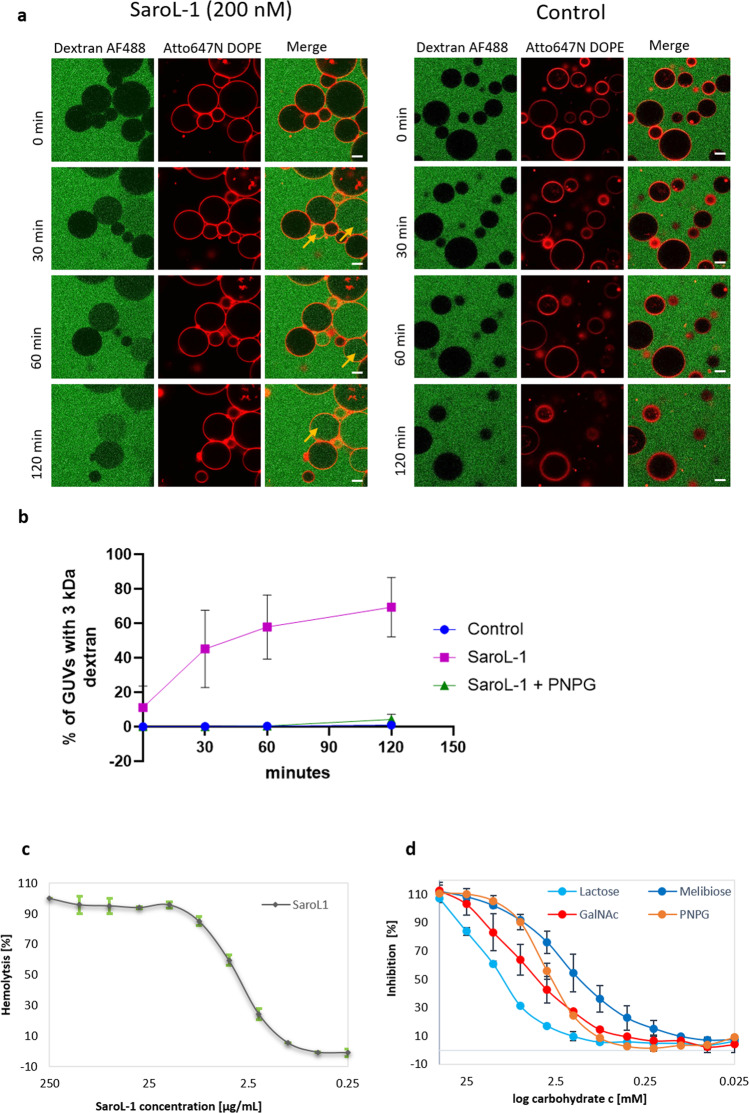
Pore-forming and hemolytic activity of SaroL-1. **a** SaroL-1 (unlabeled, 200 nM) triggers the influx of 3 kDa dextran-AF488 (green) into wt Gb3-containing GUVs (red) via its pore-forming activity. In the control group without SaroL-1, there was no visible influx of dextran-AF488 detected. Yellow arrows indicate events of dextran-AF488 influx to wt Gb3-containing GUVs. The GUVs were composed of DOPC, cholesterol, wt Gb3, and membrane dye to the molar ratio of 64.7:30:5:0.3, respectively. The scale bars represent 10 µm. **b** Kinetics of SaroL-1 driven dextran-AF488 influx to wt Gb3-containing GUVs. Mean values ± SD are shown. Data represent three independent experiments, *n* = 3. The molecular weight of fluorescently labelled dextran is 3 kDa. The total amount of control GUVs was at 0 min—225 GUVs, 30 min—344 GUVs, 60 min—349 GUVs and 120 min—393 GUVs. For SaroL-1 experiment with wt Gb3-containing GUVs, the total amount of GUVs was 0 min—230 GUVs, 30 min—185 GUVs, 60 min—183 GUVs and 120 min—178 GUVs. In the PNPG-treated group there was a total amount of GUVs at 0 min—322 GUVs, at 30 min—435 GUVs, 60 min—447 GUVs and at 120 min 446 GUVs. **c** Relative hemolytic activity of SaroL-1 with estimation of IC_50_ as 6.3 μg/mL (170 nM). Mean values ± SD are shown. Data represent two independent experiments, *n* = 2. **d** Relative inhibition of the hemolytic activity of SaroL-1 by PNPG, GalNAc, melibiose and lactose. Mean values ± SD are shown. Data represent two independent experiments, *n* = 2. All error bars correspond to mean value ± SD. Data for the graphs are available in Supplementary Data 1.

The presence of the hemolytic/pore-forming domain indicates that SaroL-1 could form pore-like structures upon membrane binding, which would fit with our first observation of the alteration of glycolipid dynamics described above. To test our hypothesis of pore-formation, we incubated wt Gb3-containing GUVs (indicated by the fluorescent lipid DOPE-Alexa647N; red color) with 200 nM unlabeled SaroL-1 and 3 kDa dextran labeled by Alexa Fluor ™ 488 (dextran-AF488; green color) and monitored dextran influx into GUVs for 2 h by using confocal microscopy (Fig. 6a). After 30 min of incubation, 45% of the 185 total observed GUVs in the experiments were filled with dextran-AF488. The number of GUVs filled with dextran-AF488 steadily increased over time and reached 69% out of 178 GUVs after 2 h of incubation with SaroL-1 (Fig. 6a). The control group of wt Gb3-containing GUVs incubated together with dextran-AF488 but without SaroL-1 showed <1% influx of dextran of total 393 GUVs after 2 h. Similarly, we incubated wt Gb3-containing GUVs with 200 nM SaroL-1 and fluorescently labelled 70 kDa FITC-dextran (Supplementary Fig. 8). The larger polysaccharides also entered in the GUVs but with a different kinetic. After 30 min of incubation, only 17% of total observed 211 GUVs were filled with 70 kDa dextran. The influx of dextran inside of GUVs increased over the time but at 60 and 120 min only 29% of total observed GUVs were filled with 70 kDa dextran. The size of 3 kDa fluorescently labeled dextran corresponds to a hydrodynamic radius of 18 Å^42^ whereas the hydrodynamic radius of 70 kDa fluorescently labeled dextran is 72 Å^43^. The pore diameter range of aerolysin-like toxins is in the range of 1–4 nm^44^, therefore consistent with the observed influx of 3 kDa dextran entering in the GUVs. On the other hand, slower equilibration of 70 kDa dextran inside of GUVs can be explained by the fact that dextran as polysaccharide can adopt an extended conformation and enter the pore however with significant delay.

The role of the lectin domain in pore formation was investigated by adding the soluble galactose analog PNPG that competes with Gb3 in the binding site. We pre-treated 200 nM SaroL-1 with 10 mM PNPG for 15 min at RT in PBS buffer and then added the solution to wt Gb3-containing GUVs. In the PNPG-treated group, the influx of dextran was fully inhibited for the first 30 min, as none of 435 of total GUVs were filled with dextran. However, after 2 h, 4% of total 466 GUVs were filled with dextran (Fig. 6b). Therefore, it was demonstrated that the Gb3-binding activity of the lectin domain is necessary for pore formation. Obtained results indicate that SaroL-1 plays a role in dextran-AF488 influx to wt Gb3-containing GUVs compared to the negative control and prefers the glycosphingolipid Gb3 over PNPG.

When adding SaroL-1 to rabbit red blood cells, hemolysis was observed instead of hemagglutination classically induced by multivalent lectins^45^. The hemolysis was confirmed by measuring the absorbance at 540 nm (Fig. 6c). SaroL-1 appeared to be a potent hemolytic agent since a concentration of 27 nM is sufficient for damaging erythrocytes, whereas nearly complete hemolysis was observed at a concentration of 8 μg/mL (217 nM). Analysis of the red blood cells by microscopy demonstrated the occurrence of almond-like shaped erythrocytes after a few minutes of exposition to the lectin (Supplementary Fig. 9). The number of erythrocytes decreases with increasing incubation time as cells are possibly lysed due to morphological changes. The role of the lectin domain in the pore formation was assayed by pre-incubating SaroL-1 with several carbohydrates in different concentrations before measuring the hemolysis. Melibiose and PNPG appeared as the most efficient competitors (Fig. 6d) with complete inhibition of hemolysis at 10 mM (IC_50_ (melibiose) = 1.5 mM, IC_50_ (PNPG) = 2.9 mM). In agreement with ITC and X-rays data, GalNAc was also an efficient inhibitor (IC_50_ = 4.9 mM), while lactose action was weaker (IC_50_ = 11.9 mM).

### Toxicity of SaroL-1 towards cancer cells

**Fig. 7:**
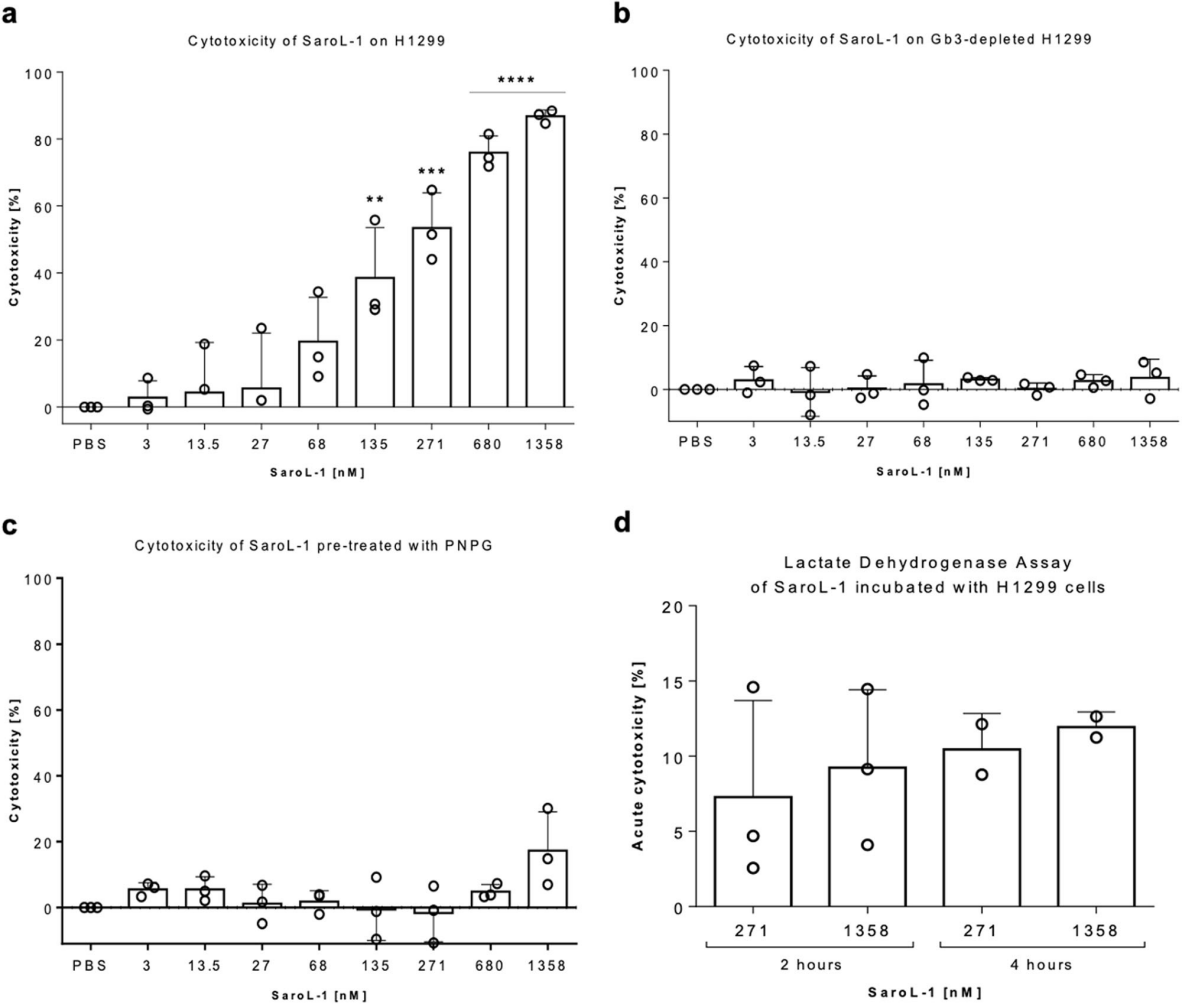
Cytotoxic activity of SaroL-1 against H1299 cells. **a** Dose-dependent increase of cytotoxicity following the addition of purified SaroL-1 in a standard cell proliferation assay (MTT) demonstrating increment of cytotoxicity after 24 h of incubation compared to treatment with PBS. Cell viability is reduced by ~87% after stimulation with 1.36 µM SaroL-1. Data represent three independent experiments, *n* = 3. **b** Cell proliferation assay (MTT) of H1299 cells pre-treated with PPMP for 72 h before addition of increasing concentrations of purified SaroL-1. Cytotoxicity was remarkably reduced after 24 h of incubation with SaroL-1 in absence of the glycosphingolipid Gb3 at the plasma membrane of treated cells. Data represent three independent experiments, *n* = 3. **c** The soluble sugar PNPG inhibited SaroL-1 cytotoxicity. H1299 were incubated with increasing concentrations of SaroL-1 pre-treated with 10 mM PNPG. Cell proliferation assay (MTT) was used to assess SaroL-1´s cytotoxic activity after 24 h in comparison to the treatment with PBS. The results indicate that cell viability is preserved when SaroL-1 glycan-binding sites are saturated with soluble 10 mM PNPG. Data represent three independent experiments, *n* = 3. **d** H1299 cells suffer of acute cytotoxicity and membrane damage in presence of SaroL-1. A lactate dehydrogenase (LDH) assay revealed impairment of cell membrane integrity upon incubation with 0.27 µM and 1.36 µM SaroL-1 after 2 and 4 h. Data represent two independent experiments, *n* = 2. Differences to the control were analyzed for significance by using two-tailed unpaired *t*-test. *p < 0.05, **p < 0.01, ***p < 0.001, ****p < 0.0001.

As the aerolysin domain may cause osmotic lysis and cell death^41^, we determined the cytotoxic effect of SaroL-1 on H1299 cells after 24 h treatment using a cell proliferation assay (MTT). The assay is based on the cleavage of tetrazolium salt MTT to form a formazan dye by metabolic-active cells, suitable for quantifying cell proliferation and viability. SaroL-1 shows cytotoxic activity in fetal calf serum (FCS)-containing medium, and the percentage of viable cells after 24 h decreased by half at a concentration of 271 nM. Based on the results depicted in Fig. 7a, increasing concentrations of SaroL-1 decreased the proliferation and viability of human epithelial cells in vitro in a dose-dependent manner. No significant cytotoxicity was observed upon treatment with PBS as negative control.

Additionally, we depleted glucosylceramide-based glycosphingolipids from H1299 cells (Fig. 7b) and saturated the carbohydrate-binding pockets of SaroL-1 with PNPG (Fig. 7c) to prove that a glycan-driven binding and internalization of the protein is essential to exert its cytotoxicity on cells. Figure 7b shows a standard MTT assay with H1299 cells pre-treated for 72 h with the GSL synthase inhibitor PPMP, also for the depletion of Gb3 at the membrane. In the absence of Gb3, the percentage of cytotoxicity following SaroL-1 addition to cells was remarkably reduced, and H1299 preserved their viability after 24 h incubation with the protein. Similarly, in a second experiment, increasing concentrations of the lectin (3, 14, 27, 68, 135, 271, 680, 1358 nM) were pre-incubated with 10 mM PNPG for 15 min at RT, then the solution was added to cells in a standard MTT assay. Strikingly, in the presence of PNPG, cell death was largely reduced by >90% after 24 h of incubation (Fig. 7c). Treated cells preserved viability, even in the presence of high protein concentrations. These results indicate that SaroL-1 activity is efficiently inhibited by 10 mM PNPG, leading to a substantial decrease in cell cytotoxicity. Remarkably, we demonstrate that SaroL-1 can exert cytotoxic activity on H1299 cells only upon binding to glycosylated receptors exposed at the plasma membrane.

Moreover, we investigated the formation of holes in the plasma membrane of H1299 cells induced by SaroL-1 after 2 and 4 h. To this end, a lactate dehydrogenase (LDH) assay was used to assess rapid changes in cell viability and membrane integrity of treated cells. Indeed, LDH is considered a ubiquitous enzyme rapidly released from the membrane of cells impaired by stress, chemicals or injuries. The amount of LDH released from cells can therefore be used to assess cellular damage and cell death. As depicted in Fig. 7d, 271 nM and 1.36 µM SaroL-1 induced acute cytotoxicity in H1299 after short incubation times (2 and 4 h). The detection of extracellular LDH following SaroL-1 addition to cells indicate the ability of this protein to induce pores at the membrane which lead to the release of significant LDH amounts at 4 h, and ultimately to cell death at 24 h.

Undeniably, cell anchorage not only provides the structural support for a cell, but it mediates crucial survival signals for the cells, providing access to nutrients and growth factors. Furthermore, it has been established that processes that modify cell adhesion leading to the loss of cell anchorage may induce cell death^46^. We therefore investigated the possible role of SaroL-1´s hemolytic domain in the disruption of cell adhesion. We proved that SaroL-1, mainly upon binding to the Gb3 receptor, causes a dose-dependent rounding and detachment of cells compared to PBS-treated cells, ultimately leading to cell death (Supplementary Fig. 10).

### Assembly of SaroL-1 in transmembrane pore structures

**Fig. 8:**
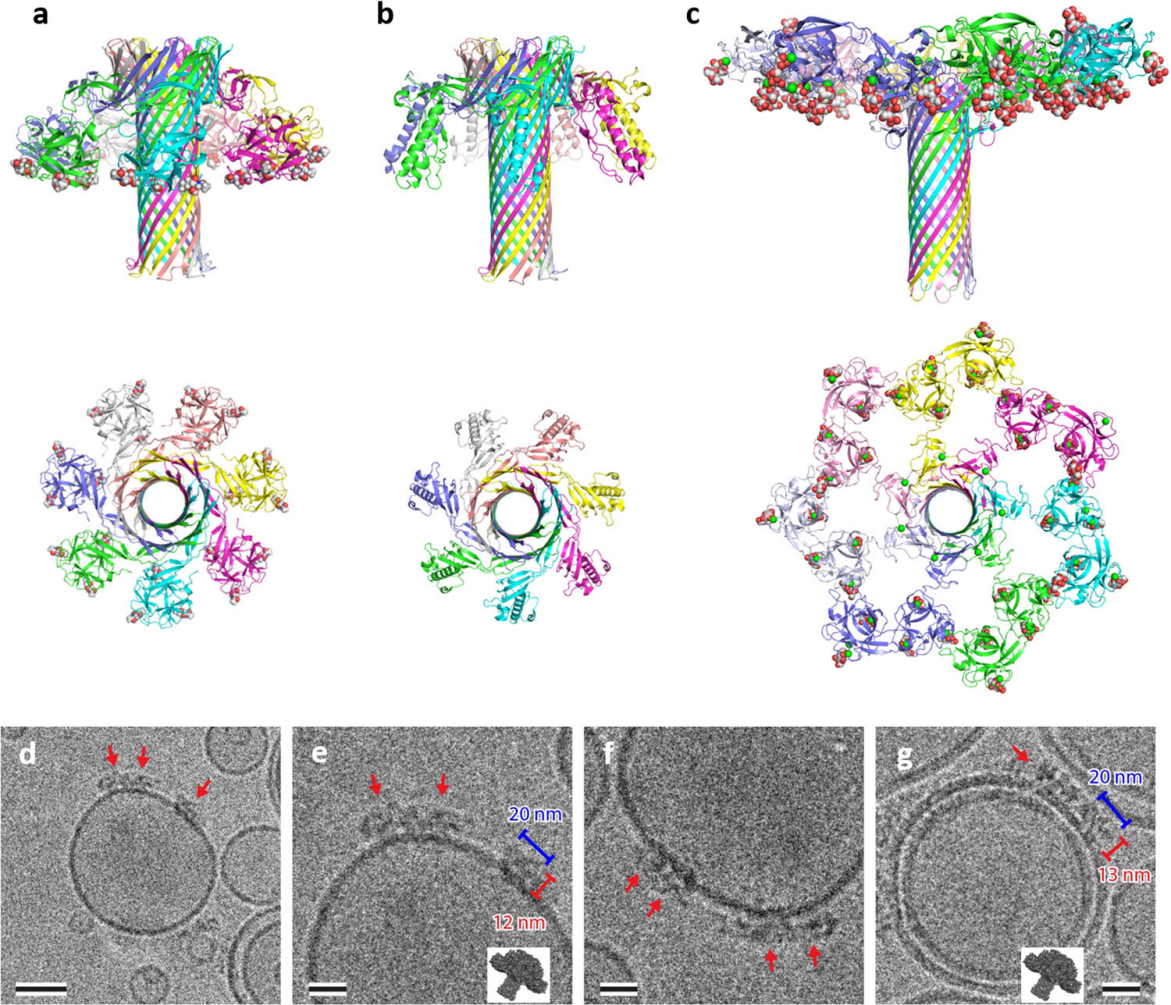
Prediction and visualization of Sarol-1 pore oligomeric assembly. **a** Preliminary model of membrane-bound heptameric SaroL-1 built by homology modeling using the structure similarity displayed in Fig. 3. **b** Crystal structure of heptameric ε-toxin of C. perfringens (PDB 6RB9). **c** Crystal structure of heptameric lactose-binding lectin CEL-III from *Cucumaria echinata* complexed with βGal derivative (PDB 3WT9). **d**–**g** Cryo TEM images of SaroL-1 clusters bound to Gb3-decorated LUVs. Red arrows indicate mushroom-like oligomers of SaroL-1 with estimated sizes (indicated by blue and red scale bars, respectively) corresponding to the heptameric predicted model (grey surface miniature). Scale bars are 50 nm in **d** and 20 nm in **e**–**g**.

β-PFTs of the aerolysin-family occur as a monomer in solution and form pores in membranes according to the following steps: they bind to a cell-surface receptor, oligomerise while generating β-hairpins from each of six to seven individual monomers and then, produce a vertical β-stranded pore which varies in size from 20 to 25 Å. to visualize the relative orientation of the lectin- and pore-forming domain of SaroL-1 in the pore architecture, a 3D model was built, using a template selected from crystal structures of a toxin for which both solution-monomeric and pore-heptameric data were available. The ε-toxin of *C. perfringens*^39,40^ matched the requirements. Although the primary sequence identity of the pore-forming domains of SaroL-1 and ε-toxin (PDB 1UYJ) is low (12%), the 3D-structures of the monomeric state are strikingly similar (Fig. 5e) and were used for the template alignment (Supplementary Fig. 11). From this, a heptameric SaroL-1 pore was built with SwissModel^47^ based on the membrane pore structure of ε-toxin (PDB 6RB9) (Fig. 8b). The lectin domain was then linked on the *N*-terminal extremity of each hairpin, in a conformation bringing all carbohydrate-binding sites towards the surface of the membrane.

The resulting preliminary model (Fig. 8a) confirms that the lectin domain can adopt an orientation that locates all 21 αGal binding sites of the heptamer in a plane, which would correspond to the surface of the glycosylated host cell membrane. The model can be compared to the only structure of a pore formed by oligomerization of an aerolysin-associated lectin. CEL-III from sea cucumber (Fig. 8c) forms the same type of heptameric pore, with all 35 carbohydrate-binding sites oriented towards the cell surface^13^. The position of lectin domains is however different in CEL-III structure and in the present model. Indeed, extensive molecular dynamics studies would be necessary to explore all possible orientations of the lectin domain, as well as the molecular mechanisms occurring during pore-formation, as previously performed for aerolysin^41^.

The assembly of pore structures in membranes was confirmed by cryogenic transmission electron microscopy. SaroL-1 (9.2 μM) was pre-incubated with Gb3-decorated large unilamellar vesicles (LUVs) and subsequently observed under cryogenic condition. Different types of oligomerization or aggregation were observed. In a dense LUV solution, SaroL-1 oligomerized while crosslinking liposome surfaces, probably through Gb3 binding, while insertion in the membrane was also observed (Supplementary Fig. 12a). When LUVs were more diluted and could be observed as single objects, the protein oligomerized as patches at the surface, with insertion into the membrane (Fig. 8d–g). Individual mushroom-like structures can be visualized at the surface of the LUVs, corresponding to the shape and size of the model of Fig. 8a. The tube part of the pores visibly integrated into lipid membrane of the vesicles. The dimensions of the observed mushroom-type structures are in the range of 13-20 nm, which corresponds to the heptameric organization of the predicted model of the pore.

## Discussion

Lectins have been primarily studied for their ability to bind to complex carbohydrates, with applications in biotechnology, histology, and diagnostics^48,49^. Their role in signaling has been recently emphasized due to their ability to cross-link membrane glycoproteins and glycolipids^36^. The interest is now high for identifying lectins associated with an additional domain carrying a particular function that can be specifically targeted to glycan-bearing cells. Furthermore, the development in protein engineering and synthetic biology highlights lectins and carbohydrate-binding modules as building blocks in designing novel molecules such as artificial and multivalent dimers increasing affinity to sialic acid^50^ or Janus lectins that associate two domains with different carbohydrate specificity^51,52^. In this context, β-trefoil lectins are pretty attractive: they are small and compact, very stable due to their hydrophobic core and multivalent through tandem repeats, thereby increasing avidity for their substrates.

β-trefoil lectins can be engineered for adapting their specificity to a precise glycan target^53^ or enhancing their internal symmetry^24^. We constructed the TrefLec database that contains predicted sequences for each of the 12 β-trefoil lectin classes, resulting from mining translated genomes. This resource offers the option of searching the tens of thousands of β-trefoil candidates for new lectin sequences and possibly associated with the additional functional domain(s). This approach was validated by identifying Mytilec family members previously found only in mollusks and other marine animals, for instance, in the lower eukaryote *Salpingoeca rosetta*. A new Mytilec sequence was selected from TrefLec, consisting of a Mytilec-like and an aerolysin domain despite the poorly informative initial UniProt record mentioning an uncharacterized protein.

The identification of a novel β-PFT with putative membrane-binding domain specific for the αGal epitope was completed by compelling evidence of carbohydrate-mediated interactions. Indeed, β-PFT aerolysin-like toxins have a conserved pore-forming architecture and a large variety of membrane-binding domains. *Aeromonas* aerolysin binds to a glycosylphosphatidylinositol-anchored protein^54^, and the *Clostridium* ε-toxin binds to proteins associated with lipid rafts^55^. Several β-PFTs have a lectin-type membrane-binding domain, such as CEL-III from sea cucumber, including 35 binding sites for galactose in its hemolytic heptameric form^13^. The fungal β-PFT from *L. sulfureus* has a lectin domain specific for lactose that also occurs in many other fungi^56^. Plant β-trefoil lectins of the Amaranthin class were also predicted to be associated with aerolysin in many plants^28^. While bacteria use PFT as virulence factors, these proteins are generally involved in defense response in eukaryotes, with anti-feeder properties in plants and fungi, and anti-infectious properties in animals. Lectins are involved in innate immunity^57^ and therefore can play a role in such defense mechanisms. Interestingly, PFTs with confirmed anti-infectious functions, such as biomphalysin from the snail *Biomphalaria glabrata*^58^ and βγ-CAT from the frog *Bombina maxima*^59^, contain lectin-like domains (fibrinogen and trefoil factor, respectively) although their ligand is yet to be identified. The physiological role of SaroL-1 in Choanoflagellates is therefore probably related to primitive innate immunity.

SaroL-1 is the first identified β-PFT with a lectin-type membrane-binding domain specific for an epitope associated with several cancers. Its specificity and biological activity were established with different biophysical and cell biological approaches. SaroL-1 showed preferences for α-galactosides with much stronger affinity than for β-galactosides, such as lactose. The specific binding to the glycosphingolipid Gb3, a cancer biomarker detected on Burkitt’s lymphoma^60^, ovarian^61^, colorectal^62^, breast^63^ and pancreatic^64^ cancers, is of particular interest. As recently reviewed^65^, several lectins have been identified as being specific to Gb3. Therefore, we tested SaroL-1 on the Gb3-positive H1299 lung epithelial cell line and observed binding and intercellular uptake. However, the binding was vastly diminished on Gb3-depleted cells pointing to a Gb3-dependent mode of interaction. Some Mytilec-like lectins, such as CGL^22,30^ and Mytilec^66^ were shown to be cytotoxic to certain cancer cell lines through Gb3-mediated interactions. Similarly, SaroL-1 binding induced a dose-dependent cytotoxic effect resulting in the detachment of H1299 cells from the cell culture dish. Despite the fact that already sub-micromolar concentrations effectively killed 50% of cells the cytotoxic effect is markedly inhibited by pre-incubation of SaroL-1 with the αGal-containing competitor PNPG, which once more confirms a glycan-dependent mode of binding and action.

The crystal structures of SaroL-1 in complex with GalNAc and Gb3 trisaccharide provided the atomic basis for its specificity. They confirmed the presence of an aerolysin domain with a structure similar to LSL and aerolysin. We could propose a model of the entire pore using ε-toxin from *C. perfringens* as a template. The ability of SaroL-1 to form a pore was experimentally validated through hemolysis and penetration of labelled dextran into giant unilamellar vesicles. The oligomerization of Sarol-1 on Gb3-containing membranes was confirmed through cryogenic transmission electron microscopy. It could also be demonstrated that SaroL-1 is cytotoxic and induces cell detachment. The lectin-binding step is necessary for the pore-formation since the pre-incubation with a high affinity galactose derivative diminishes or abolishes hemolysis, GUVs penetration, and cytotoxicity.

We could demonstrate here that SaroL-1 displays carbohydrate-dependent cytotoxic activity on cancer cells that overexpress Gb3. We could also demonstrate that SaroL-1 is not active on Gb3- depleted cancer cells. As recently reviewed^67^, Gb3 is expressed in a limited number of human organs (lung, kidney, lymph node and CNS), so SaroL-1 might not be specific solely towards cancer cells, limiting possible applications in targeted treatments. Nonetheless, the Gb3-targeting verotoxin proved to be selective in the elimination of human tumor xenografts in mice^68,69^. Moreover, several tumors, such as secondary ovarian metastases and tumors refractory to chemotherapy that display very high level of Gb3^70^, could be targets of interest. Additional studies are necessary to establish the specificity of SaroL-1 towards cancer and non-cancerous tissues, according to Gb3 distribution.

Recently, PFTs gained interest for their application as biotechnological sensors and delivery systems. Aerolysin is a well-characterized nanopore, wild-type and mutated forms demonstrated promising results as nanopores for direct sensing of nucleotide acids and proteins^71,72^, or even charged polysaccharides^73^. The applications for early diagnosis, such as detecting circulating cancer cells, are promising^74^. Aerolysin was demonstrated to identify most of the twenty proteinogenic amino acids^75^. Together with the characterization of new aerolysins from biodiversity, these findings pave the way for nanopore-based sequencing of proteins. With an increasing number of possible applications for PFTs, SaroL-1 is a suitable candidate for further exploration in the field of nanopore technology and cancer diagnosis and treatment.

## Methods

### Construction of the TrefLec database

Trefoil structures in UniLectin3D are grouped together in a class if they share 20% of sequence similarity with any other structure of the same class. Within each trefoil class, the lectin lobes have been manually selected based on PDB 3D structures and aligned with the Muscle software^76^. When a protein has several associated 3D structures, only one of them is selected.

Twelve distinct trefoil lectin classes were defined. The sequences defining the trefoil lobes in each class are extracted individually and aligned together. This resulted in collecting 12 multiple alignments of lobe conserved regions. These were used as input to HMMER-hmmbuild^77^, using default parameters and symfrac (the minimum residue fraction necessary to set a position as consensus in the alignment) set at 0.8, to generate twelve characteristic profiles for each class. HMMER is an established tool that produces Hidden Markov Models (HMM)^78^, that is, a signature/profile for a group of similar protein sequences. Using these 12 trefoil lectin class profiles, potential lectin sequences were predicted in UniProtKB (UniProt April 2019)^79^ and NCBI-nr (non-redundant July 2019)^80^. TrefLec was updated in 2021 and, at the time of writing, includes more β-trefoil candidates. The two large protein sequence datasets (millions of sequences) were screened with the 12 HMM profiles using HMMER-hmmsearch, with default parameters and a *p*-value below 0.01^77^.

Further filtering was applied to discard highly similar (>98%) proteins of the same species (only one instance was kept), as well as identified domains <10 amino acids. The HMMER tool generates a statistical score for each predicted lectin. Since each sequence is compared to 12 profiles, a prediction is assigned 12 scores, which, by nature of the score definition (bit score), are not comparable across the 12 classes. That is why, to use a single cut-off value in TrefLec, a normalised prediction/similarity score (between 0 and 1) was defined. It reflects the similarity between the predicted lobe sequence and the matched profile lobe consensus sequence. In this way, for each sequence, the 12 prediction results can be ranked, and the top one is selected following published procedure^81^.

### Datamining for β-trefoil lectins with aerolysin domains

The “Search by Field” option of the TrefLec database (https://www.unilectin.eu/trefoil/search) was used with the option “aerolysin” in the PFAM window or the option “proaerolysin” in the CATH window.

### Gene design and cloning

The original gene sequence of the uncharacterized protein from *Salpingoeca rosetta* (strain ATCC 50818/BSB-021) (NW_004754913.1) was obtained from the UniLectin3D database. The gene *sarol-1* was ordered from Eurofins Genomics (Ebersberg, Germany) after codon optimization for the expression in the bacteria *Escherichia coli*. The restriction enzyme sites of NdeI and XhoI were added at 5' and 3' ends, respectively. The synthesized gene was delivered in plasmid pEX-A128-SaroL-1. This plasmid and the pET-TEV vector^82^ were digested with the NdeI and XhoI restriction enzymes to ligate *sarol-1* in pET-TEV to fuse a 6-His Tag cleavable with TEV protease at the *N*-terminus of SaroL-1. After transformation by heat shock in *E. coli* DH5α strain, a colony screening was performed, and the positive plasmid was amplified and controlled by sequencing.

### Protein expression

*E. coli* BL21(DE3) cells were transformed by heat shock with pET-TEV-SaroL-1 plasmid prior preculture in Luria Broth (LB) media with 25 μg/mL kanamycin at 37 °C under agitation at 180 rpm overnight. The next day, 10 mL of preculture was used to inoculate 1 L LB medium with 25 μg/mL kanamycin at 37 °C and agitation at 180 rpm. When the culture reached OD_600nm_ of 0.6–0.8, the protein expression was induced by adding 0.1 mM isopropyl β-D-thiogalactoside (IPTG), and the cells were cultured at 16 °C for 20 h. The cells were harvested by centrifugation at 14,000 × *g* for 20 min at 4 °C and the cell paste was resuspended in 20 mM Tris/HCl pH 7.5, 100 mM NaCl, and lysed by a pressure cell disruptor (Constant Cell Disruption System) with a pressure of 1.9 kBar. The lysate was centrifuged at 24,000 × *g* for 30 min at 4 °C and filtered on a 0.45 µm syringe filter prior to loading on an affinity column.

### Seleno-methionine protein expression

Substitution of the methionine of SaroL-1 by selenomethionine was performed according to protocol^83^. *E. coli* BL21(DE3) pET TEV-SaroL-1 were precultured in 5 mL LB Broth media with 25 μg/mL kanamycin at 37 °C with agitation at 180 rpm until OD_600nm_ reached 0.6. The pre-culture was centrifuged (10 min, 3000 × *g*, 4 °C) and washed twice by 1 × M9 medium. 1 × M9 media is composed of salts (Na2HPO4-2H2O, KH2PO4, NaCl, NH4Cl) enriched with 0.4% glucose, 1 mM MgSO_4_, 0.3 mM CaCl_2_, 1 μg thiamine, and sterile water. The cells were cultured in 50 mL 1 × M9 minimal salts media and 25 μg/mL kanamycin at 37 °C with agitation at 180 rpm overnight. Then, the preculture was transferred into 1 L 1 × M9 medium with the addition of 25 μg/mL kanamycin at 37 °C and agitation at 180 rpm. When OD_600nm_ reached 0.6–0.8, the mixture of the amino acids (Lys, Phe, Thr, Ile, Leu, Val, SeMet) was added and incubated for 15 min at 37 °C with agitation at 180 rpm prior to induction and treatment of the cells as described above for the wild-type protein. The cell pellet was stored at −20 °C prior to purification.

### Protein purification

The sample was loaded on 1 mL HisTrap column (Cytiva) pre-equilibrated with 20 mM Tris/HCl pH 7.5, 100 mM NaCl (Buffer A). The column was washed with Buffer A to remove all contaminants and unbound proteins. The SaroL-1 was eluted by Buffer A in steps during which the concentration of imidazole was increased from 50 mM to 500 mM. The fractions were analyzed by 12% SDS PAGE and those containing SaroL-1 were collected and deprived of imidazole using a PD10 desalting column (Cytiva). The sample was concentrated by Pall centrifugal device with MWCO 10 kDa prior to loading onto Enrich 70 column (BioRad) previously equilibrated with Buffer A for further purification. After analysis by SDS-PAGE, the pure protein fractions were pooled, concentrated and stored at 4 °C.

### Isothermal Titration Calorimetry (ITC)

ITC experiments were performed with MicroCaliTC200 (Malvern Panalytical). Experiments were carried out at 25 °C ± 0.1 °C. SaroL-1 and ligands samples were prepared in Buffer A. The ITC cell contained SaroL-1 in a concentration range from 0.05 mM to 0.16 mM. The syringe contained the ligand solutions in a concentration from 10 to 50 mM. 2 μL of ligands solutions were injected into the sample cell at intervals of 120 s while stirring at 750 rpm. Integrated heat effects were analysed by nonlinear regression using one site binding model (MicroCal PEAQ-ITC Analysis software). The experimental data were fitted to a theoretical curve, which gave the dissociation constant (Kd) and the enthalpy of binding (∆H).

### Protein labelling

SaroL-1 was dissolved at 1 mg/mL in Dulbecco’s phosphate-buffered saline (PBS) and stored at 4 °C prior to usages. For fluorescent labelling, Cy5 (GE Healthcare) mono-reactive NHS ester and NHS-ester conjugated Atto488 (Thermo Fisher) were used. Fluorescent dyes were dissolved at a final concentration of 1 mg/mL in water-free DMSO (Carl RothGmbH & Co), aliquoted, and stored at −20 °C before usage according to the manufacturer´s protocol. For the labelling reaction, 100 µL of lectin (1 mg/mL) was supplemented with 10 µL of a 1 M NaHCO_3_ (pH 9) solution. Hereby, the molar ratio between dye and lectin was 2:1. The labelling mixture was incubated at 4 °C for 90 min, and uncoupled dyes were separated using Zeba Spin desalting columns (7 kDa MWCO, 0.5 mL, Thermo Fischer). Labelled SaroL-1 was stored at 4 °C, protected from light.

### Composition and preparation of giant unilamellar vesicles (GUVs)

GUVs were composed of 1,2-dioleoyl-sn-glycero-3-phosphocholine (DOPC), cholesterol (both AvantiPolar Lipids, United States), Atto 647 N 1,2-dioleoyl-sn-glycero-3-phosphoethanolamine (DOPE; Sigma-Aldrich), and one of the following glycolipids at a molar ratio of 64.7:30:0.3:5. The glycolipids are globotriaosylceramide (Gb3, Matreya), FSL-Gb3 (Function-Spacer-Lipid with globotriaosyl saccharide), and corresponding FSL-isoGb3 (Function-Spacer-Lipid with iso-globotriaosyl saccharide) synthetized as previously described^84,85^, FSL-A(tri) (Function-Spacer-Lipid with blood group A trisaccharide; Sigma-Aldrich), FSL-B(tri) (Function-Spacer-Lipid with blood group B trisaccharide; Sigma-Aldrich) or lactosylceramide (LC, Sigma-Aldrich).

GUVs were prepared by the electroformation method as earlier described^86^. Briefly, lipids dissolved in chloroform of a total concentration of 0.5 mg/mL were spread on indium tin oxid-covered (ITO) glass slides and dried in a vacuum for at least 1 h or overnight. Two ITO slides were assembled to create a chamber filled with sucrose solution adapted to the osmolarity of the imaging buffer of choice, either HBSS (live-cell imaging) or PBS (GUVs only imaging). Then, an alternating electrical field with a field strength of 1 V/mm was implemented for 2.5 h at RT. Later we observed the GUVs in chambers manually built as described^86^.

### Imaging of SaroL-1 binding to GUVs

Samples of GUVs and SaroL-1 were imaged using a confocal fluorescence microscope (Nikon Eclipse Ti-E inverted microscope equipped with Nikon A1R confocal laser scanning system, 60x oil immersion objective, NA = 1.49 and four laser lines: 405 nm, 488 nm, 561 nm, and 640 nm). Image acquisition and processing were made using the software NIS-Elements (version 4.5, Nikon) and open-source Fiji software (https://imagej.net/software/fiji/).

### Dextran influx into GUVs

Dextran Alexa Fluor 488 (Dextran-AF488) with a MW of 3 kDa (Thermo Fischer) or 70 kDa FITC-dextran (Sigma-Aldrich) was added to the observation chamber (0.02 mg/mL) in PBS buffer together with 200 nM SaroL-1. Then wt Gb3-containing GUVs (40 µL) were added for imaging and monitoring of pore-formation. Dextran-AF488 was present in the GUVs’ surrounding solution. For PNPG treatment, 200 nM of SaroL-1 was pre-incubated with 10 mM PNPG dissolved in DMSO for 15 min at RT in the observation chamber. Directly after, dextran-AF488 (0.02 mg/mL) and 40 µL wt Gb3-containing GUVs were added to the observation chamber and monitored using confocal microscopy.

### Hemolytic assay (HA) and inhibition of hemolytic activity (IHA)

Both HA and IHA assays were performed in U-shaped 96-well microtiter plates. In both cases, a control negative experiment was performed with Buffer A added to erythrocyte solution (2% final concentration), and the resulting value was subtracted from all data.

SaroL-1 with an initial concentration of 27 μM was diluted by serial 2-fold dilution in Buffer A. Rabbit erythrocytes were purchased from Atlantis France and used without further washing. The erythrocytes were diluted to a 4% solution in 150 mM NaCl. Protein solution and rabbit erythrocytes were mixed together in a 1:1 ratio and incubated for 1 h at 37 °C. After the samples were centrifuged, 2600 × *g* for 15 min at RT and the absorbance of the supernatant was measured at 540 nm by a Tecan reader.

IHA was performed in U-shaped 96-well microtiter plates. Various carbohydrates (PNPG, GalNAc, lactose, melibiose) with the initial concentration of 100 mM were serially 2-fold diluted in Buffer A. SaroL-1 with a final concentration in the well 217 nM was added into ligand solution in 1:1 ratio and incubated for 1 h at RT. 4% rabbit erythrocytes were added to the mixture in a 1:1 ratio and incubated for 1–2 h at 37 °C. The samples were centrifuged, 2600 × *g* for 15 min at RT, and the absorbance of the supernatant was measured at 540 nm by a Tecan reader.

### Optical microscopy

SaroL-1 (110 nM and 217 nM) was added into a 4% solution of rabbit erythrocytes and observed after incubation for 5 and 30 min, respectively, at RT. The samples were observed Zeiss Axioplan 2 microscope with 40x magnification. 4% solution of rabbit erythrocytes was used as a negative control.

### Cell culture

The human lung epithelial cell line H1299 (American Type Culture Collection, CRL-5803) was cultured in a complete medium, consisting of Roswell Park Memorial Institute (RPMI) medium supplemented with 10% fetal calf serum (FCS) and 2 mM L-glutamine at 37 °C and 5% CO_2_. Cells were stimulated with different concentrations of SaroL-1 in a complete medium for indicated time points.

### Flow cytometry analysis

H1299 cells were detached with 1.5 mM EDTA in PBS −/−, and 1 × 10^5^ cells were counted and transferred to a U-bottom 96-well plate (Sarstedt AG & Co.). To determine the binding of SaroL-1 to surface receptors, cells were incubated with fluorescently labelled protein for 30 min at 4 °C and protected from light, in comparison with PBS-treated cells as a negative control. To deplete cells of glucosylceramide-based glycosphingolipids, they were cultivated 72 h in the presence of 2 μM DL-threo-1-phenyl-2-palmitoylamino-3-morpholino-1-propanol (PPMP) (Sigma-Aldrich) to inhibit the synthesis of glucosylceramide-based GSLs^87^ and incubated with fluorescent SaroL-1. Subsequently, cells were centrifuged at 1600 × *g* for 3 min at 4 °C to remove unbound lectin. The samples were then washed two times with ice-cold FACS buffer (PBS (−/−) supplemented with 3% FCS (*v/v*)). After the last washing step, the cells were re-suspended with FACS buffer and transferred to FACS tubes (Kisker Biotech GmbH & Co) on ice and protected from light. The fluorescence intensity of treated cells was measured with FACS Gallios from Beckman Coulter. The samples were further analyzed using FlowJo V.10.5.3.

### Fluorescence imaging of SaroL-1 binding and cellular uptake by confocal microscopy

Between 4 and 5 × 10^4^ cells were seeded on 12-mm glass coverslips in a 4-well plate and cultured for 1 day before the experiment. Cells were incubated with SaroL-1-Cy5 for indicated time points at 37 °C, in comparison with PBS-treated cells as a negative control. Subsequently, cells were fixed with 4% paraformaldehyde for 15 min at RT and quenched with 50 mM ammonium chloride for 5 min. The membrane was permeabilised, and cells were blocked by 0.2% Saponin in 3% BSA in PBS (*w/v*) for 30 min. The cell nuclei were counterstained with DAPI (5 × 10^−9^ g/L), and the samples were mounted on coverslips using Mowiol (containing the anti-bleaching reagent DABCO). Samples were imaged utilising a laser scanning confocal microscope system from Nikon (Eclipse Ti-E, A1R), equipped with a 60x oil immersion objective and a numerical aperture (*NA*) of 1.49. The images were further analyzed using NIS-Element Confocal 4.20 from Nikon and ImageJ 1.52a from Laboratory for Optical and Computational Instrumentation. A minimum of three biological replicates with ≥20 cells per condition were analyzed.

### Cell proliferation (MTT) assay

To investigate the cytotoxic effect of SaroL-1 on human cells, H1299 cells were treated with increasing concentrations of SaroL-1 for 24 h in a standard MTT assay. For the depletion of Gb3 from the plasma membrane, H1299 cells were treated with 2 µM PPMP for 72 h before the experiment. 3.5 × 10^4^ cells per well were transferred to a 96-well plate with a U-bottom. The cells were centrifuged at 1600 × *g* for 3 min at RT. The cell pellet was re-suspended in 100 μL of variously concentrated protein solutions (3, 14, 27, 68, 135, 270, 680, 1360 nM) and transferred to a 96-well flat-bottomed plate. The cells were incubated for 24 h at 37 °C. Subsequently, 10 μL of MTT labelling solution (MTT Cell Proliferation Kit, Roche) was added to each well, and the cells were incubated for 4 h at 37 °C. Then, 100 μL of the solubilisation reagent was added to each well, and the plate was incubated at 37 °C overnight. The next day, the absorbance of the samples was measured at 550 nm using a BioTek microplate reader. To assess SaroL-1 cytotoxicity in the presence of the soluble sugar PNPG, 3.5 × 10^4^ cells per well were transferred to a 96-well plate with a U-bottom. Variously concentrated protein solutions (3, 14, 27, 68, 135, 270, 680, 1360 nM) were preincubated with 10 mM PNPG dissolved in complete medium, for 15 min at RT. The cells were centrifuged at 1600 × *g* for 3 min at RT. Next, the cell pellet was re-suspended in 100 μL protein solution and transferred to a 96-well flat-bottomed plate. The cells were incubated for 24 h at 37 °C, and the MTT assay was performed as described above. The data was further analyzed using GraphPad Prism software.

### Lactate dehydrogenase (LDH) assay

This assay was used to monitor rapid changes in cell viability following the formation of pores induced by SaroL-1. Prior to running the assay, kit components were assembled according to CyQuant^TM^ LDH Cytotoxicity assay kit (Invitrogen—Thermo Fisher Scientific) manufacturer´s instructions. 1 × 10^4^ cells per well were plated in 100 µL in triplicates in a 96-well plate with a flat bottom and allowed to adhere overnight. The next day, 271 nM or 1358 nM of SaroL-1 were added to the samples, and controls including wells in triplicate for Spontaneous LDH activity and Maximum LDH activity. The plate was incubated at 37 °C for indicated time points. Spontaneous LDH activity control wells were incubated with 10 µL of sterile, ultrapure water, while Maximum LDH activity controls included 10 µL of 10X Lysis Buffer. At the end of incubation, 50 µl of each sample medium were transferred to a new 96-well flat-bottom plate, to which 50 µL of Reaction mixture were added to each well. After 30 min\ of incubation at room temperature and in absence of light, 50 µL of Stop solution were added to each well and the absorbance of samples was recorded at 490 nm and 680 nm using a BioTek microplate reader. To determine LDH activity, the reference absorbance at 690 nm (background) was subtracted from the 490 nm absorbance values before calculation of % cytotoxicity. % cytotoxicity was calculated according to the following formula = [SaroL-1-treated LDH activity − spontaneous LDH activity]/[maximum LDH activity − spontaneous LDH activity] × 100.

### Cell detachment assay

To determine the role of SaroL-1 in cell detachment, 3 × 10^5^ cells per well were counted and allowed to adhere overnight in a 6-well plate. The next day, cells were incubated for 2, 4, or 8 h with two different concentrations of SaroL-1 (270 nM and 1.36 µM) diluted in a complete medium compared to a negative control. As a positive control, cells were incubated with trypsin-EDTA for 10 min at 37 °C, to induce 100% cell detachment. The number of cells in suspension at the indicated time points was quantified by analyzing the supernatant with the CytoSmart Corning cell counter. Cells were resuspended with Trypan Blue solution (Sigma-Aldrich) in a ratio 1:1 for the quantification of cellular death upon detachment.

### Crystallization and structure determination of SaroL-1

The protein dissolved in Buffer A to 9 mg/mL was co-crystallized with 10 mM GalNAc and 10 mM Gb3 trisaccharide after incubation for at least 2 h at RT. Crystallization screening was performed using the vapor diffusion method with hanging drops of 2 μL drops containing a 1/1 (*v/v*) mix of protein and reservoir solution at 19 °C. Crystal clusters were obtained after several days from a solution containing 0.1 M buffer system 4, pH 6.5 (MOPSO/Bis-Tris, Molecular Dimensions), 100 mM AA Morpheus I, 25 or 35% PEG SMEAR MEDIUM for native SaroL-1 GalNAc and Gb3 complex crystals, respectively and 0.1 M buffer system 4 pH 6.5, 100 mM AA (Arg, Thr, Lys, His), 45% precipitant mix 6 Morpheus II (Molecular Dimensions) for SeMet protein. Single crystals were directly mounted in a cryoloop and flash-freezed in liquid nitrogen. Diffraction data were collected at 100 K at the Soleil Synchrotron (Paris, France) on Proxima-2 for SeMet and GalNAc structures and Proxima-1 for the Gb3 complex using a DECTRIS EIGER X 9M and 16 M detector, respectively. The data were processed using XDS^88^ and XDSME (GitHub). All further computing was performed using the CCP4i suite^89^. Data quality statistics are summarized in Supplementary Table 3. The SeMet SaroL-1 served as the initial structure where the phases were solved experimentally, and the model was built using Crank2 on MAD data, including peak and inflection point^90^. The structures of SaroL-1/GalNAc and SaroL-1/Gb3 were solved by molecular replacement using PHASER and the coordinates of SeMet protein as search model^91^. The structures were refined with restrained maximum likelihood refinement using REFMAC 5.8, and local NCS restrains^92^ iterated with manual rebuilding in Coot^93^. Five percent of the observations were set aside for cross-validation analysis, and hydrogen atoms were added in their riding positions and used for geometry and structure-factor calculations. Incorporation of the ligand was performed after inspection of the ARP/WARP 2Fo-DFc weighted maps. The library for hexanetriol was constructed with Sketcher and Libcheck in CCP4i. Water molecules were inspected manually. The model was validated with the wwPDB Validation server: http://wwpdb-validation.wwpdb.org. The coordinates were deposited in the Protein Data Bank under code 7QE3 for SeMet SaroL-1, 7QE4 for the native SaroL-1 in complex with GalNAc and 7R55 for the native SaroL-1 in complex with Gb3.

### Cryogenic transmission electron microscopy (cryo TEM)

Gb3-containing large unilamellar vesicles (LUVs) were used for cryo TEM observations for their smaller size (100 nm) compared to that of GUVs. 1.5 μL of SaroL-1 (32 μM) were added to 50 μL of LUV suspension (lipid concentration of 0.5 mg/mL) and incubated at RT for 3 h. A droplet (ca. 3 μL) of the incubated suspension was applied on a glow-discharged lacey carbon-coated grid. The grid was then blotted and plunge-frozen in liquid ethane using an automatic plunge freezer (EM-GP, Leica Microsystems, Germany). Cryogenic transmission electron microscopy was performed using a JEM-2100Plus (JEOL Ltd., Japan) operated at an accelerating voltage at 200 kV and an Elsa cryo-transfer holder (Gatan Inc., USA). All images were recorded by a Gatan Rio 16 camera (Gatan Inc., USA) under low-dose conditions using SerialEM software^94^ and analyzed using the Fiji program^95^.

### Statistics and reproducibility

All data in graphs are presented as mean ± standard deviation (SD) and were calculated from the results of independent experiments. Statistical testing was performed with GraphPad Prism software and Microsoft Excel using data of ≥3 biological replicates. Statistical differences in independent, identical samples were determined with a two-tailed, unpaired *t*-test. Tests with a *p*-value ≤ 0.05 were considered statistically significant and marked with an asterisk (*). *p*-values ≤ 0.01 are shown as two asterisks (**), ≤0.001 are summarized with three asterisks (***) and ≤0.0001 are indicated as four asterisks (****).

### Reporting summary

Further information on research design is available in the Nature Research Reporting Summary linked to this article.

## Supplementary information

Supplemental Material

Description of Additional Supplementary Files

Supplementary Data 1

Reporting Summary

validation report 7QE3

validation report 7QE4

validation report 7R55

## Data Availability

Coordinates and structure factors generated in this study have been deposited in the Protein Data Bank under accession codes 7QE3 for the structure of seleno-Met SaroL-1, and 7QE4 and 7R55 for the structure of the complexes of SaroL-1 with GalNAc and Gb3 trisaccharide, respectively. Uncropped protein gel is available as Supplementary Fig. 13.
