# Supplementary material for: The choanoflagellate pore-forming lectin SaroL-1 punches holes in cancer cells by targeting the tumor-related glycosphingolipid Gb3
Source: Commun Biol. 2022 Sep 12;5:954. doi: 10.1038/s42003-022-03869-w (PMC9468336; doi:10.1038/s42003-022-03869-w)
Supplement: Supplementary file 1 — Supplemental Material [file 42003_2022_3869_MOESM1_ESM.pdf]

The choanoflagellate pore-forming lectin SaroL-1 punches holes in cancer cells by targeting a tumor-related glycosphingolipid Gb3

Supplementary Information

Supplementary Table 1*: Selection of protein particular architectures composed of β-trefoil domain(s) and other functional domains, identified using Pfam motifs.*

Family	PFAM	Species	ProteinAC	PfamAC
Ricin-like trefoil	Melibiase 2	*Catenulispora acidiphila*	C7Q336	PF16499, PF17801
Ricin-like trefoil	Lipase GDSL 2	*Catellatospora citrea*	A0A419XYK	PF13472
Ricin-like trefoil	Arabinase	*Lentzea aerocolonigenes*	A0A0F0GI39	PF09206
Ricin-like trefoil	Glycosyltransferase GT2	*Trachymyrmex septentrionalis*	A0A151JT25	PF00535
Ricin-like trefoil	Glycosylhydrolase GH16	*Streptomyces sp* BK335	A0A4V2ULV4	PF00722
Ricin-like trefoil	CBM 6 (cellulose-binding domain), GSDH (Glucose / Sorbosone dehydrogenase), PKD 4 (Polycystic Kidney Disease)	*Saccharothrix sp* ALI22I	A0A1V2PXN8	PF03422, PF07995, PF18911
Coprinus trefoil	LSM RNA-binding proteins	*Thanatephorus cucumeris*	A0A0B7G0E7	PF01423
Earthworm trefoil	Lipase GDSL 2	*Cellulomonas persica*	A0A510UZ07	PF13472
Cys-rich man-receptor trefoil	Fibronectin type II, C-type lectin-like	*Homo sapiens*, *Rattus norvegicus*	Q9UBG0, Q4TU93	PF00040, PF05473
Fungi and Clostridium trefoil	Aerolysin	*Laetiporus sulphureus*	Q7Z8V1	PF01117

Supplementary Table 2: *ITC results of SaroL-1 binding to different ligands. The stoichiometry N was fixed to value 3 in all cases.*

**Ligand**	**Kd (mM)**	**Δ H (kcal/mol)**	**Δ G (kcal/mol)**	**-T Δ S (kcal/mol)**
αGal1-4Gal	0.39 ± 0.02	-6.36 ± 0.20	-4.65	1.71
PNPG	1.01 ± 0.02	-7.36 ± 0.11	-4.09	3.28
αGal1-6Glc	1.20 ± 0.06	-12.7 ± 0.39	-3.98	8.76
αGal1-3Gal	1.38 ± 0.28	-7.46 ± 0.11	-3.90	3.56
GalαOMe	2.17 ± 0.27	-5.67 ± 0.46	-3.63	2.03
GalNAc	2.76 ± 0.01	-11.8 ± 0.31	-3.49	8.35
βGal1-4Glc	6.66 ± 1.4	-4.98 ± 1.20	-2.97	2.01

Supplementary Table 3: *Direct hydrogen bonds observed in native SaroL-1 structures between α-GalNAc and Gb3 trisaccharide with amino acids of the three binding sites. Distances (Å) are averaged between the two chains (standard deviation < 0.1 Å).*

		Site α	***Site β***	***Site γ***
αGalNAc	O3	R26.NH2<br>2.85	R103.NH2<br>2.85	R150.NH2<br>2.85
	O4	R26.NH1<br>2.9	R103.NH1<br>2.95	R150.NH1<br>2.90
	N2	H32.ND1<br>2.60	H83.ND1<br>2.75	H129.ND1<br>2.75
	O6	G35.N<br>2.90<br>N140.OD1<br>2.50	G86.N<br>2.85<br>D43.OD1<br>2.65	G132.N<br>2.75<br>E92.OE2<br>2.55
Gb3	O3 (αGal)		S100.OG<br>2.6<br>R103.NH1<br>2.85	R150.NH2<br>2.8
	O4 (αGal)		R103.NH2<br>3<br>H83.ND1<br>2.7	R150.NH2<br>2.9<br>H129.ND1<br>2.6
	O6 (αGal)		D43.OD1<br>2.75<br>G86.N<br>2.9	E92.OE2<br>2.5<br>G132.N<br>2.75
	O2 (βGal)		D43.OD2<br>2.5	E92.OE2<br>2.65
	O5 (βGal)		H98.NE2<br>3.35	Y146.OH<br>3.5
	O1 (αGlc)			K91.NZ<br>3.2
	O2 (αGlc)		N42.ND2<br>3.35	K91.NZ<br>2.8
	O3 (αGlc)		H98.NE2<br>3.1	Y146.OH<br>3
	O4 (αGlc)		H98.NE2<br>3.2	Y146.OH<br>3.45
	O6 (αGlc)		D43.OD2<br>3.3	

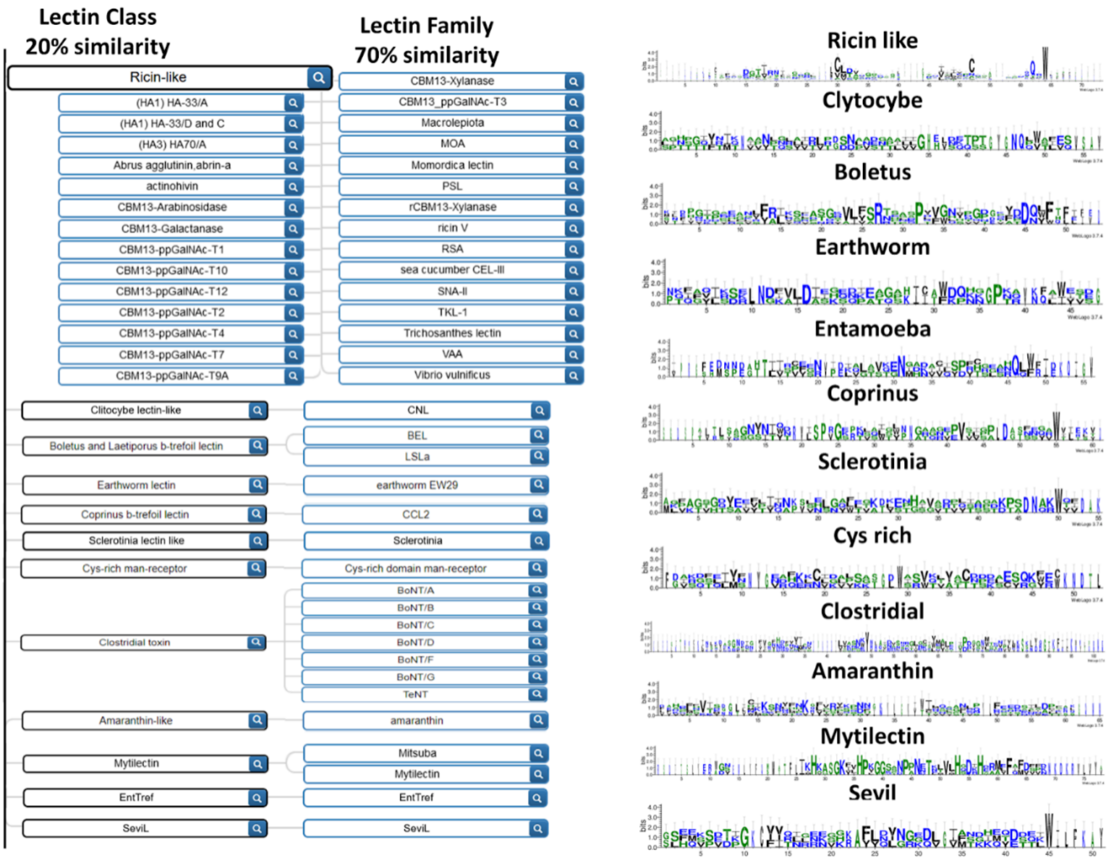

Supplementary Figure 1: *The classification of β-trefoil lectins and their binding motif signature generated by WebLOGO (Crooks et al. 2004, doi: 10.1101/gr.849004.)*

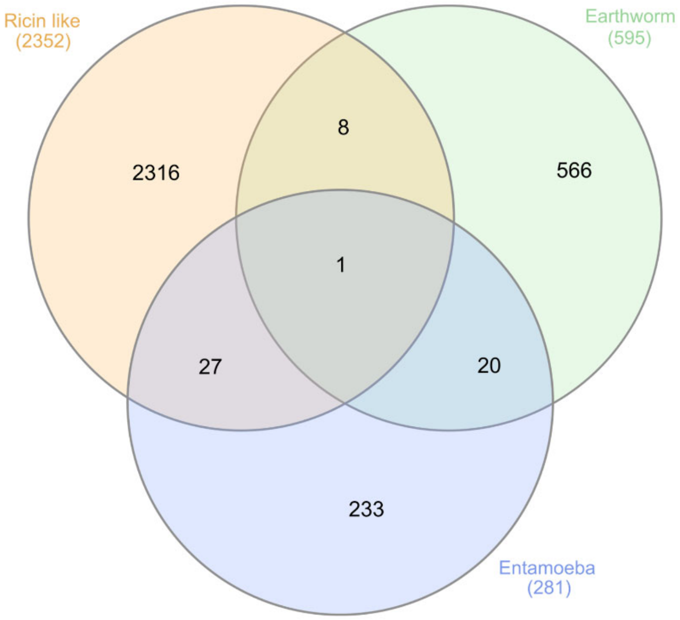

Supplementary Figure 2: *Venn diagram representing the overlap between the β-trefoil classes in predicted lectins, for a score > 0.25. Graphics by InteractiVenn (Heberle et al. 2015, doi: 10.1186/s12859-015-0611-3).*

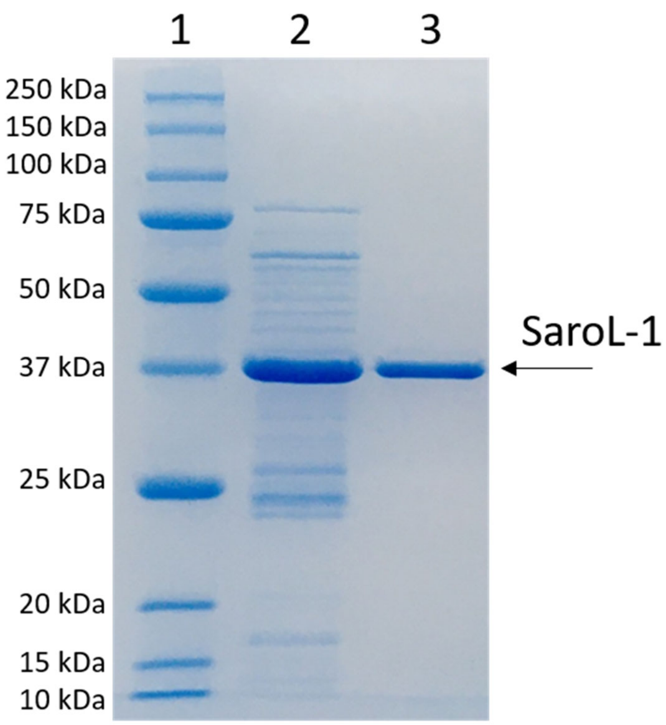

Supplementary Figure 3: *Analysis of SaroL-1 by denaturating SDS page gel electrophoresis. 12 % SDS gel, row 1 – protein marker, row 2 – elution of metal affinity chromatography, row 3 – elution of size exclusion chromatography. The molecular weight of SaroL-1 was estimated as 36.86 ± 0.76 kDa.*

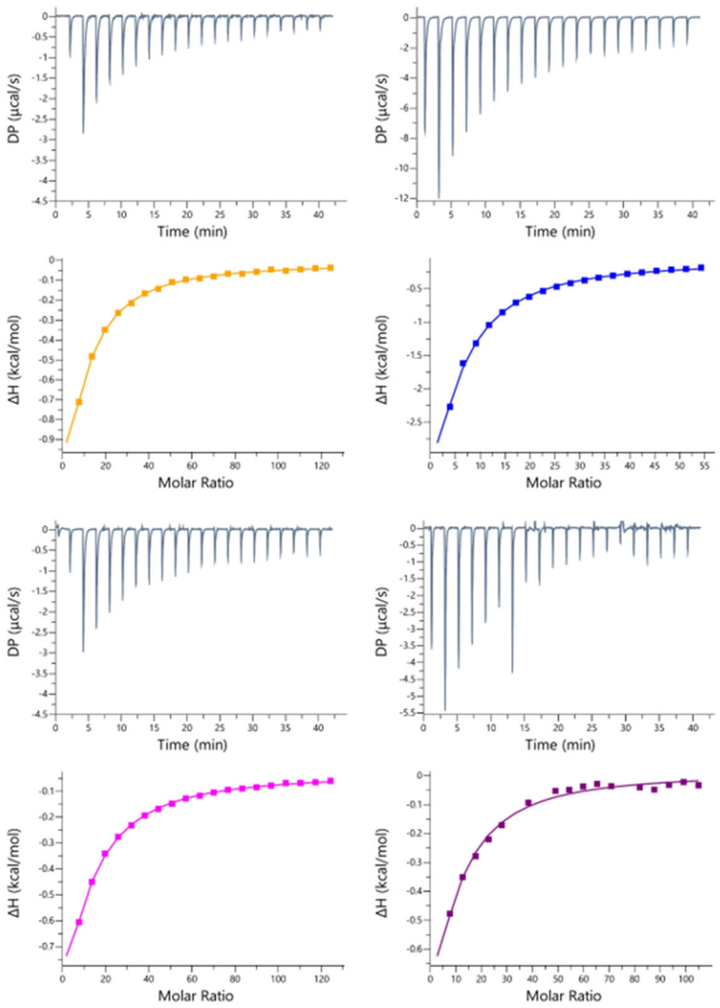

Supplementary Figure 4: *ITC data of SaroL-1 with different carbohydrate, the thermograms (top) and integrated peaks (bottom). The ITC cell contained SaroL-1 in the concentration range of 0.050-0.116 mM. The syringe contained ligands, such as PNPG (orange), αGal1-6Glc (melibiose) (blue), αGal1-3Gal (magenta) and GalαOMe (purple) in the concentration range of 10-50 mM.*

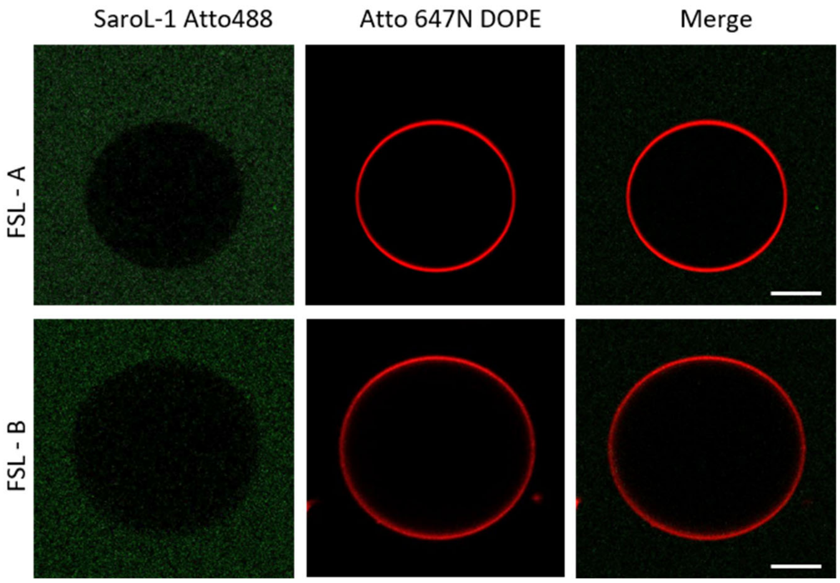

Supplementary Figure 5: *Binding assay of 200 nM of SaroL-1 (green) and GUVs (red). GUVs are functionalised with FSL-A and FSL-B (function-spacer-lipid with either blood group A or blood group B trisaccharide). Scale bars represent 10 µm.*

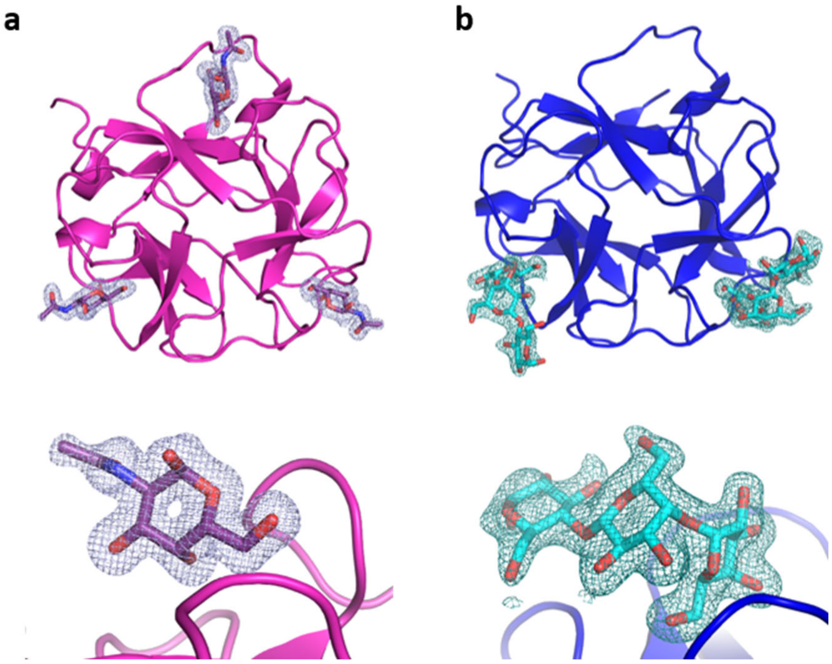

Supplementary Figure 6: *Electron density map of ligands. a) β-trefoil domain of SaroL-1 in complex with αGalNAc, zoom on β-site, b) β-trefoil domain of SaroL-1 in complex with Gb3, zoom on β-site.*

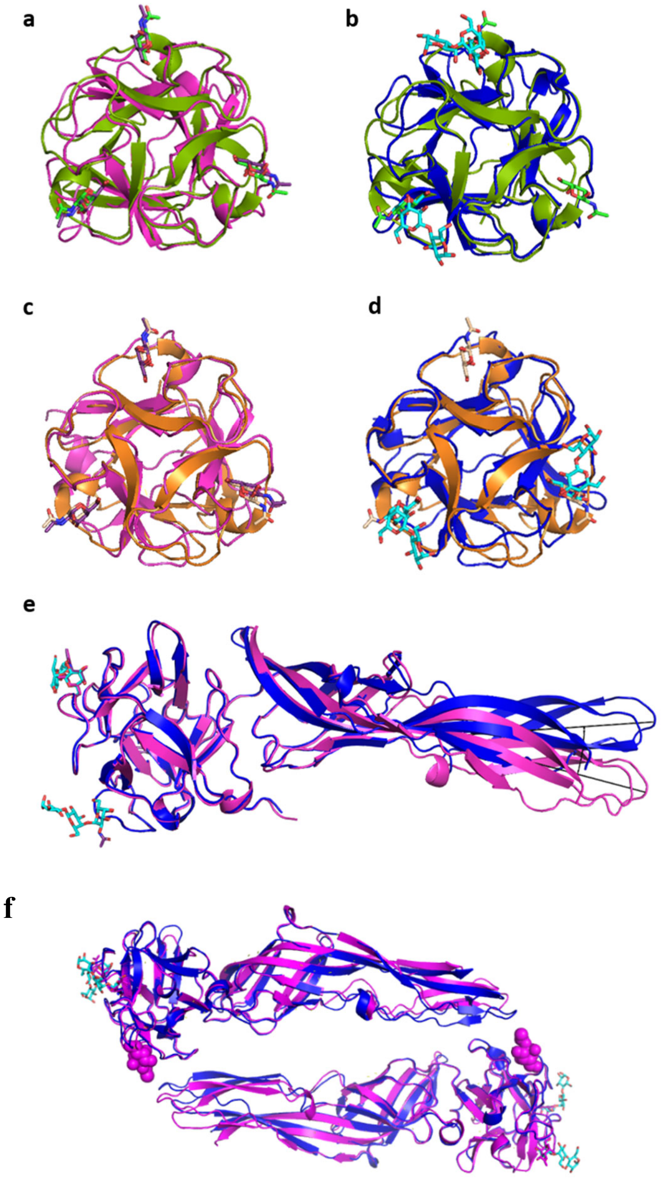

Supplementary Figure 7: *Structures superimposition. a) β-trefoil domain of SaroL-1 in light magenta in complex with GalNAc (violet) (7QE4) with Mytilec (olive green) in complex with GalNAc (green) (3WMV). b) β-trefoil domain of SaroL-1 in blue in complex with Gb3 (cyan) (7R55) with Mytilec (olive green) in complex with GalNAc (green) (3WMV). c) β-trefoil domain of SaroL-1 in light magenta in complex with GalNAc (violet) (7QE4) with Mitsuba (orange) in complex with GalNAc (pale orange) (5XG5). d) β-trefoil domain of SaroL-1 in blue in complex with Gb3 (cyan) (7R55) with Mitsuba (orange) in complex with GalNAc (pale orange) (5XG5). e) Shift of pore-forming domain between chain A of SaroL-1/GalNAc (light magenta) with SaroL-1/Gb3 (blue). The difference angle is measured as SaroL-1/Gb3/D194 - SaroL-1/GalNAc/R287 - SaroL-1/GalNAc/D194 = 17.6°.). f) Same comparison displaying the dimer contacts in the crystal packing with contacting GalNAc in site α represented by spheres.*

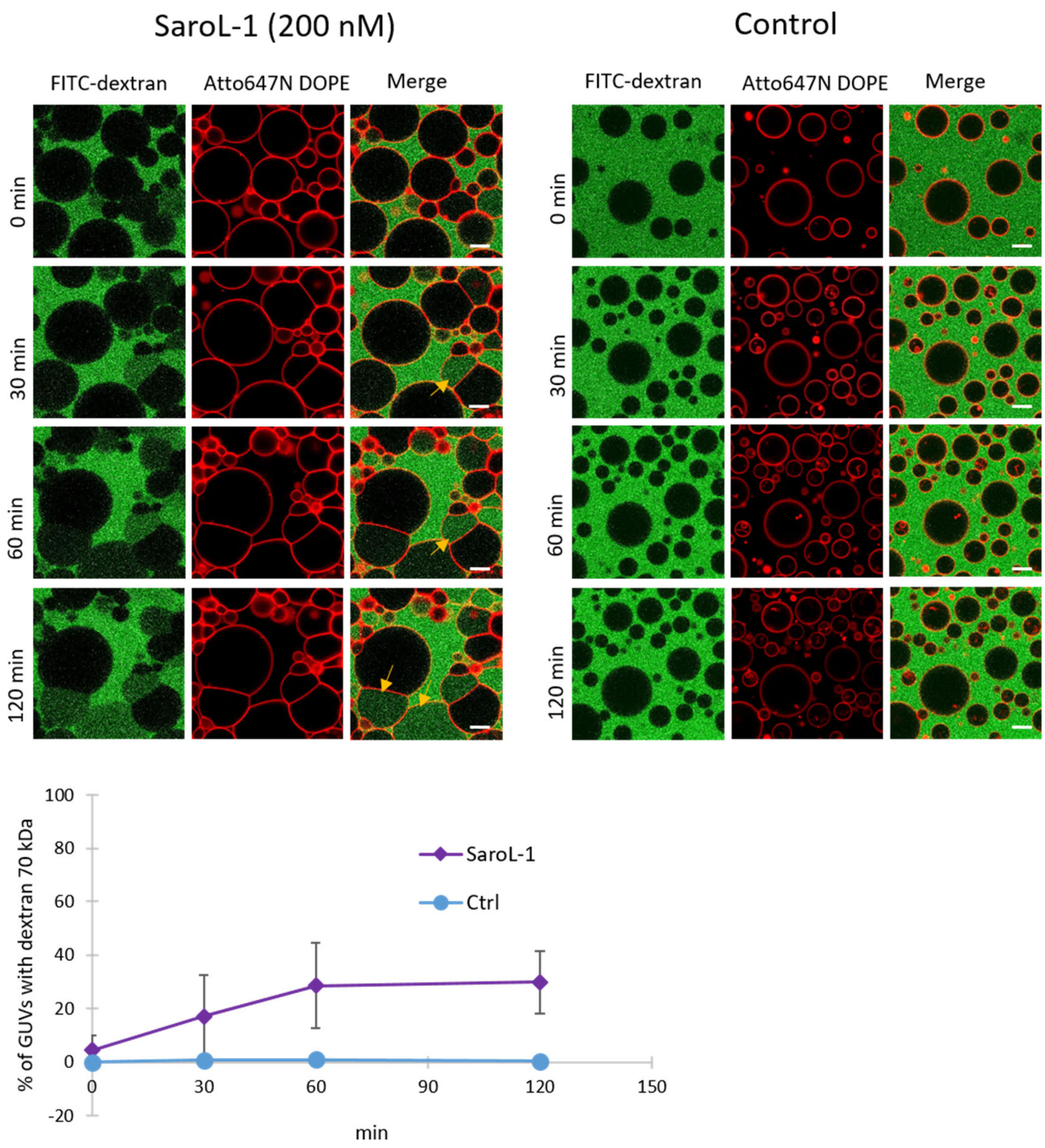

Supplementary Figure 8: Pore-forming activity of SaroL-1. *SaroL-1 (unlabeled, 200 nM) triggers the influx of 70 kDa FITC-dextran (green) into wt Gb3-containing GUVs (red) via its pore-forming activity. In the control group without SaroL-1, there was no visible influx of dextran-AF488 detected. Yellow arrows indicate events of FITC-dextran influx to wt Gb3-GUVs. The GUVs were composed of DOPC, cholesterol, wt Gb3, and membrane dye to the molar ratio of 64.7:30:5:0.3, respectively. The scale bars represent 10 µm. Mean values ± SD are shown. Data represent three independent experiments, n=3. The molecular weight of fluorescently labelled dextran is 70 kDa. The total amount of control GUVs was at 0 min – 200 GUVs, 30 min – 279 GUVs, 60min – 294 GUVs and 120 min – 302 GUVs. For SaroL-1 experiment with wt Gb3-GUVs, total amount of GUVs was 0 min – 199 GUVs, 30 min – 211 GUVs, 60 min – 215 GUVs and 120 min – 227 GUVs. Data for the graphs are available in Supplementary Data 1.*

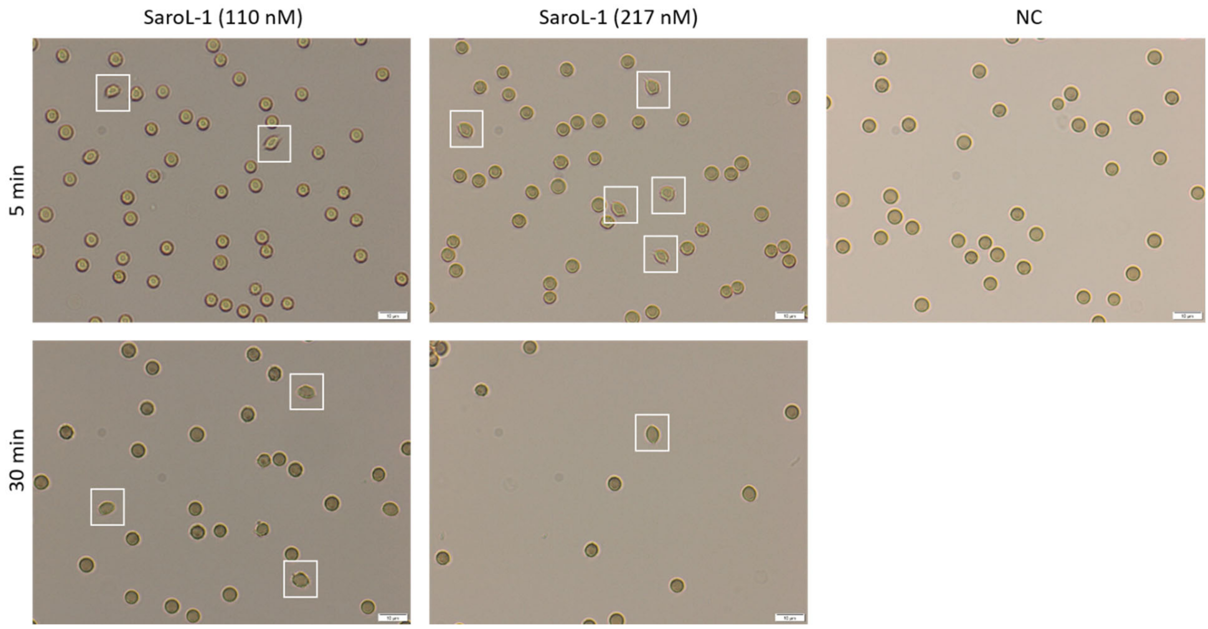

Supplementary Figure 9: *The almond-like shape of the rabbit erythrocytes is observed once incubated with SaroL-1 (110 nM and 217 nM). After 5 and 30 min of incubation, the red blood cells underwent morphological changes (white rectangles) due to SaroL-1 binding. A solution of untreated rabbit erythrocytes (NC) functioned as negative control showing the natural shape of red blood cells.*

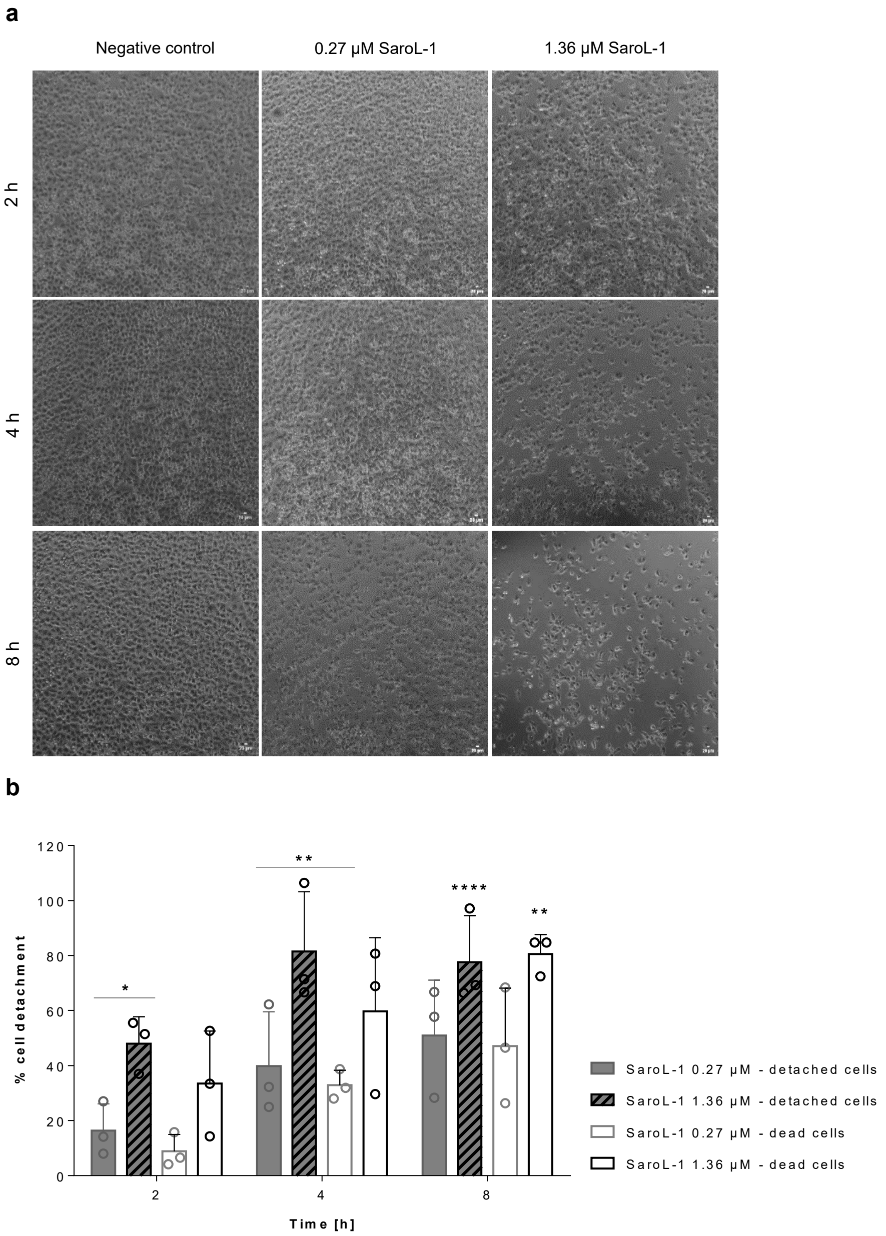

Supplementary Figure 10: *SaroL-1 binding to H1299 cells induce cell detachment. a) Adherent H1299 cells were treated with SaroL-1 (271 nM and 1.36 µM) for 8 hours and observed by light microscopy. SaroL-1 caused a dose-dependent rounding and detachment of cells in comparison to treatment with PBS. At the highest concentration, SaroL-1's induced detachment of cells is visible at early time point (2 h), whereas 271 nM SaroL-1 promoted cell detachment at 8 h. Scale bars represent 20 µm. b) Quantification of cell detachment and death induced by SaroL-1 treatment for different time points (2, 4, 8 hours). The increase in cell detachment was quantified by analyzing the supernatant with CytoSmart Corning cell counter (means ± SD, n = 3). Viability of detached cells upon SaroL-1 treatment was assessed with Trypan blue staining. Significant difference compared to the negative control for each time point,* $*p \leq 0.05$, $**p \leq 0.01$, $***p \leq 0.001$, $****p \leq 0.0001$.

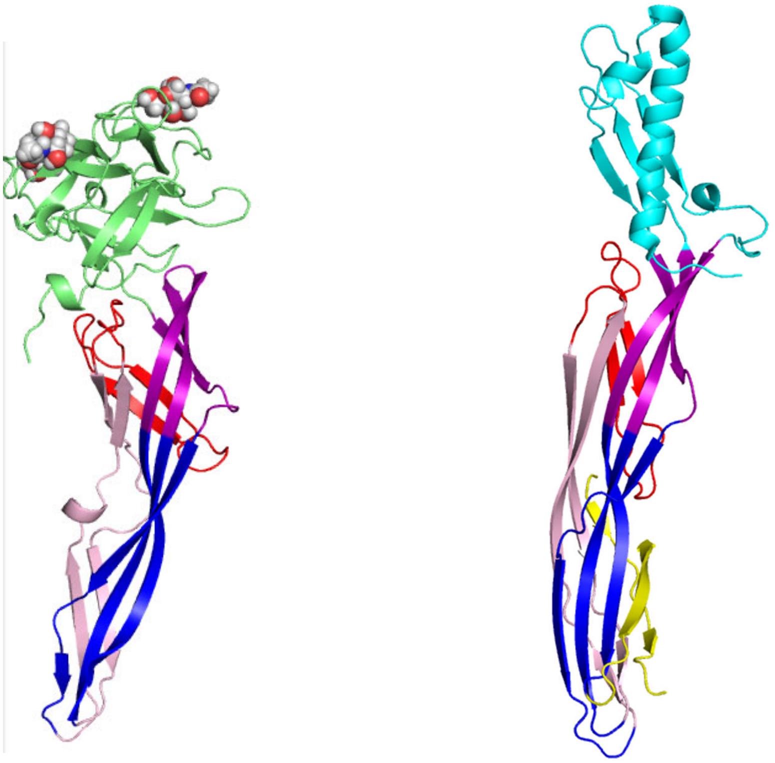

Supplementary Figure 11: *Structural similarities between SaroL-1 (left) and ε-toxin of C. perfringens (PDB 1UYJ, right), used for aligning the sequences for further model building of the pore-forming domain.*

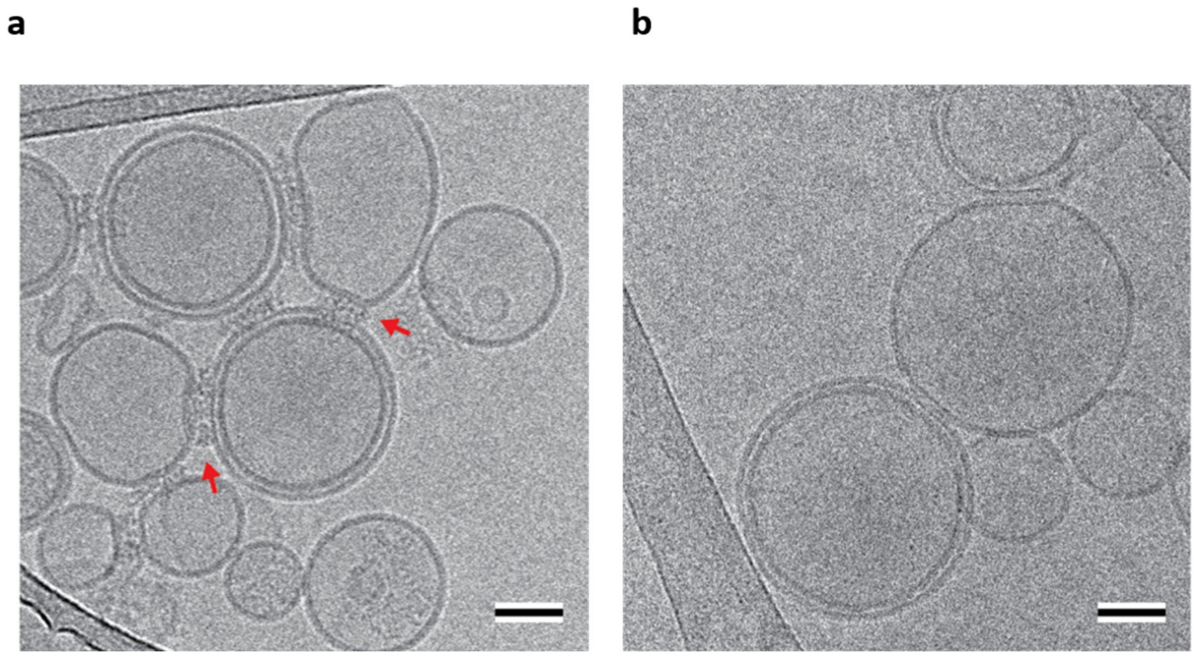

Supplementary Figure 12: *Cryo TEM images of Gb3 decorated LUVs. a) SaroL-1 clusters crosslink liposome surfaces (indicated by red arrows). b) negative control – solution of LUVs without addition of SaroL-1. Scale bars are 50 nm.*

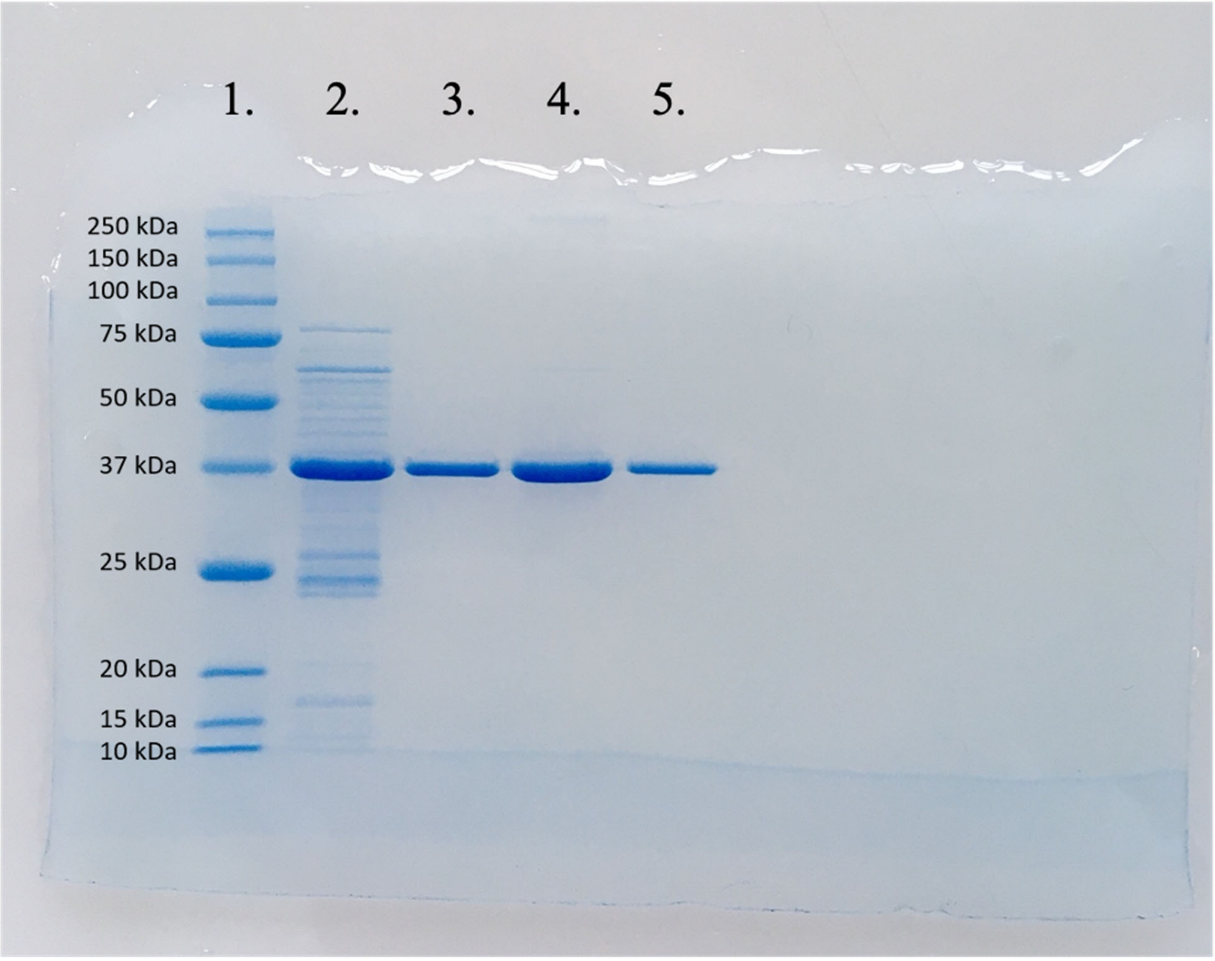

Supplementary Figure 13: *Uncropped version of Supplementary Figure 3. Analysis of SaroL-1 by denaturing SDS page gel electrophoresis. 12 % SDS gel, uncropped gel corresponding Supplementary Figure 3. Row 1 – protein marker, row 2 – elution of metal affinity chromatography, row 3,4,5 – elution of size exclusion chromatography. The molecular weight of SaroL-1 was estimated as 36.86 ± 0.76 kDa.*
