# Supplementary material for: The choanoflagellate pore-forming lectin SaroL-1 punches holes in cancer cells by targeting the tumor-related glycosphingolipid Gb3
Source: Commun Biol. 2022 Sep 12;5:954. doi: 10.1038/s42003-022-03869-w (PMC9468336; doi:10.1038/s42003-022-03869-w)
Supplement: Supplementary file 2 — Description of Additional Supplementary Files [file 42003_2022_3869_MOESM2_ESM.pdf]

Description of Additional Supplementary Files

File name: Supplementary Data 1
Description: The source data behind the graphs of Figure 6 and Supplemental figure 8.
