# Supplementary material for: The choanoflagellate pore-forming lectin SaroL-1 punches holes in cancer cells by targeting the tumor-related glycosphingolipid Gb3
Source: Commun Biol. 2022 Sep 12;5:954. doi: 10.1038/s42003-022-03869-w (PMC9468336; doi:10.1038/s42003-022-03869-w)
Supplement: Supplementary file 4 — Reporting Summary [file 42003_2022_3869_MOESM4_ESM.pdf]

nature portfolio

Corresponding author(s): Dr. Anne Imberty

Last updated by author(s): Jul 27, 2022

Reporting Summary

Nature Portfolio wishes to improve the reproducibility of the work that we publish. This form provides structure for consistency and transparency in reporting. For further information on Nature Portfolio policies, see our Editorial Policies and the Editorial Policy Checklist.

Statistics

For all statistical analyses, confirm that the following items are present in the figure legend, table legend, main text, or Methods section.

n/a	Confirmed	
☐	☒	The exact sample size (n) for each experimental group/condition, given as a discrete number and unit of measurement
☐	☒	A statement on whether measurements were taken from distinct samples or whether the same sample was measured repeatedly
☐	☒	The statistical test(s) used AND whether they are one- or two-sided<br>*Only common tests should be described solely by name; describe more complex techniques in the Methods section.*
☒	☐	A description of all covariates tested
☒	☐	A description of any assumptions or corrections, such as tests of normality and adjustment for multiple comparisons
☐	☒	A full description of the statistical parameters including central tendency (e.g. means) or other basic estimates (e.g. regression coefficient) AND variation (e.g. standard deviation) or associated estimates of uncertainty (e.g. confidence intervals)
☒	☐	For null hypothesis testing, the test statistic (e.g. F, t, r) with confidence intervals, effect sizes, degrees of freedom and P value noted<br>*Give P values as exact values whenever suitable.*
☒	☐	For Bayesian analysis, information on the choice of priors and Markov chain Monte Carlo settings
☒	☐	For hierarchical and complex designs, identification of the appropriate level for tests and full reporting of outcomes
☒	☐	Estimates of effect sizes (e.g. Cohen's d, Pearson's r), indicating how they were calculated

Our web collection on statistics for biologists contains articles on many of the points above.

Software and code

Policy information about availability of computer code

Data collection	NIS-Elements (version 4.5, Nikon)<br>FACS Gallios (Beckman Coulter Inc.)<br>MXCube
Data analysis	XDS, CCP4, REFMAC, ARP/WARP, GraphPad Prism, NIS-Element Confocal 4.20, ImageJ 1.52a, FlowJo V.10.5.3, MicroCal-PEAQ ITC

For manuscripts utilizing custom algorithms or software that are central to the research but not yet described in published literature, software must be made available to editors and reviewers. We strongly encourage code deposition in a community repository (e.g. GitHub). See the Nature Portfolio guidelines for submitting code & software for further information.

Data

Policy information about availability of data

All manuscripts must include a data availability statement. This statement should provide the following information, where applicable:

- Accession codes, unique identifiers, or web links for publicly available datasets
- A description of any restrictions on data availability
- For clinical datasets or third party data, please ensure that the statement adheres to our policy

Coordinates and structure factors have been deposited in the Protein Data Bank under the accession codes: 7QE3, 7QE4 and 7R55.

March 2021

Field-specific reporting

Please select the one below that is the best fit for your research. If you are not sure, read the appropriate sections before making your selection.

☒ Life sciences ☐ Behavioural & social sciences ☐ Ecological, evolutionary & environmental sciences

For a reference copy of the document with all sections, see nature.com/documents/nr-reporting-summary-flat.pdf

Life sciences study design

All studies must disclose on these points even when the disclosure is negative.

Sample size	Between 178 and 466 GUVs analyzed per time point and per condition while the number of GUVs fluctuated over the time of experiment.<br>Between 40.000 and 50.000 cells were seeded and analyzed for confocal imaging.<br>100.000 cells were seeded for each sample in flow cytometry, 10.000 cells were analyzed per condition.
Data exclusions	No data were excluded in these studies
Replication	Affinity values from IMT data result from duplicated measurements, cytotoxicity assay, cell detachment assay, flow cytometry, confocal imaging
Randomization	Randomization was not applicabe in these studies
Blinding	Blinding was not relevant in these studies

Reporting for specific materials, systems and methods

We require information from authors about some types of materials, experimental systems and methods used in many studies. Here, indicate whether each material, system or method listed is relevant to your study. If you are not sure if a list item applies to your research, read the appropriate section before selecting a response.

Materials & experimental systems

n/a	Involved in the study	
☒	☐	Antibodies
☐	☒	Eukaryotic cell lines
☒	☐	Palaeontology and archaeology
☒	☐	Animals and other organisms
☒	☐	Human research participants
☒	☐	Clinical data
☒	☐	Dual use research of concern

Methods

n/a	Involved in the study	
☒	☐	ChIP-seq
☐	☒	Flow cytometry
☒	☐	MRI-based neuroimaging

Eukaryotic cell lines

Policy information about cell lines

Cell line source(s)	American Type Culture Collection, Human lung epithelial cell line H1299
Authentication	CRL-5803
Mycoplasma contamination	None
Commonly misidentified lines (See ICLAC register)	None

Flow Cytometry

Plots

Confirm that:

☒ The axis labels state the marker and fluorochrome used (e.g. CD4-FITC).

☒ The axis scales are clearly visible. Include numbers along axes only for bottom left plot of group (a 'group' is an analysis of identical markers).

☐ All plots are contour plots with outliers or pseudocolor plots.

☒ A numerical value for number of cells or percentage (with statistics) is provided.

Methodology

Sample preparation	H1299 cells were detached with 1.5 mM EDTA in PBS -/-, and 100.000 cells were counted and transferred to a U-bottom 96 well plate. Cells were incubated with fluorescently labelled protein for 30 min at 4°C and protected from light, in comparison with PBS-treated cells as a negative control. Subsequently, cells were centrifuged at 1600 ×g for 3 min at 4°C to remove unbound lectin. The samples were then washed two times with ice-cold FACS buffer. After the last washing step, the cells were re-suspended with FACS buffer and transferred to FACS tubes on ice and protected from light. The fluorescence intensity of treated cells was measured immediately with FACS Gallios from Beckman Coulter.
Instrument	FACS Gallios (Beckman Coulter Inc.)
Software	FlowJo V.10.5.3
Cell population abundance	10.000 cells
Gating strategy	Gating for viable cells is applied to all samples. Gating is performed on FSC-A/SSC-A plots.

☐ Tick this box to confirm that a figure exemplifying the gating strategy is provided in the Supplementary Information.
