# Supplementary material for: The choanoflagellate pore-forming lectin SaroL-1 punches holes in cancer cells by targeting the tumor-related glycosphingolipid Gb3
Source: Commun Biol. 2022 Sep 12;5:954. doi: 10.1038/s42003-022-03869-w (PMC9468336; doi:10.1038/s42003-022-03869-w)
Supplement: Supplementary file 5 — validation report 7QE3 [file 42003_2022_3869_MOESM5_ESM.pdf]

Full wwPDB X-ray Structure Validation Report (i)

Feb 4, 2022 – 09:42 pm GMT

PDB ID	:	7QE3
Title	:	Se-M variant of B-trefoil lectin from Salpingoeca rosetta in complex with GalNAc
Deposited on	:	2021-12-01
Resolution	:	2.20 Å(reported)

This wwPDB validation report is for manuscript review

This is a Full wwPDB X-ray Structure Validation Report.

This report is produced by the wwPDB biocuration pipeline after annotation of the structure.

We welcome your comments at *validation@mail.wwpdb.org*
A user guide is available at
https://www.wwpdb.org/validation/2017/XrayValidationReportHelp
with specific help available everywhere you see the (i) symbol.

The following versions of software and data (see references (i)) were used in the production of this report:

MolProbity	:	4.02b-467
Mogul	:	1.8.4, CSD as541be (2020)
Xtriage (Phenix)	:	1.13
EDS	:	2.26
buster-report	:	1.1.7 (2018)
Percentile statistics	:	20191225.v01 (using entries in the PDB archive December 25th 2019)
Refmac	:	5.8.0267
CCP4	:	7.1.010 (Gargrove)
Ideal geometry (proteins)	:	Engh & Huber (2001)
Ideal geometry (DNA, RNA)	:	Parkinson et al. (1996)
Validation Pipeline (wwPDB-VP)	:	2.26

For Manuscript Review

1 Overall quality at a glance (i)

The following experimental techniques were used to determine the structure:
X-RAY DIFFRACTION

The reported resolution of this entry is 2.20 Å.

Percentile scores (ranging between 0-100) for global validation metrics of the entry are shown in the following graphic. The table shows the number of entries on which the scores are based.

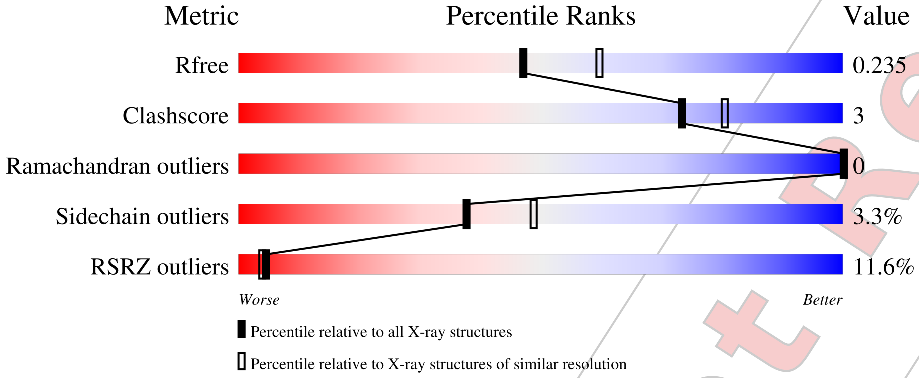

Metric	Whole archive (#Entries)	Similar resolution (#Entries, resolution range(Å))
R_{free}	130704	4898 (2.20-2.20)
Clashscore	141614	5594 (2.20-2.20)
Ramachandran outliers	138981	5503 (2.20-2.20)
Sidechain outliers	138945	5504 (2.20-2.20)
RSRZ outliers	127900	4800 (2.20-2.20)

The table below summarises the geometric issues observed across the polymeric chains and their fit to the electron density. The red, orange, yellow and green segments of the lower bar indicate the fraction of residues that contain outliers for >=3, 2, 1 and 0 types of geometric quality criteria respectively. A grey segment represents the fraction of residues that are not modelled. The numeric value for each fraction is indicated below the corresponding segment, with a dot representing fractions <=5% The upper red bar (where present) indicates the fraction of residues that have poor fit to the electron density. The numeric value is given above the bar.

Mol	Chain	Length	Quality of chain
1	AAA	350	11% / 83% 9% • 7%
1	BBB	350	10% / 83% 9% 8%

For Manuscript Review

2 Entry composition (i)

There are 7 unique types of molecules in this entry. The entry contains 5574 atoms, of which 0 are hydrogens and 0 are deuteriums.

In the tables below, the ZeroOcc column contains the number of atoms modelled with zero occupancy, the AltConf column contains the number of residues with at least one atom in alternate conformation and the Trace column contains the number of residues modelled with at most 2 atoms.

- Molecule 1 is a protein called Sarol-1.

Mol	Chain	Residues	Atoms						ZeroOcc	AltConf	Trace
1	AAA	325	Total<br>2521	C<br>1584	N<br>447	O<br>482	S<br>3	Se<br>5	0	2	0
1	BBB	322	Total<br>2511	C<br>1577	N<br>443	O<br>484	S<br>3	Se<br>4	0	4	0

There are 42 discrepancies between the modelled and reference sequences:

Chain	Residue	Modelled	Actual	Comment	Reference
AAA	-20	MSE	-	initiating methionine	UNP F2UID9
AAA	-19	GLY	-	expression tag	UNP F2UID9
AAA	-18	SER	-	expression tag	UNP F2UID9
AAA	-17	SER	-	expression tag	UNP F2UID9
AAA	-16	HIS	-	expression tag	UNP F2UID9
AAA	-15	HIS	-	expression tag	UNP F2UID9
AAA	-14	HIS	-	expression tag	UNP F2UID9
AAA	-13	HIS	-	expression tag	UNP F2UID9
AAA	-12	HIS	-	expression tag	UNP F2UID9
AAA	-11	HIS	-	expression tag	UNP F2UID9
AAA	-10	SER	-	expression tag	UNP F2UID9
AAA	-9	SER	-	expression tag	UNP F2UID9
AAA	-8	GLY	-	expression tag	UNP F2UID9
AAA	-7	GLU	-	expression tag	UNP F2UID9
AAA	-6	ASN	-	expression tag	UNP F2UID9
AAA	-5	LEU	-	expression tag	UNP F2UID9
AAA	-4	TYR	-	expression tag	UNP F2UID9
AAA	-3	PHE	-	expression tag	UNP F2UID9
AAA	-2	GLN	-	expression tag	UNP F2UID9
AAA	-1	GLY	-	expression tag	UNP F2UID9
AAA	0	HIS	-	expression tag	UNP F2UID9
BBB	-20	MSE	-	initiating methionine	UNP F2UID9
BBB	-19	GLY	-	expression tag	UNP F2UID9
BBB	-18	SER	-	expression tag	UNP F2UID9
BBB	-17	SER	-	expression tag	UNP F2UID9

Continued on next page...

For Manuscript Review

Continued from previous page...

Chain	Residue	Modelled	Actual	Comment	Reference
BBB	-16	HIS	-	expression tag	UNP F2UID9
BBB	-15	HIS	-	expression tag	UNP F2UID9
BBB	-14	HIS	-	expression tag	UNP F2UID9
BBB	-13	HIS	-	expression tag	UNP F2UID9
BBB	-12	HIS	-	expression tag	UNP F2UID9
BBB	-11	HIS	-	expression tag	UNP F2UID9
BBB	-10	SER	-	expression tag	UNP F2UID9
BBB	-9	SER	-	expression tag	UNP F2UID9
BBB	-8	GLY	-	expression tag	UNP F2UID9
BBB	-7	GLU	-	expression tag	UNP F2UID9
BBB	-6	ASN	-	expression tag	UNP F2UID9
BBB	-5	LEU	-	expression tag	UNP F2UID9
BBB	-4	TYR	-	expression tag	UNP F2UID9
BBB	-3	PHE	-	expression tag	UNP F2UID9
BBB	-2	GLN	-	expression tag	UNP F2UID9
BBB	-1	GLY	-	expression tag	UNP F2UID9
BBB	0	HIS	-	expression tag	UNP F2UID9

- Molecule 2 is 2-acetamido-2-deoxy-beta-D-galactopyranose (three-letter code: NGA) (formula: $C_8H_{15}NO_6$) (labeled as "Ligand of Interest" by depositor).

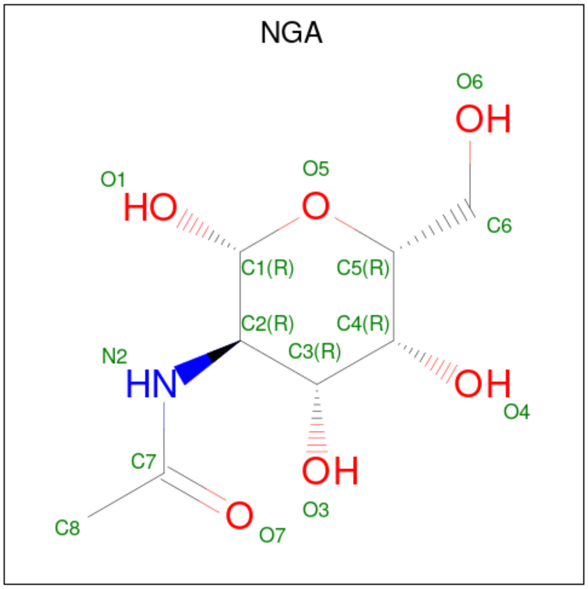

Mol	Chain	Residues	Atoms	ZeroOcc	AltConf
2	AAA	1	Total C N O<br>15 8 1 6	0	0

- Molecule 3 is 1,2-ETHANEDIOL (three-letter code: EDO) (formula: $C_2H_6O_2$).

For Manuscript Review

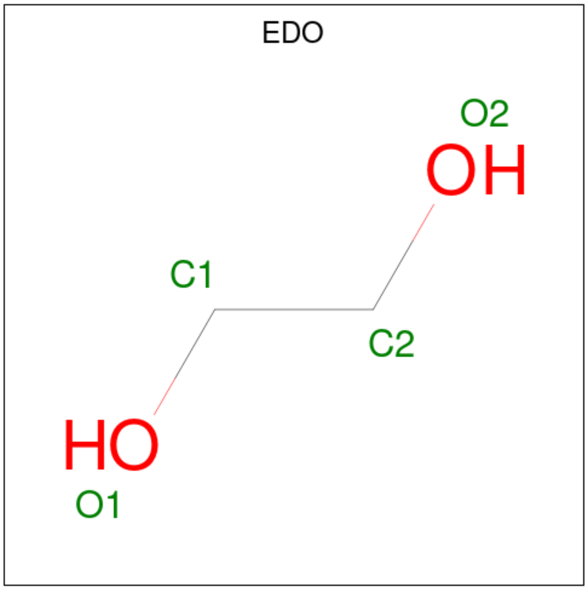

Mol	Chain	Residues	Atoms	ZeroOcc	AltConf
3	AAA	1	Total C O<br>4 2 2	0	0
3	BBB	1	Total C O<br>4 2 2	0	0
3	BBB	1	Total C O<br>4 2 2	0	0

- Molecule 4 is DI(HYDROXYETHYL)ETHER (three-letter code: PEG) (formula: $C_4H_{10}O_3$).

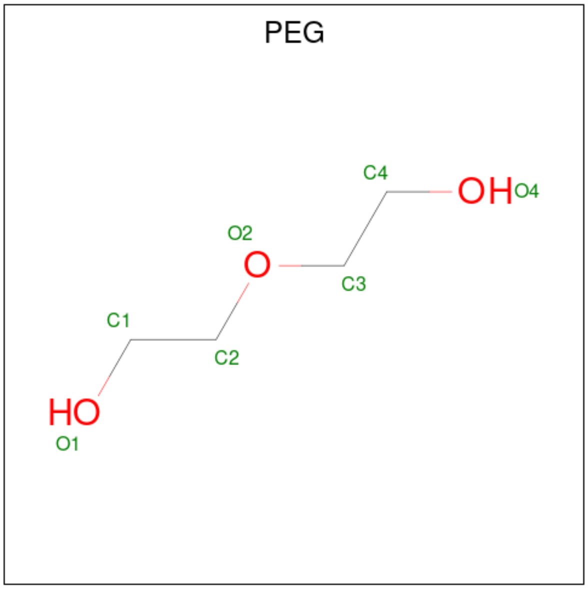

Mol	Chain	Residues	Atoms	ZeroOcc	AltConf
4	AAA	1	Total C O<br>7 4 3	0	0

For Manuscript Review

- Molecule 5 is (2S)-hexane-1,2,6-triol (three-letter code: 1JW) (formula: $C_6H_{14}O_3$).

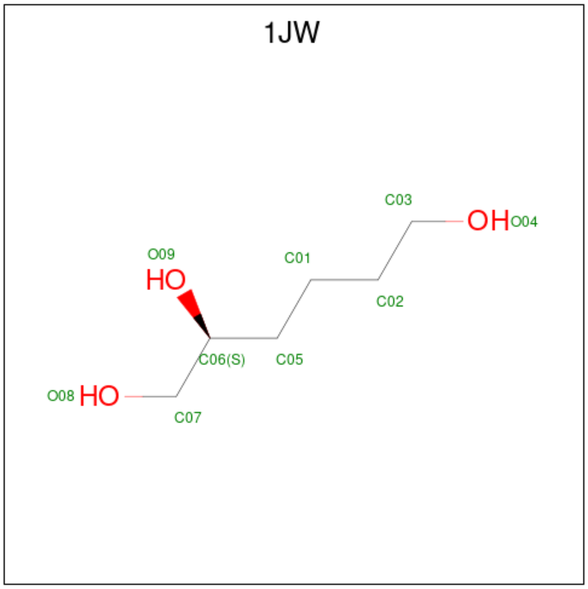

Mol	Chain	Residues	Atoms	ZeroOcc	AltConf
5	BBB	1	Total C O<br>9 6 3	0	0
5	BBB	1	Total C O<br>9 6 3	0	0
5	BBB	1	Total C O<br>9 6 3	0	0

- Molecule 6 is TRIETHYLENE GLYCOL (three-letter code: PGE) (formula: $C_6H_{14}O_4$).

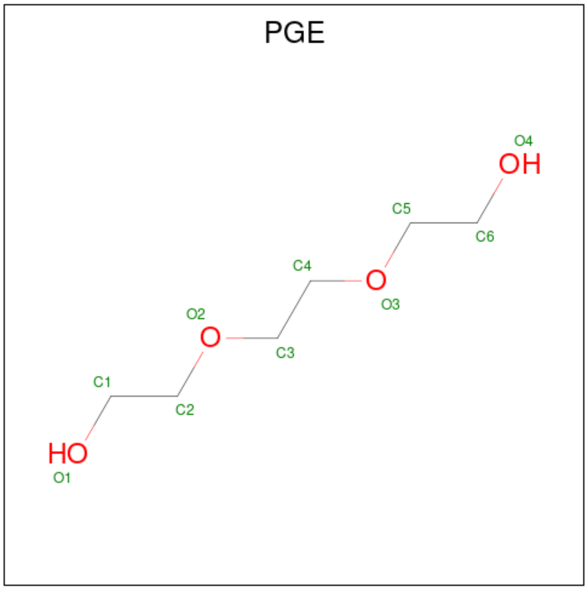

For Manuscript Review

Mol	Chain	Residues	Atoms	ZeroOcc	AltConf
6	BBB	1	Total C O<br>10 6 4	0	0

- Molecule 7 is water.

Mol	Chain	Residues	Atoms	ZeroOcc	AltConf
7	AAA	185	Total O<br>189 189	0	4
7	BBB	265	Total O<br>282 282	0	17

For Manuscript Review

3 Residue-property plots (i)

These plots are drawn for all protein, RNA, DNA and oligosaccharide chains in the entry. The first graphic for a chain summarises the proportions of the various outlier classes displayed in the second graphic. The second graphic shows the sequence view annotated by issues in geometry and electron density. Residues are color-coded according to the number of geometric quality criteria for which they contain at least one outlier: green = 0, yellow = 1, orange = 2 and red = 3 or more. A red dot above a residue indicates a poor fit to the electron density (RSRZ > 2). Stretches of 2 or more consecutive residues without any outlier are shown as a green connector. Residues present in the sample, but not in the model, are shown in grey.

- Molecule 1: Sarol-1

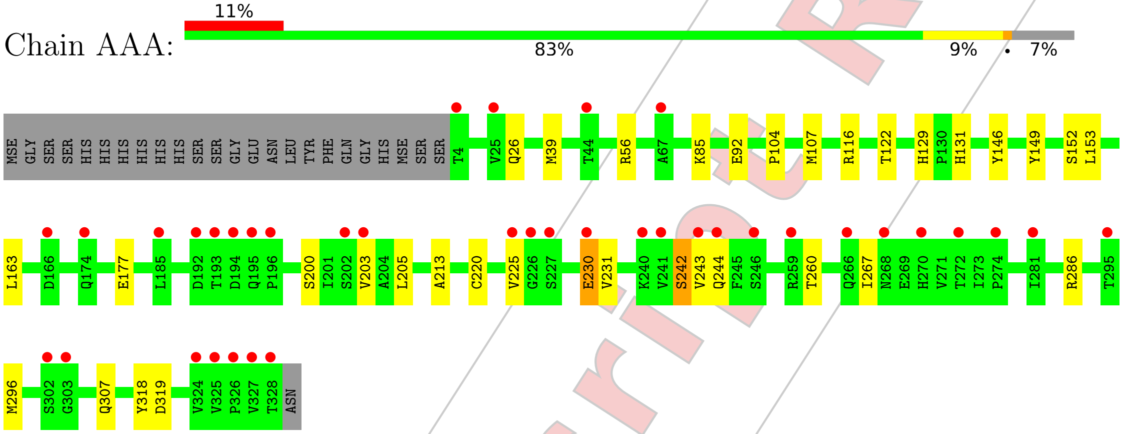

- Molecule 1: Sarol-1

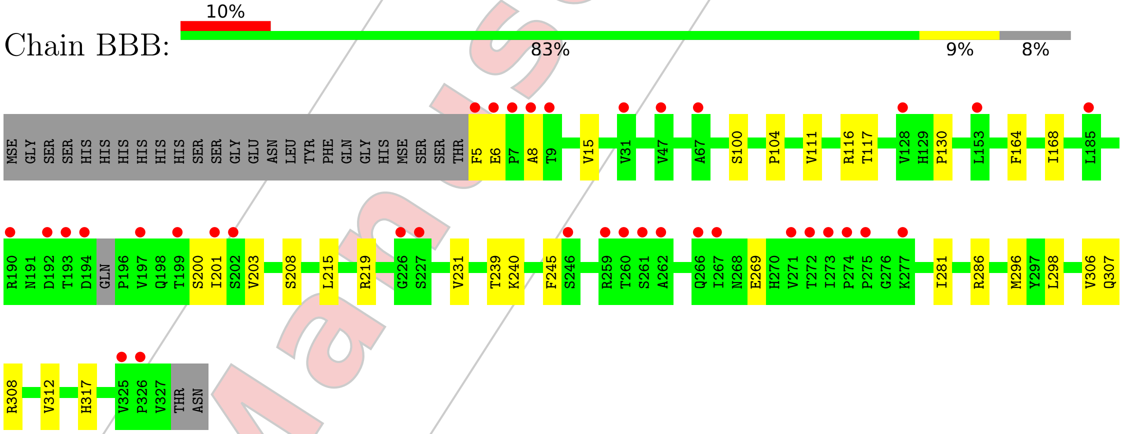

For Manuscript Review

4 Data and refinement statistics (i)

Property	Value	Source
Space group	P 1 21 1	Depositor
Cell constants<br>a, b, c, α, β, γ	44.99Å 58.58Å 166.42Å<br>90.00° 90.68° 90.00°	Depositor
Resolution (Å)	39.77 – 2.20<br>39.77 – 2.20	Depositor<br>EDS
% Data completeness<br>(in resolution range)	99.4 (39.77-2.20)<br>99.4 (39.77-2.20)	Depositor<br>EDS
R_{merge}	0.10	Depositor
R_{sym}	(Not available)	Depositor
$< I/\sigma(I) >$ [1]	2.48 (at 2.20Å)	Xtriage
Refinement program	REFMAC 5.8.0267	Depositor
R, R_{free}	0.182 , 0.231<br>0.191 , 0.235	Depositor<br>DCC
R_{free} test set	2095 reflections (4.75%)	wwPDB-VP
Wilson B-factor (Å^2)	28.3	Xtriage
Anisotropy	0.467	Xtriage
Bulk solvent k_{sol}(e/Å^3), B_{sol}(Å^2)	(Not available) , (Not available)	EDS
L-test for twinning[2]	$< \|L\| > = 0.49$, $< L^2 > = 0.32$	Xtriage
Estimated twinning fraction	0.029 for h,-k,-l	Xtriage
F_o,F_c correlation	0.95	EDS
Total number of atoms	5574	wwPDB-VP
Average B, all atoms (Å^2)	34.0	wwPDB-VP

Xtriage's analysis on translational NCS is as follows: *The largest off-origin peak in the Patterson function is 5.41% of the height of the origin peak. No significant pseudotranslation is detected.*

[1] Intensities estimated from amplitudes.

[2] Theoretical values of $< |L| >$, $< L^2 >$ for acentric reflections are 0.5, 0.333 respectively for untwinned datasets, and 0.375, 0.2 for perfectly twinned datasets.

For Manuscript Review

5 Model quality (i)

5.1 Standard geometry (i)

Bond lengths and bond angles in the following residue types are not validated in this section: 1JW, PEG, NGA, EDO, PGE

The Z score for a bond length (or angle) is the number of standard deviations the observed value is removed from the expected value. A bond length (or angle) with $|Z| > 5$ is considered an outlier worth inspection. RMSZ is the root-mean-square of all Z scores of the bond lengths (or angles).

Mol	Chain	Bond lengths		Bond angles	
		RMSZ	#$|Z|$ >5	RMSZ	#$|Z|$ >5
1	AAA	0.80	0/2580	0.96	3/3508 (0.1%)
1	BBB	0.92	0/2568	1.03	3/3488 (0.1%)
All	All	0.86	0/5148	1.00	6/6996 (0.1%)

There are no bond length outliers.

All (6) bond angle outliers are listed below:

Mol	Chain	Res	Type	Atoms	Z	Observed(^{o})	Ideal(^{o})
1	BBB	308	ARG	NE-CZ-NH2	-5.49	117.56	120.30
1	AAA	39[A]	MSE	CG-SE-CE	5.44	110.87	98.90
1	AAA	39[B]	MSE	CG-SE-CE	5.44	110.87	98.90
1	BBB	116	ARG	NE-CZ-NH1	-5.11	117.74	120.30
1	BBB	116	ARG	NE-CZ-NH2	5.06	122.83	120.30
1	AAA	318	TYR	CB-CA-C	5.05	120.49	110.40

There are no chirality outliers.

There are no planarity outliers.

5.2 Too-close contacts (i)

In the following table, the Non-H and H(model) columns list the number of non-hydrogen atoms and hydrogen atoms in the chain respectively. The H(added) column lists the number of hydrogen atoms added and optimized by MolProbity. The Clashes column lists the number of clashes within the asymmetric unit, whereas Symm-Clashes lists symmetry-related clashes.

Mol	Chain	Non-H	H(model)	H(added)	Clashes	Symm-Clashes
1	AAA	2521	0	2428	16	0
1	BBB	2511	0	2411	16	0
2	AAA	15	0	15	0	0

Continued on next page...

For Manuscript Review

Continued from previous page...

Mol	Chain	Non-H	H(model)	H(added)	Clashes	Symm-Clashes
3	AAA	4	0	6	0	0
3	BBB	8	0	12	0	0
4	AAA	7	0	10	0	0
5	BBB	27	0	0	0	0
6	BBB	10	0	14	1	0
7	AAA	189	0	0	3	0
7	BBB	282	0	0	5	0
All	All	5574	0	4896	33	0

The all-atom clashscore is defined as the number of clashes found per 1000 atoms (including hydrogen atoms). The all-atom clashscore for this structure is 3.

All (33) close contacts within the same asymmetric unit are listed below, sorted by their clash magnitude.

Atom-1	Atom-2	Interatomic distance (Å)	Clash overlap (Å)
6:BBB:405:PGE:H32	7:BBB:726:HOH:O	1.90	0.72
1:BBB:15:VAL:HG11	1:BBB:306:VAL:HB	1.74	0.69
1:BBB:130:PRO:HD3	7:BBB:648:HOH:O	2.05	0.57
1:AAA:92:GLU:OE1	1:AAA:131:HIS:ND1	2.40	0.50
1:BBB:201:ILE:HB	1:BBB:281:ILE:CD1	2.41	0.50
1:BBB:201:ILE:HB	1:BBB:281:ILE:HD12	1.94	0.50
1:AAA:286:ARG:HG3	1:AAA:319:ASP:HB2	1.94	0.49
1:BBB:296:MSE:O	1:BBB:307:GLN:HA	2.12	0.49
1:BBB:168:ILE:HG13	1:BBB:231:VAL:HG21	1.95	0.49
1:AAA:205:LEU:HD12	1:AAA:267:ILE:CD1	2.43	0.49
1:AAA:107:MSE:HE3	1:AAA:122:THR:HB	1.95	0.48
1:AAA:260:THR:O	1:AAA:260:THR:OG1	2.32	0.47
1:AAA:116[A]:ARG:NE	7:AAA:501:HOH:O	2.30	0.46
1:BBB:269:GLU:HB2	1:BBB:281:ILE:CD1	2.47	0.45
1:AAA:296:MSE:O	1:AAA:307:GLN:HA	2.17	0.45
1:AAA:116[B]:ARG:NE	7:AAA:501:HOH:O	2.31	0.44
1:AAA:230:GLU:OE1	1:AAA:242:SER:OG	2.22	0.44
1:BBB:269:GLU:HB2	1:BBB:281:ILE:HD11	2.00	0.44
1:BBB:231:VAL:HG23	7:BBB:616:HOH:O	2.18	0.44
1:AAA:129:HIS:CD2	1:AAA:146:TYR:HB3	2.53	0.43
1:BBB:286:ARG:O	1:BBB:317:HIS:HA	2.19	0.43
1:BBB:240:LYS:NZ	7:BBB:689[B]:HOH:O	2.52	0.43
1:BBB:312:VAL:HG12	7:BBB:631:HOH:O	2.19	0.42
1:AAA:26:GLN:HG3	1:AAA:152:SER:HA	2.01	0.42
1:BBB:215:LEU:HG	1:BBB:245:PHE:CE1	2.55	0.42
1:AAA:56:ARG:HB2	7:AAA:513:HOH:O	2.19	0.42

Continued on next page...

For Manuscript Review

Continued from previous page...

Atom-1	Atom-2	Interatomic distance (Å)	Clash overlap (Å)
1:AAA:205:LEU:HD12	1:AAA:267:ILE:HD13	2.02	0.42
1:AAA:163:LEU:O	1:AAA:225:VAL:HA	2.20	0.41
1:AAA:213:ALA:HB3	1:AAA:243:VAL:HG11	2.02	0.41
1:BBB:111:VAL:O	1:BBB:117:THR:HA	2.21	0.41
1:AAA:149:TYR:HA	1:AAA:153:LEU:HD12	2.03	0.41
1:BBB:164:PHE:O	1:BBB:298:LEU:HA	2.20	0.40
1:BBB:6:GLU:C	1:BBB:8:ALA:H	2.24	0.40

There are no symmetry-related clashes.

5.3 Torsion angles (i)

5.3.1 Protein backbone (i)

In the following table, the Percentiles column shows the percent Ramachandran outliers of the chain as a percentile score with respect to all X-ray entries followed by that with respect to entries of similar resolution.

The Analysed column shows the number of residues for which the backbone conformation was analysed, and the total number of residues.

Mol	Chain	Analysed	Favoured	Allowed	Outliers	Percentiles	
1	AAA	325/350 (93%)	316 (97%)	9 (3%)	0	100	100
1	BBB	322/350 (92%)	310 (96%)	12 (4%)	0	100	100
All	All	647/700 (92%)	626 (97%)	21 (3%)	0	100	100

There are no Ramachandran outliers to report.

5.3.2 Protein sidechains (i)

In the following table, the Percentiles column shows the percent sidechain outliers of the chain as a percentile score with respect to all X-ray entries followed by that with respect to entries of similar resolution.

The Analysed column shows the number of residues for which the sidechain conformation was analysed, and the total number of residues.

Mol	Chain	Analysed	Rotameric	Outliers	Percentiles	
1	AAA	276/301 (92%)	266 (96%)	10 (4%)	35	45

Continued on next page...

For Manuscript Review

Continued from previous page...

Mol	Chain	Analysed	Rotameric	Outliers	Percentiles	
1	BBB	275/301 (91%)	267 (97%)	8 (3%)	42	54
All	All	551/602 (92%)	533 (97%)	18 (3%)	38	49

All (18) residues with a non-rotameric sidechain are listed below:

Mol	Chain	Res	Type
1	AAA	85	LYS
1	AAA	104	PRO
1	AAA	177	GLU
1	AAA	200	SER
1	AAA	203	VAL
1	AAA	220	CYS
1	AAA	230	GLU
1	AAA	231	VAL
1	AAA	242	SER
1	AAA	244	GLN
1	BBB	5	PHE
1	BBB	100	SER
1	BBB	104	PRO
1	BBB	200	SER
1	BBB	203	VAL
1	BBB	208	SER
1	BBB	219	ARG
1	BBB	239	THR

Sometimes sidechains can be flipped to improve hydrogen bonding and reduce clashes. There are no such sidechains identified.

5.3.3 RNA (i)

There are no RNA molecules in this entry.

5.4 Non-standard residues in protein, DNA, RNA chains (i)

There are no non-standard protein/DNA/RNA residues in this entry.

5.5 Carbohydrates (i)

There are no monosaccharides in this entry.

For Manuscript Review

5.6 Ligand geometry (i)

9 ligands are modelled in this entry.

In the following table, the Counts columns list the number of bonds (or angles) for which Mogul statistics could be retrieved, the number of bonds (or angles) that are observed in the model and the number of bonds (or angles) that are defined in the Chemical Component Dictionary. The Link column lists molecule types, if any, to which the group is linked. The Z score for a bond length (or angle) is the number of standard deviations the observed value is removed from the expected value. A bond length (or angle) with $|Z| > 2$ is considered an outlier worth inspection. RMSZ is the root-mean-square of all Z scores of the bond lengths (or angles).

Mol	Type	Chain	Res	Link	Bond lengths			Bond angles		
					Counts	RMSZ	$\#\|Z\| > 2$	Counts	RMSZ	$\#\|Z\| > 2$
5	1JW	BBB	401	-	8,8,8	0.52	0	7,8,8	1.54	1 (14%)
4	PEG	AAA	403	-	6,6,6	0.48	0	5,5,5	0.44	0
3	EDO	AAA	402	-	3,3,3	0.29	0	2,2,2	0.17	0
5	1JW	BBB	402	-	8,8,8	0.84	1 (12%)	7,8,8	0.72	0
5	1JW	BBB	403	-	8,8,8	0.67	0	7,8,8	0.67	0
6	PGE	BBB	405	-	9,9,9	1.02	0	8,8,8	0.67	0
3	EDO	BBB	406	-	3,3,3	0.58	0	2,2,2	0.33	0
2	NGA	AAA	401	-	15,15,15	1.14	1 (6%)	21,21,21	1.58	5 (23%)
3	EDO	BBB	404	-	3,3,3	0.46	0	2,2,2	0.34	0

In the following table, the Chirals column lists the number of chiral outliers, the number of chiral centers analysed, the number of these observed in the model and the number defined in the Chemical Component Dictionary. Similar counts are reported in the Torsion and Rings columns. '-' means no outliers of that kind were identified.

Mol	Type	Chain	Res	Link	Chirals	Torsions	Rings
5	1JW	BBB	401	-	-	5/7/7/7	-
4	PEG	AAA	403	-	-	3/4/4/4	-
3	EDO	AAA	402	-	-	1/1/1/1	-
5	1JW	BBB	402	-	-	4/7/7/7	-
5	1JW	BBB	403	-	-	0/7/7/7	-
6	PGE	BBB	405	-	-	4/7/7/7	-
3	EDO	BBB	406	-	-	1/1/1/1	-
2	NGA	AAA	401	-	-	0/6/26/26	0/1/1/1
3	EDO	BBB	404	-	-	0/1/1/1	-

All (2) bond length outliers are listed below:

Mol	Chain	Res	Type	Atoms	Z	Observed(Å)	Ideal(Å)
2	AAA	401	NGA	C1-C2	2.37	1.55	1.52

Continued on next page...

For Manuscript Review

Continued from previous page...

Mol	Chain	Res	Type	Atoms	Z	Observed(Å)	Ideal(Å)
5	BBB	402	1JW	O09-C06	2.08	1.49	1.43

All (6) bond angle outliers are listed below:

Mol	Chain	Res	Type	Atoms	Z	Observed(^{o})	Ideal(^{o})
2	AAA	401	NGA	C1-C2-N2	3.94	115.29	110.73
5	BBB	401	1JW	O09-C06-C05	2.90	117.50	109.21
2	AAA	401	NGA	C1-O5-C5	-2.84	108.30	113.66
2	AAA	401	NGA	C3-C4-C5	-2.23	106.26	110.24
2	AAA	401	NGA	O1-C1-C2	-2.08	104.89	109.22
2	AAA	401	NGA	O1-C1-O5	2.03	116.48	110.38

There are no chirality outliers.

All (18) torsion outliers are listed below:

Mol	Chain	Res	Type	Atoms
5	BBB	401	1JW	C05-C06-C07-O08
5	BBB	402	1JW	C01-C05-C06-O09
6	BBB	405	PGE	O2-C3-C4-O3
5	BBB	402	1JW	C01-C05-C06-C07
6	BBB	405	PGE	O3-C5-C6-O4
4	AAA	403	PEG	O1-C1-C2-O2
5	BBB	401	1JW	O09-C06-C07-O08
5	BBB	402	1JW	C05-C01-C02-C03
3	BBB	406	EDO	O1-C1-C2-O2
5	BBB	402	1JW	C02-C01-C05-C06
4	AAA	403	PEG	C1-C2-O2-C3
5	BBB	401	1JW	C01-C05-C06-C07
5	BBB	401	1JW	C02-C01-C05-C06
6	BBB	405	PGE	C3-C4-O3-C5
5	BBB	401	1JW	C01-C05-C06-O09
6	BBB	405	PGE	O1-C1-C2-O2
3	AAA	402	EDO	O1-C1-C2-O2
4	AAA	403	PEG	O2-C3-C4-O4

There are no ring outliers.

1 monomer is involved in 1 short contact:

Mol	Chain	Res	Type	Clashes	Symm-Clashes
6	BBB	405	PGE	1	0

For Manuscript Review

The following is a two-dimensional graphical depiction of Mogul quality analysis of bond lengths, bond angles, torsion angles, and ring geometry for all instances of the Ligand of Interest. In addition, ligands with molecular weight > 250 and outliers as shown on the validation Tables will also be included. For torsion angles, if less then 5% of the Mogul distribution of torsion angles is within 10 degrees of the torsion angle in question, then that torsion angle is considered an outlier. Any bond that is central to one or more torsion angles identified as an outlier by Mogul will be highlighted in the graph. For rings, the root-mean-square deviation (RMSD) between the ring in question and similar rings identified by Mogul is calculated over all ring torsion angles. If the average RMSD is greater than 60 degrees and the minimal RMSD between the ring in question and any Mogul-identified rings is also greater than 60 degrees, then that ring is considered an outlier. The outliers are highlighted in purple. The color gray indicates Mogul did not find sufficient equivalents in the CSD to analyse the geometry.

Ligand NGA AAA 401

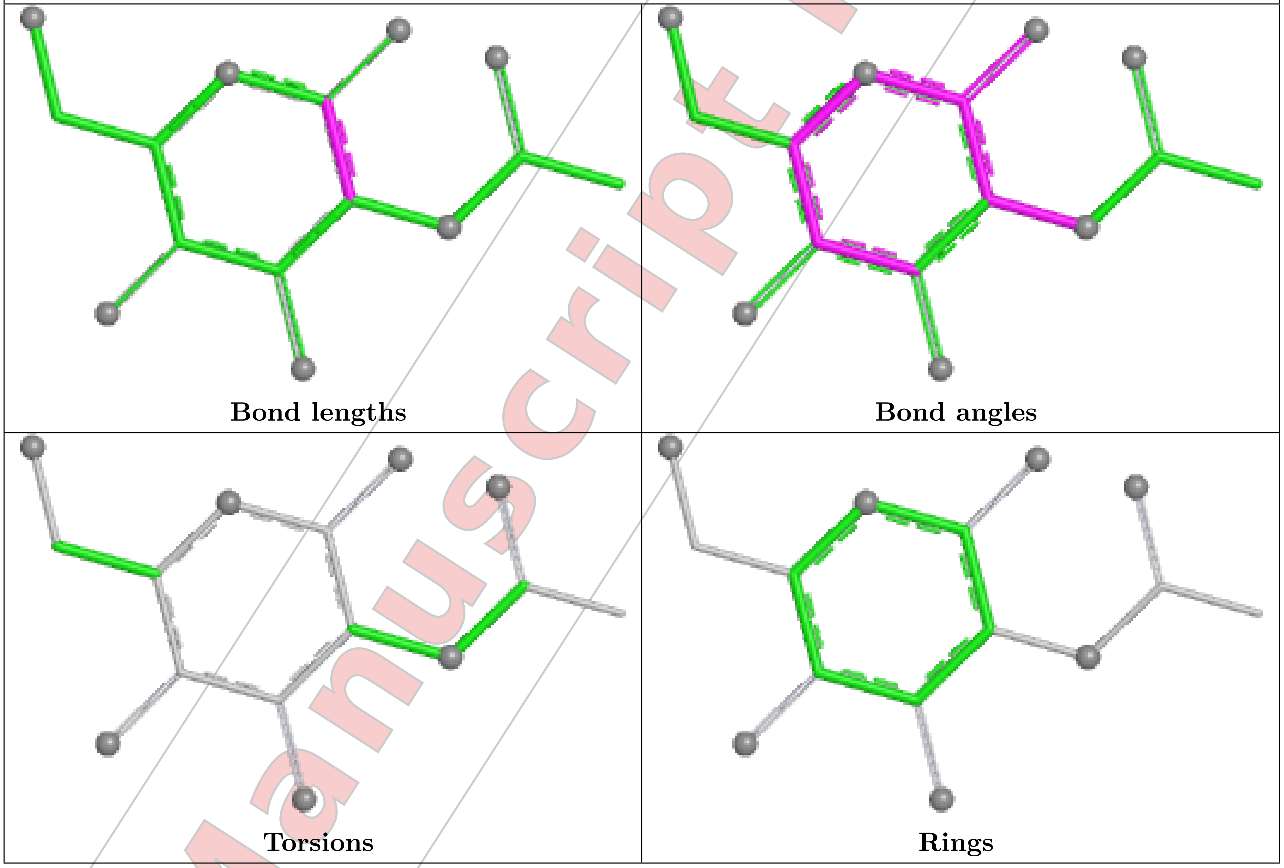

5.7 Other polymers (i)

There are no such residues in this entry.

5.8 Polymer linkage issues (i)

There are no chain breaks in this entry.

For Manuscript Review

6 Fit of model and data (i)

6.1 Protein, DNA and RNA chains (i)

In the following table, the column labelled '#RSRZ> 2' contains the number (and percentage) of RSRZ outliers, followed by percent RSRZ outliers for the chain as percentile scores relative to all X-ray entries and entries of similar resolution. The OWAB column contains the minimum, median, 95th percentile and maximum values of the occupancy-weighted average B-factor per residue. The column labelled 'Q< 0.9' lists the number of (and percentage) of residues with an average occupancy less than 0.9.

Mol	Chain	Analysed	<RSRZ>	#RSRZ>2			OWAB($Å^2$)	Q<0.9
1	AAA	321/350 (91%)	0.88	38 (11%)	4	4	21, 36, 60, 86	0
1	BBB	318/350 (90%)	0.87	36 (11%)	5	4	11, 26, 63, 80	0
All	All	639/700 (91%)	0.87	74 (11%)	4	4	11, 32, 61, 86	0

All (74) RSRZ outliers are listed below:

Mol	Chain	Res	Type	RSRZ
1	AAA	303	GLY	6.2
1	BBB	227	SER	6.1
1	AAA	259	ARG	5.6
1	AAA	326	PRO	5.3
1	BBB	274	PRO	4.9
1	AAA	327	VAL	4.7
1	AAA	202	SER	4.4
1	BBB	8	ALA	4.2
1	BBB	275	PRO	4.1
1	AAA	268	ASN	3.7
1	BBB	266	GLN	3.6
1	AAA	328	THR	3.5
1	BBB	194	ASP	3.5
1	AAA	295	THR	3.5
1	BBB	326	PRO	3.3
1	AAA	246	SER	3.1
1	BBB	262	ALA	3.1
1	BBB	153	LEU	3.0
1	BBB	193	THR	3.0
1	BBB	128	VAL	3.0
1	BBB	202	SER	3.0
1	BBB	7	PRO	3.0
1	BBB	192	ASP	3.0
1	BBB	185	LEU	3.0

Continued on next page...

For Manuscript Review

Continued from previous page...

Mol	Chain	Res	Type	RSRZ
1	BBB	246	SER	2.9
1	AAA	196	PRO	2.9
1	BBB	272	THR	2.8
1	BBB	325	VAL	2.8
1	BBB	6	GLU	2.8
1	BBB	261	SER	2.8
1	AAA	325	VAL	2.8
1	BBB	199	THR	2.8
1	AAA	302	SER	2.7
1	AAA	227	SER	2.7
1	BBB	259	ARG	2.7
1	AAA	166	ASP	2.7
1	AAA	230	GLU	2.6
1	BBB	260	THR	2.6
1	AAA	174	GLN	2.6
1	AAA	226	GLY	2.6
1	AAA	266	GLN	2.6
1	AAA	194	ASP	2.5
1	BBB	197	VAL	2.5
1	BBB	226	GLY	2.5
1	AAA	192	ASP	2.5
1	AAA	193	THR	2.5
1	BBB	201	ILE	2.5
1	AAA	244	GLN	2.4
1	AAA	324	VAL	2.4
1	AAA	4	THR	2.4
1	AAA	44	THR	2.4
1	AAA	272	THR	2.3
1	AAA	225	VAL	2.3
1	AAA	241	VAL	2.3
1	BBB	31	VAL	2.3
1	AAA	185	LEU	2.3
1	AAA	270	HIS	2.3
1	BBB	5	PHE	2.2
1	AAA	67	ALA	2.2
1	AAA	240	LYS	2.2
1	AAA	203	VAL	2.2
1	AAA	243	VAL	2.2
1	BBB	190	ARG	2.2
1	BBB	273	ILE	2.2
1	AAA	195	GLN	2.1
1	BBB	277	LYS	2.1

Continued on next page...

For Manuscript Review

Continued from previous page...

Mol	Chain	Res	Type	RSRZ
1	BBB	267	ILE	2.1
1	BBB	47	VAL	2.1
1	BBB	271	VAL	2.1
1	BBB	9	THR	2.0
1	BBB	67	ALA	2.0
1	AAA	25	VAL	2.0
1	AAA	274	PRO	2.0
1	AAA	281	ILE	2.0

6.2 Non-standard residues in protein, DNA, RNA chains (i)

There are no non-standard protein/DNA/RNA residues in this entry.

6.3 Carbohydrates (i)

There are no monosaccharides in this entry.

6.4 Ligands (i)

In the following table, the Atoms column lists the number of modelled atoms in the group and the number defined in the chemical component dictionary. The B-factors column lists the minimum, median, 95th percentile and maximum values of B factors of atoms in the group. The column labelled ‘Q< 0.9’ lists the number of atoms with occupancy less than 0.9.

Mol	Type	Chain	Res	Atoms	RSCC	RSR	B-factors(Å^2)	Q<0.9
6	PGE	BBB	405	10/10	0.62	0.26	39,53,70,71	0
3	EDO	BBB	404	4/4	0.70	0.26	44,47,49,54	0
5	1JW	BBB	402	9/9	0.80	0.30	28,46,67,69	0
5	1JW	BBB	401	9/9	0.82	0.19	18,28,45,52	0
4	PEG	AAA	403	7/7	0.86	0.18	31,38,48,51	0
2	NGA	AAA	401	15/15	0.87	0.16	34,40,50,55	0
3	EDO	AAA	402	4/4	0.88	0.21	39,46,47,54	0
3	EDO	BBB	406	4/4	0.90	0.20	29,41,47,51	0
5	1JW	BBB	403	9/9	0.92	0.23	18,22,26,28	0

The following is a graphical depiction of the model fit to experimental electron density of all instances of the Ligand of Interest. In addition, ligands with molecular weight > 250 and outliers as shown on the geometry validation Tables will also be included. Each fit is shown from different orientation to approximate a three-dimensional view.

For Manuscript Review

Electron density around NGA AAA 401:

$2mF_o$-DF_c (at 0.7 rmsd) in gray
mF_o-DF_c (at 3 rmsd) in purple (negative)
and green (positive)

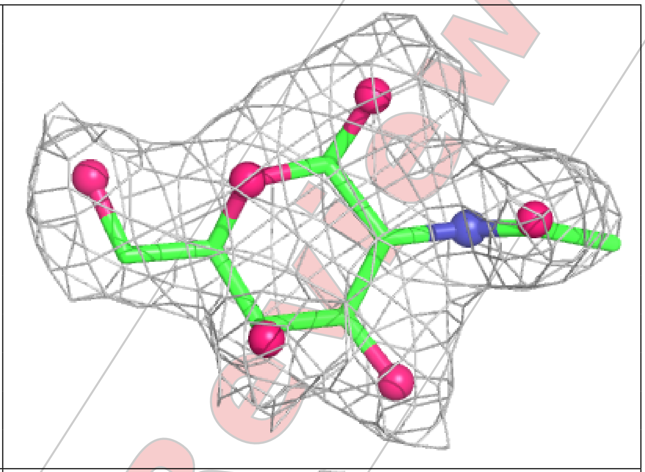

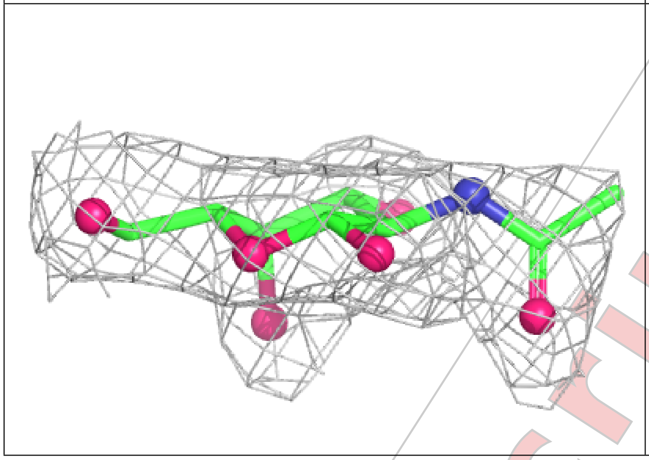

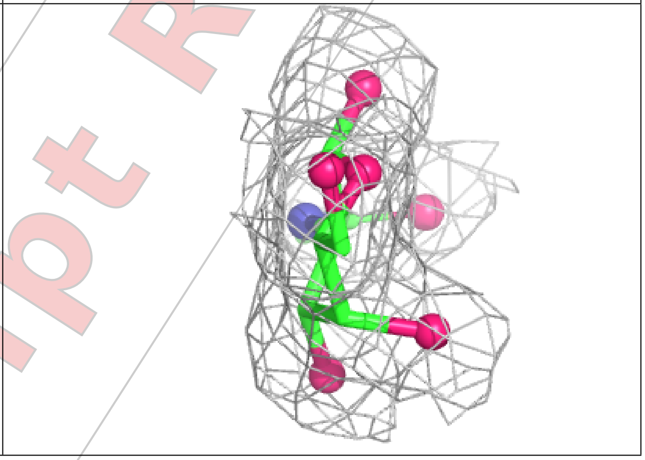

6.5 Other polymers (i)

There are no such residues in this entry.

For Manuscript Review
