# Supplementary material for: The choanoflagellate pore-forming lectin SaroL-1 punches holes in cancer cells by targeting the tumor-related glycosphingolipid Gb3
Source: Commun Biol. 2022 Sep 12;5:954. doi: 10.1038/s42003-022-03869-w (PMC9468336; doi:10.1038/s42003-022-03869-w)
Supplement: Supplementary file 6 — validation report 7QE4 [file 42003_2022_3869_MOESM6_ESM.pdf]

Full wwPDB X-ray Structure Validation Report (i)

Dec 1, 2021 – 12:19 pm GMT

PDB ID	:	7QE4
Title	:	B-trefoil lectin from Salpingoeca rosetta in complex with GalNAc
Deposited on	:	2021-12-01
Resolution	:	1.70 Å(reported)

This is a Full wwPDB X-ray Structure Validation Report.

This report is produced by the wwPDB biocuration pipeline after annotation of the structure.

We welcome your comments at *validation@mail.wwpdb.org*
A user guide is available at
https://www.wwpdb.org/validation/2017/XrayValidationReportHelp
with specific help available everywhere you see the (i) symbol.

CONFIDENTIAL VALIDATION REPORT

The following versions of software and data (see references (i)) were used in the production of this report:

MolProbity	:	4.02b-467
Mogul	:	1.8.4 (270009), CSD as541be (2020)
Xtriage (Phenix)	:	1.13
EDS	:	2.23.2
buster-report	:	1.1.7 (2018)
Percentile statistics	:	20191225.v01 (using entries in the PDB archive December 25th 2019)
Refmac	:	5.8.0267
CCP4	:	7.1.010 (Gargrove)
Ideal geometry (proteins)	:	Engh & Huber (2001)
Ideal geometry (DNA, RNA)	:	Parkinson et al. (1996)
Validation Pipeline (wwPDB-VP)	:	2.23.2

1 Overall quality at a glance (i)

The following experimental techniques were used to determine the structure:
X-RAY DIFFRACTION

The reported resolution of this entry is 1.70 Å.

Percentile scores (ranging between 0-100) for global validation metrics of the entry are shown in the following graphic. The table shows the number of entries on which the scores are based.

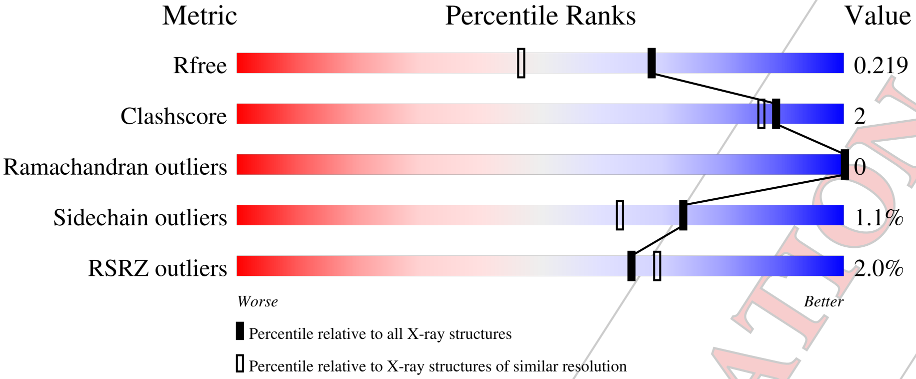

Metric	Whole archive (#Entries)	Similar resolution (#Entries, resolution range(Å))
R_{free}	130704	4298 (1.70-1.70)
Clashscore	141614	4695 (1.70-1.70)
Ramachandran outliers	138981	4610 (1.70-1.70)
Sidechain outliers	138945	4610 (1.70-1.70)
RSRZ outliers	127900	4222 (1.70-1.70)

The table below summarises the geometric issues observed across the polymeric chains and their fit to the electron density. The red, orange, yellow and green segments of the lower bar indicate the fraction of residues that contain outliers for >=3, 2, 1 and 0 types of geometric quality criteria respectively. A grey segment represents the fraction of residues that are not modelled. The numeric value for each fraction is indicated below the corresponding segment, with a dot representing fractions <=5% The upper red bar (where present) indicates the fraction of residues that have poor fit to the electron density. The numeric value is given above the bar.

Mol	Chain	Length	Quality of chain
1	AAA	329	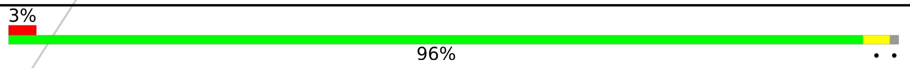
1	BBB	329	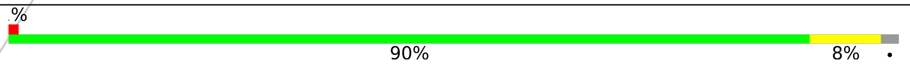

CONFIDENTIAL VALIDATION REPORT

2 Entry composition (i)

There are 6 unique types of molecules in this entry. The entry contains 11463 atoms, of which 5190 are hydrogens and 0 are deuteriums.

In the tables below, the ZeroOcc column contains the number of atoms modelled with zero occupancy, the AltConf column contains the number of residues with at least one atom in alternate conformation and the Trace column contains the number of residues modelled with at most 2 atoms.

- Molecule 1 is a protein called Sarol-1.

Mol	Chain	Residues	Atoms						ZeroOcc	AltConf	Trace
			Total	C	H	N	O	S			
1	AAA	325	5095	1611	2525	458	492	9	116	8	0
1	BBB	322	5115	1617	2545	458	488	7	111	11	0

- Molecule 2 is 2-acetamido-2-deoxy-alpha-D-galactopyranose (three-letter code: A2G) (formula: $C_8H_{15}NO_6$) (labeled as "Ligand of Interest" by depositor).

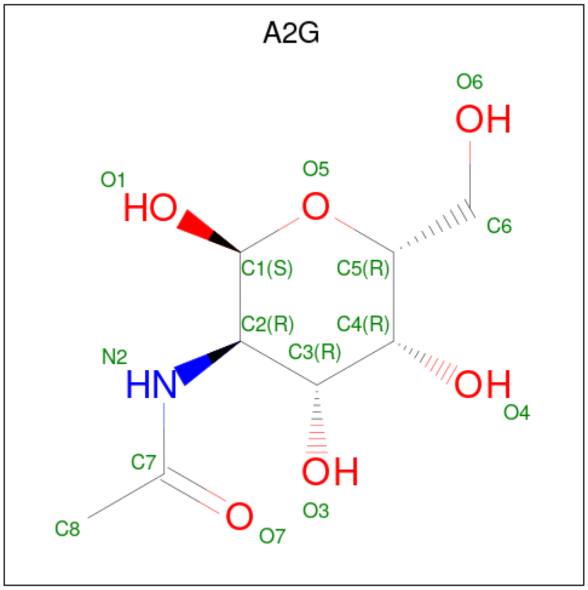

Mol	Chain	Residues	Atoms					ZeroOcc	AltConf
			Total	C	H	N	O		
2	AAA	1	30	8	15	1	6	4	0
2	AAA	1	30	8	15	1	6	4	1
2	AAA	1	30	8	15	1	6	4	0
2	BBB	1	30	8	15	1	6	4	0

Continued on next page...

CONFIDENTIAL VALIDATION REPORT

Continued from previous page...

Mol	Chain	Residues	Atoms					ZeroOcc	AltConf
2	BBB	1	Total<br>30	C<br>8	H<br>15	N<br>1	O<br>6	4	0
2	BBB	1	Total<br>30	C<br>8	H<br>15	N<br>1	O<br>6	4	1

- Molecule 3 is 2-acetamido-2-deoxy-beta-D-galactopyranose (three-letter code: NGA) (formula: $C_8H_{15}NO_6$) (labeled as "Ligand of Interest" by depositor).

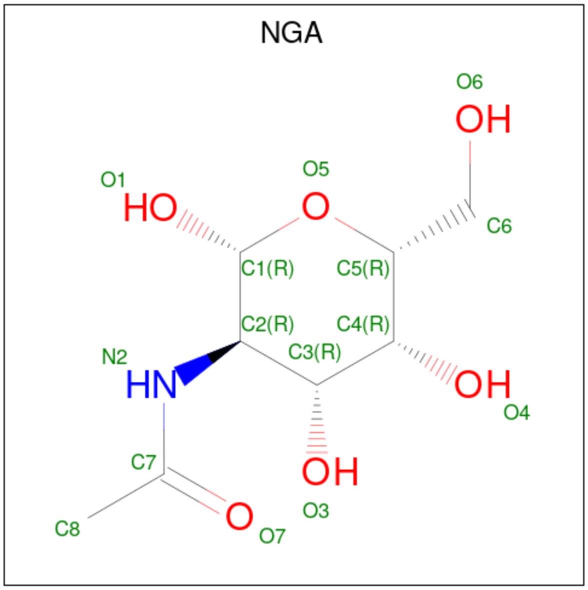

Mol	Chain	Residues	Atoms					ZeroOcc	AltConf
3	AAA	1	Total<br>30	C<br>8	H<br>15	N<br>1	O<br>6	3	1
3	BBB	1	Total<br>30	C<br>8	H<br>15	N<br>1	O<br>6	3	1

- Molecule 4 is CHLORIDE ION (three-letter code: CL) (formula: Cl).

Mol	Chain	Residues	Atoms		ZeroOcc	AltConf
4	AAA	1	Total<br>1	Cl<br>1	0	0

- Molecule 5 is SODIUM ION (three-letter code: NA) (formula: Na).

Mol	Chain	Residues	Atoms		ZeroOcc	AltConf
5	BBB	2	Total<br>2	Na<br>2	0	0

CONFIDENTIAL VALIDATION REPORT

- Molecule 6 is water.

Mol	Chain	Residues	Atoms	ZeroOcc	AltConf
6	AAA	474	Total O<br>486 486	0	12
6	BBB	503	Total O<br>524 524	0	21

CONFIDENTIAL VALIDATION REPORT

3 Residue-property plots (i)

These plots are drawn for all protein, RNA, DNA and oligosaccharide chains in the entry. The first graphic for a chain summarises the proportions of the various outlier classes displayed in the second graphic. The second graphic shows the sequence view annotated by issues in geometry and electron density. Residues are color-coded according to the number of geometric quality criteria for which they contain at least one outlier: green = 0, yellow = 1, orange = 2 and red = 3 or more. A red dot above a residue indicates a poor fit to the electron density (RSRZ > 2). Stretches of 2 or more consecutive residues without any outlier are shown as a green connector. Residues present in the sample, but not in the model, are shown in grey.

- Molecule 1: Sarol-1

Chain AAA:

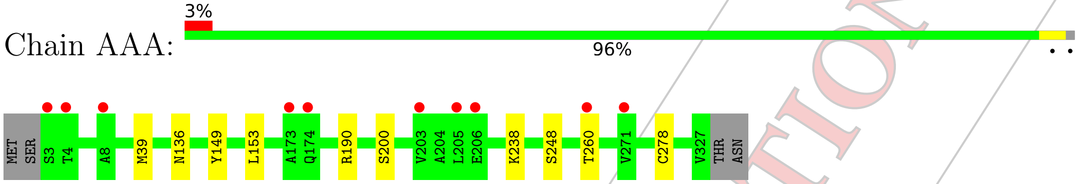

- Molecule 1: Sarol-1

Chain BBB:

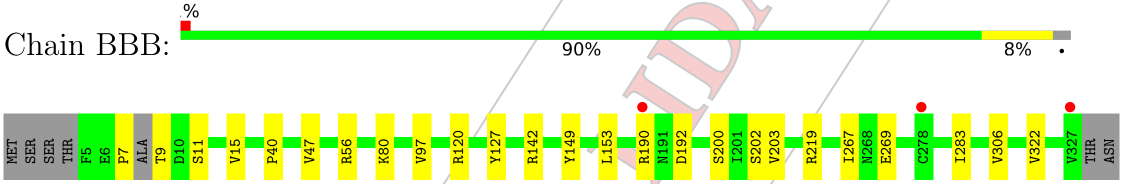

CONFIDENTIAL VALIDATION REPORT

4 Data and refinement statistics (i)

Property	Value	Source
Space group	P 21 21 21	Depositor
Cell constants<br>a, b, c, α, β, γ	57.16Å 59.25Å 209.61Å<br>90.00° 90.00° 90.00°	Depositor
Resolution (Å)	44.28 – 1.70<br>44.24 – 1.70	Depositor<br>EDS
% Data completeness<br>(in resolution range)	100.0 (44.28-1.70)<br>99.9 (44.24-1.70)	Depositor<br>EDS
R_{merge}	0.08	Depositor
R_{sym}	(Not available)	Depositor
$< I/\sigma(I) >$ [1]	2.34 (at 1.70Å)	Xtriage
Refinement program	REFMAC 5.8.0267, REFMAC 5.8.0267	Depositor
R, R_{free}	0.169 , 0.206<br>0.180 , 0.219	Depositor<br>DCC
R_{free} test set	3912 reflections (4.93%)	wwPDB-VP
Wilson B-factor (Å^2)	18.0	Xtriage
Anisotropy	0.499	Xtriage
Bulk solvent k_{sol}(e/Å^3), B_{sol}(Å^2)	(Not available) , (Not available)	EDS
L-test for twinning[2]	$<\|L\|> = 0.49$, $< L^2 > = 0.32$	Xtriage
Estimated twinning fraction	0.000 for k,h,-l	Xtriage
F_o,F_c correlation	0.97	EDS
Total number of atoms	11463	wwPDB-VP
Average B, all atoms (Å^2)	20.0	wwPDB-VP

Xtriage's analysis on translational NCS is as follows: *The analyses of the Patterson function reveals a significant off-origin peak that is 37.75 % of the origin peak, indicating pseudo-translational symmetry. The chance of finding a peak of this or larger height randomly in a structure without pseudo-translational symmetry is equal to 4.0888e-04. The detected translational NCS is most likely also responsible for the elevated intensity ratio.*

[1] Intensities estimated from amplitudes.

[2] Theoretical values of $<|L|>$, $< L^2 >$ for acentric reflections are 0.5, 0.333 respectively for untwinned datasets, and 0.375, 0.2 for perfectly twinned datasets.

CONFIDENTIAL VALIDATION REPORT

5 Model quality (i)

5.1 Standard geometry (i)

Bond lengths and bond angles in the following residue types are not validated in this section: CL, NA, A2G, NGA

The Z score for a bond length (or angle) is the number of standard deviations the observed value is removed from the expected value. A bond length (or angle) with $|Z| > 5$ is considered an outlier worth inspection. RMSZ is the root-mean-square of all Z scores of the bond lengths (or angles).

Mol	Chain	Bond lengths		Bond angles	
		RMSZ	$\#\|Z\|>5$	RMSZ	$\#\|Z\|>5$
1	AAA	0.64	0/2633	0.79	0/3582
1	BBB	0.63	0/2650	0.78	0/3603
All	All	0.64	0/5283	0.78	0/7185

There are no bond length outliers.

There are no bond angle outliers.

There are no chirality outliers.

There are no planarity outliers.

5.2 Too-close contacts (i)

In the following table, the Non-H and H(model) columns list the number of non-hydrogen atoms and hydrogen atoms in the chain respectively. The H(added) column lists the number of hydrogen atoms added and optimized by MolProbity. The Clashes column lists the number of clashes within the asymmetric unit, whereas Symm-Clashes lists symmetry-related clashes.

Mol	Chain	Non-H	H(model)	H(added)	Clashes	Symm-Clashes
1	AAA	2570	2525	2482	4	0
1	BBB	2570	2545	2515	16	0
2	AAA	45	45	36	0	0
2	BBB	45	45	36	0	0
3	AAA	15	15	15	0	0
3	BBB	15	15	15	0	0
4	AAA	1	0	0	0	0
5	BBB	2	0	0	0	0
6	AAA	486	0	0	1	0
6	BBB	524	0	0	1	0
All	All	6273	5190	5099	20	0

CONFIDENTIAL VALIDATION REPORT

The all-atom clashscore is defined as the number of clashes found per 1000 atoms (including hydrogen atoms). The all-atom clashscore for this structure is 2.

All (20) close contacts within the same asymmetric unit are listed below, sorted by their clash magnitude.

Atom-1	Atom-2	Interatomic distance (Å)	Clash overlap (Å)
1:BBB:40:PRO:HB2	1:BBB:97[A]:VAL:HG21	1.81	0.63
1:AAA:39[A]:MET:HA	1:AAA:39[A]:MET:HE3	1.82	0.62
1:BBB:120[B]:ARG:HD3	1:BBB:127:TYR:CZ	2.37	0.60
1:BBB:15[B]:VAL:HG11	1:BBB:306:VAL:HB	1.85	0.59
1:BBB:7:PRO:O	1:BBB:11:SER:OG	2.25	0.54
1:BBB:200:SER:HA	1:BBB:269:GLU:O	2.10	0.51
1:BBB:283[B]:ILE:HD12	1:BBB:322:VAL:HG22	1.93	0.50
1:BBB:202[B]:SER:HA	1:BBB:267:ILE:O	2.13	0.48
1:AAA:238:LYS:HD3	6:AAA:771:HOH:O	2.14	0.48
1:BBB:47:VAL:HG12	1:BBB:142:ARG:HD2	1.96	0.47
1:BBB:202[A]:SER:HA	1:BBB:267:ILE:O	2.15	0.47
1:BBB:80:LYS:HB2	1:BBB:97[B]:VAL:HG13	1.96	0.46
1:BBB:149:TYR:HA	1:BBB:153:LEU:HD12	1.98	0.45
1:BBB:203:VAL:HG11	1:BBB:283[A]:ILE:HD11	1.98	0.45
1:BBB:142:ARG:NH2	6:BBB:502:HOH:O	2.36	0.44
1:AAA:149:TYR:HA	1:AAA:153:LEU:HD12	1.98	0.44
1:BBB:120[B]:ARG:HD3	1:BBB:127:TYR:CE2	2.52	0.43
1:AAA:190[A]:ARG:CZ	1:AAA:278:CYS:HB2	2.48	0.43
1:BBB:190:ARG:HD2	1:BBB:192:ASP:OD1	2.19	0.41
1:BBB:11:SER:HB2	1:BBB:219:ARG:HH21	1.85	0.41

There are no symmetry-related clashes.

5.3 Torsion angles (i)

5.3.1 Protein backbone (i)

In the following table, the Percentiles column shows the percent Ramachandran outliers of the chain as a percentile score with respect to all X-ray entries followed by that with respect to entries of similar resolution.

The Analysed column shows the number of residues for which the backbone conformation was analysed, and the total number of residues.

Mol	Chain	Analysed	Favoured	Allowed	Outliers	Percentiles	
1	AAA	331/329 (101%)	325 (98%)	6 (2%)	0	100	100

Continued on next page...

CONFIDENTIAL VALIDATION REPORT

Continued from previous page...

Mol	Chain	Analysed	Favoured	Allowed	Outliers	Percentiles	
1	BBB	329/329 (100%)	321 (98%)	8 (2%)	0	100	100
All	All	660/658 (100%)	646 (98%)	14 (2%)	0	100	100

There are no Ramachandran outliers to report.

5.3.2 Protein sidechains (i)

In the following table, the Percentiles column shows the percent sidechain outliers of the chain as a percentile score with respect to all X-ray entries followed by that with respect to entries of similar resolution.

The Analysed column shows the number of residues for which the sidechain conformation was analysed, and the total number of residues.

Mol	Chain	Analysed	Rotameric	Outliers	Percentiles	
1	AAA	284/289 (98%)	280 (99%)	4 (1%)	67	53
1	BBB	288/289 (100%)	286 (99%)	2 (1%)	84	77
All	All	572/578 (99%)	566 (99%)	6 (1%)	73	67

All (6) residues with a non-rotameric sidechain are listed below:

Mol	Chain	Res	Type
1	AAA	136	ASN
1	AAA	200	SER
1	AAA	248	SER
1	AAA	260	THR
1	BBB	9	THR
1	BBB	56	ARG

Sometimes sidechains can be flipped to improve hydrogen bonding and reduce clashes. There are no such sidechains identified.

5.3.3 RNA (i)

There are no RNA molecules in this entry.

5.4 Non-standard residues in protein, DNA, RNA chains (i)

There are no non-standard protein/DNA/RNA residues in this entry.

CONFIDENTIAL VALIDATION REPORT

5.5 Carbohydrates (i)

There are no monosaccharides in this entry.

5.6 Ligand geometry (i)

Of 11 ligands modelled in this entry, 3 are monoatomic - leaving 8 for Mogul analysis.

There are no bond length outliers.

There are no bond angle outliers.

There are no chirality outliers.

There are no torsion outliers.

There are no ring outliers.

No monomer is involved in short contacts.

The following is a two-dimensional graphical depiction of Mogul quality analysis of bond lengths, bond angles, torsion angles, and ring geometry for all instances of the Ligand of Interest. In addition, ligands with molecular weight > 250 and outliers as shown on the validation Tables will also be included. For torsion angles, if less then 5% of the Mogul distribution of torsion angles is within 10 degrees of the torsion angle in question, then that torsion angle is considered an outlier. Any bond that is central to one or more torsion angles identified as an outlier by Mogul will be highlighted in the graph. For rings, the root-mean-square deviation (RMSD) between the ring in question and similar rings identified by Mogul is calculated over all ring torsion angles. If the average RMSD is greater than 60 degrees and the minimal RMSD between the ring in question and any Mogul-identified rings is also greater than 60 degrees, then that ring is considered an outlier. The outliers are highlighted in purple. The color gray indicates Mogul did not find sufficient equivalents in the CSD to analyse the geometry.

CONFIDENTIAL VALIDATION REPORT

Ligand A2G AAA 401

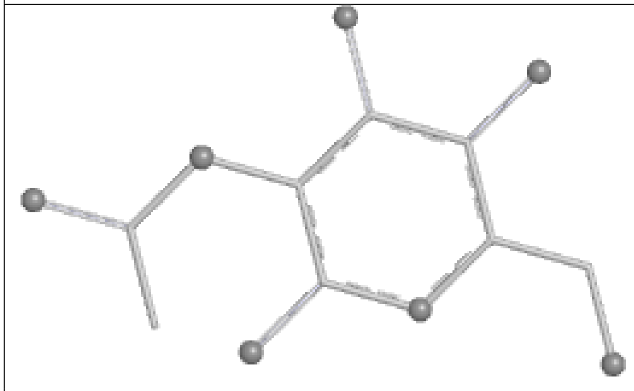
Bond lengths

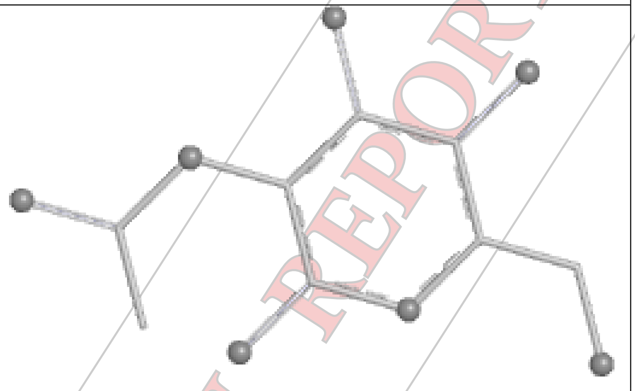
Bond angles

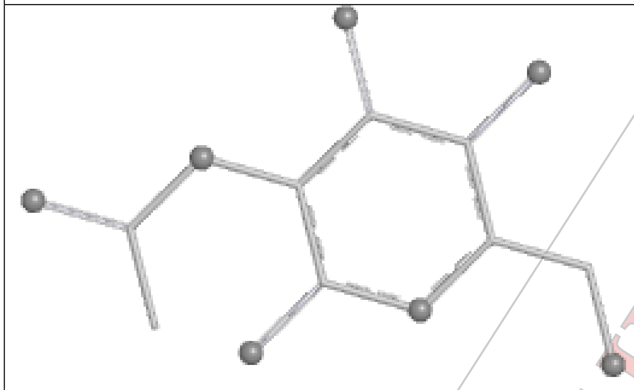
Torsions

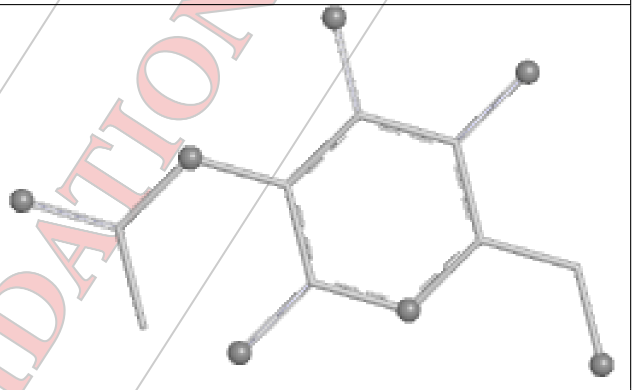
Rings

Ligand A2G AAA 402 (A)

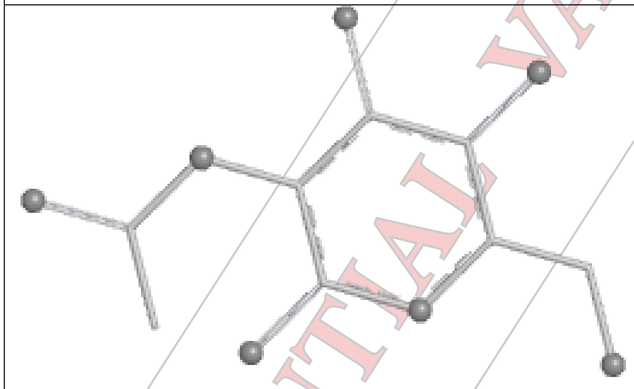
Bond lengths

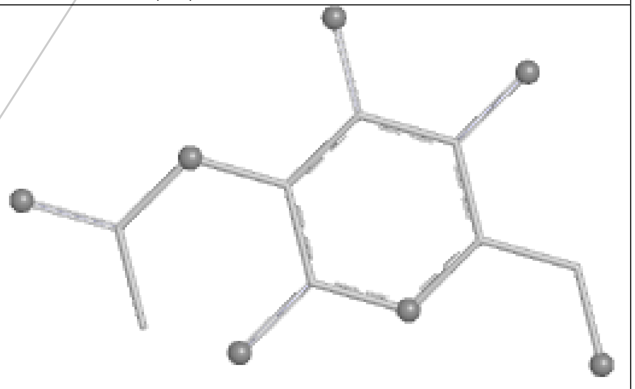
Bond angles

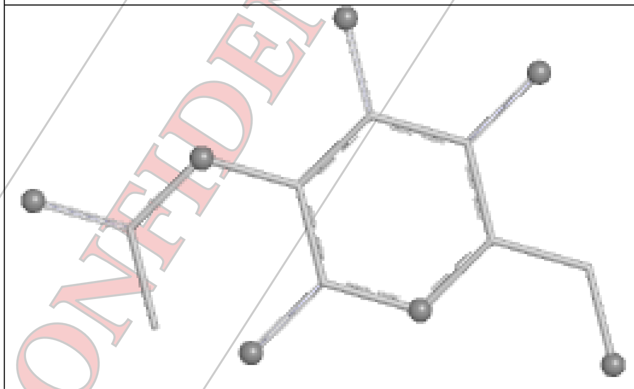
Torsions

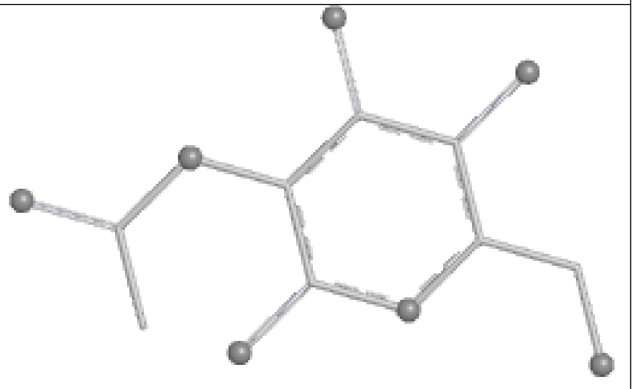
Rings

CONFIDENTIAL VALIDATION REPORT

Ligand A2G AAA 403

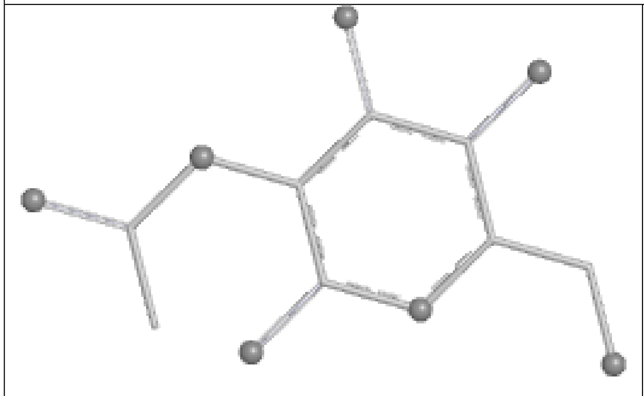

Bond lengths

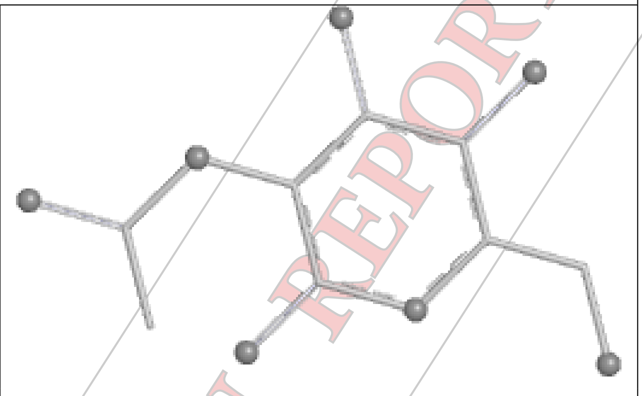

Bond angles

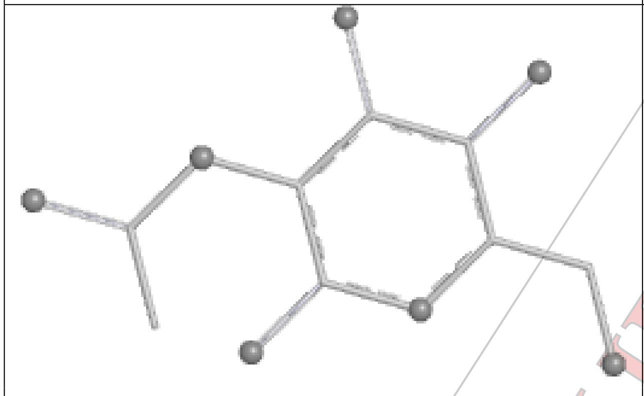

Torsions

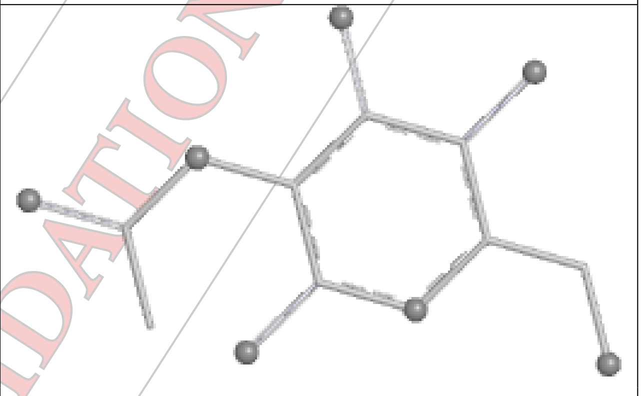

Rings

Ligand A2G BBB 401

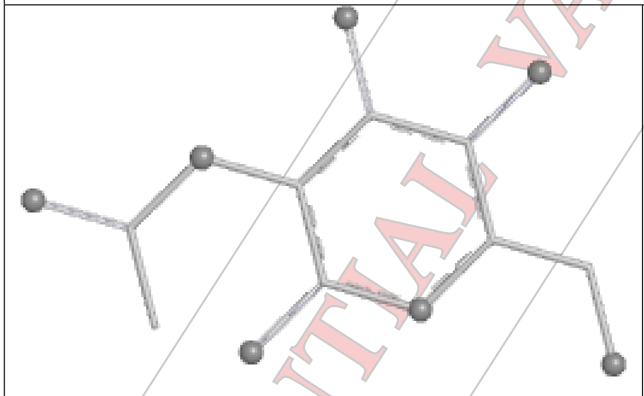

Bond lengths

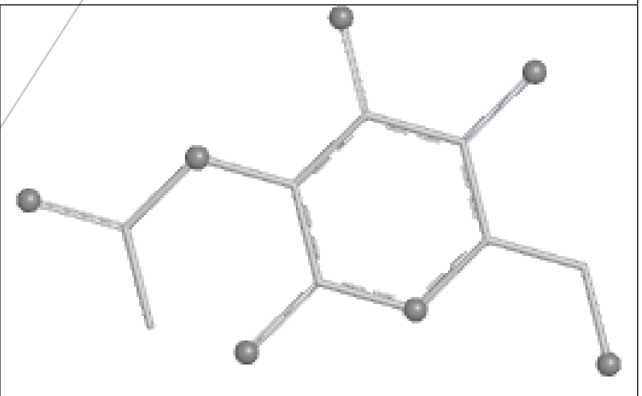

Bond angles

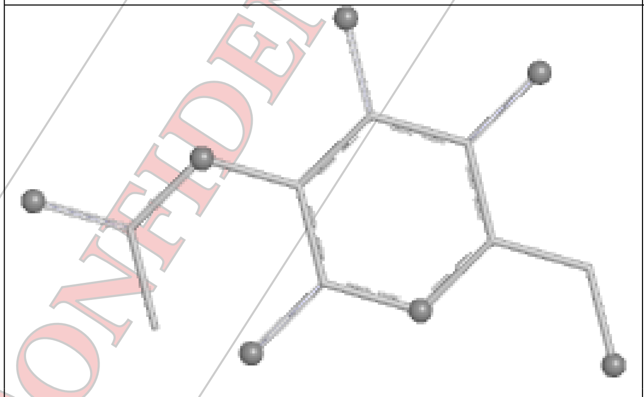

Torsions

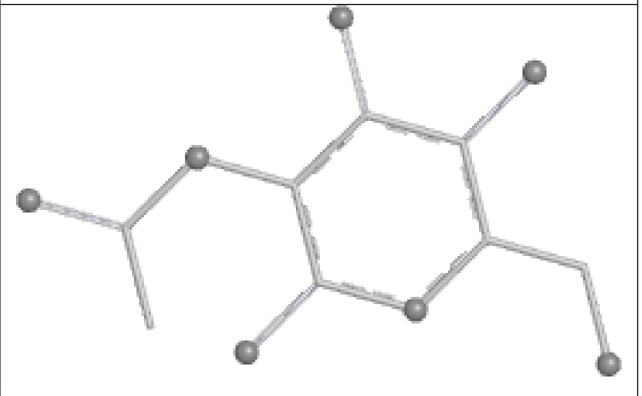

Rings

CONFIDENTIAL VALIDATION REPORT

Ligand A2G BBB 402

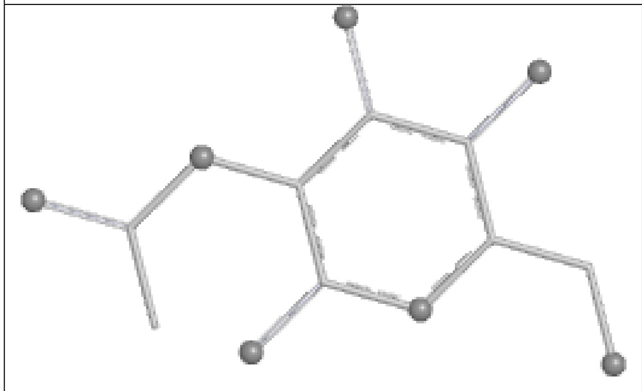

Bond lengths

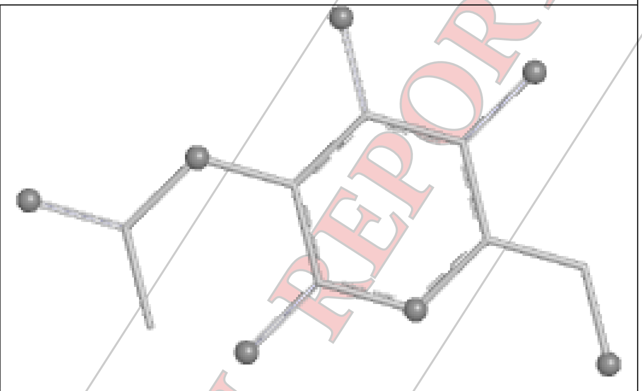

Bond angles

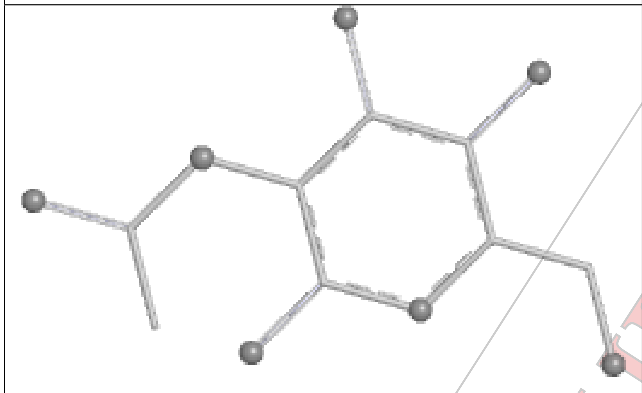

Torsions

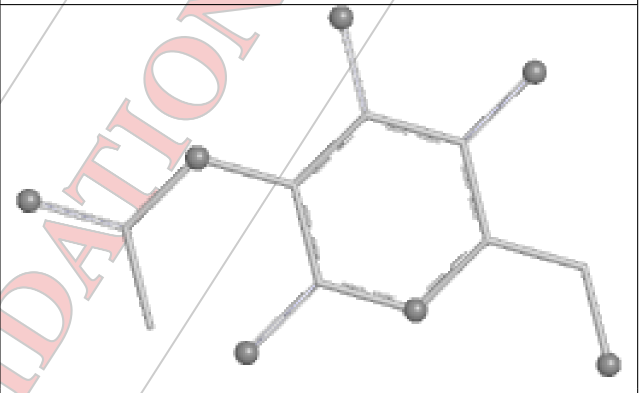

Rings

Ligand A2G BBB 403 (A)

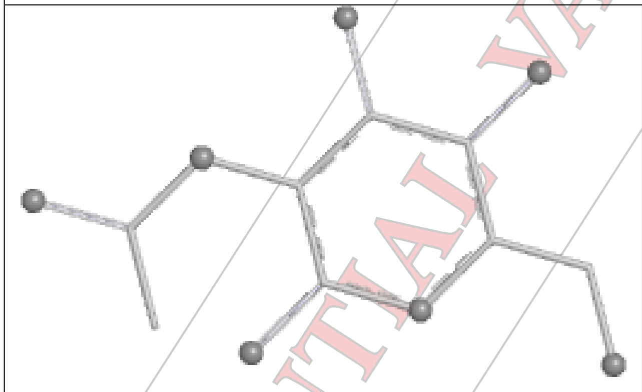

Bond lengths

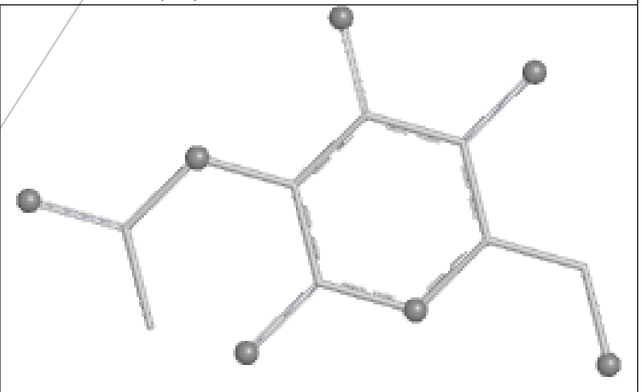

Bond angles

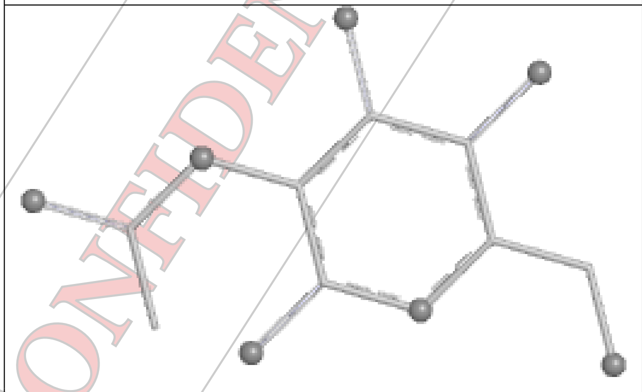

Torsions

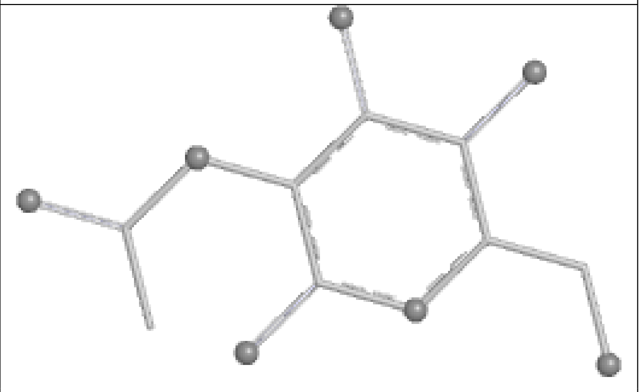

Rings

CONFIDENTIAL VALIDATION REPORT

Ligand NGA AAA 404 (B)

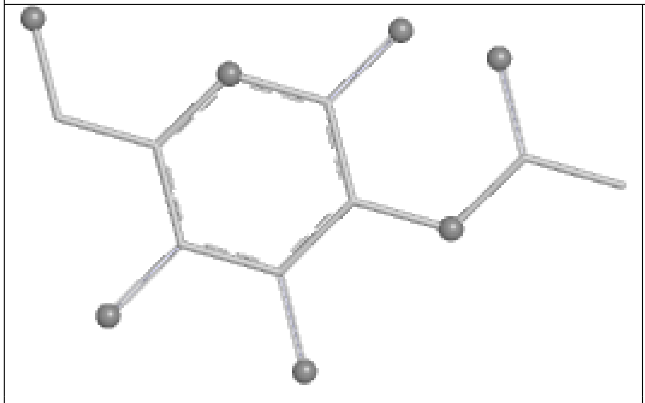

Bond lengths

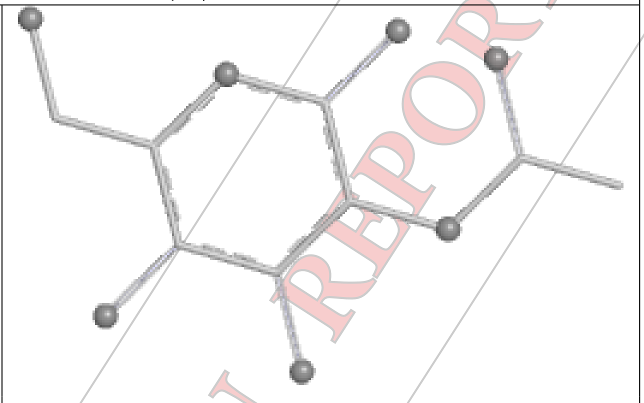

Bond angles

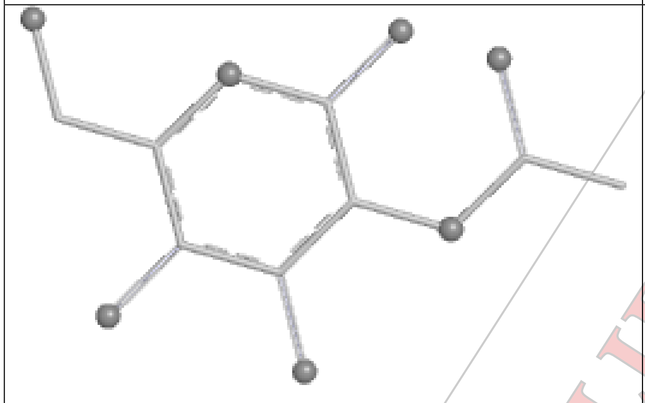

Torsions

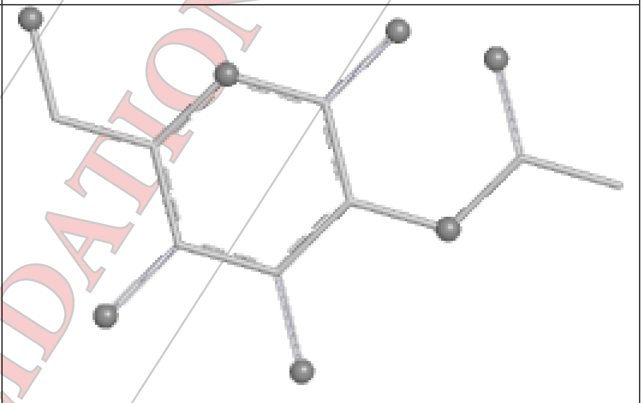

Rings

CONFIDENTIAL VALIDATION REPORT

Ligand NGA BBB 404 (B)	
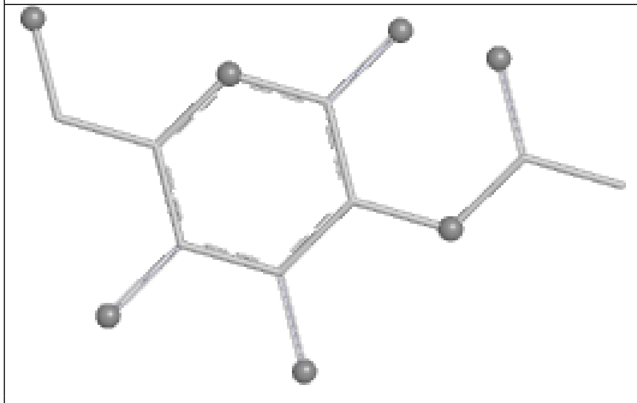 Bond lengths	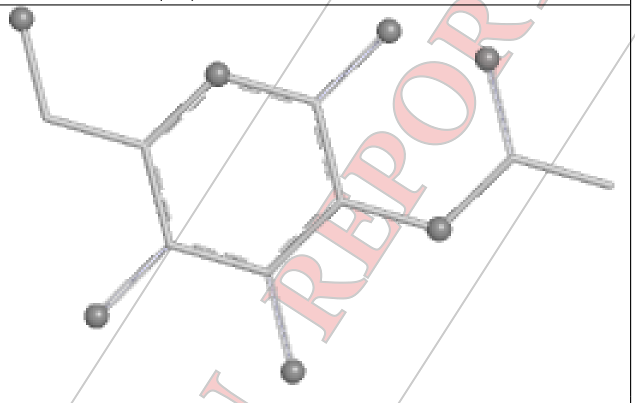 Bond angles
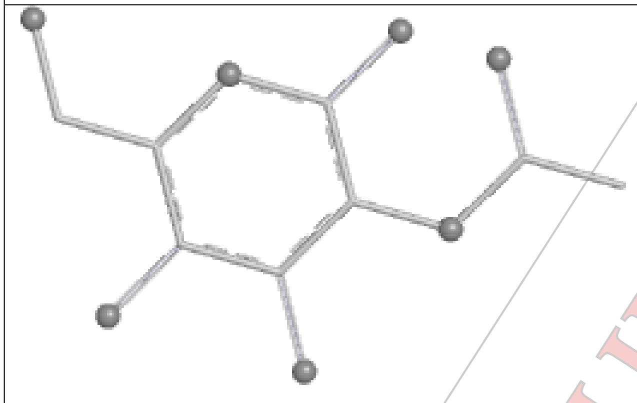 Torsions	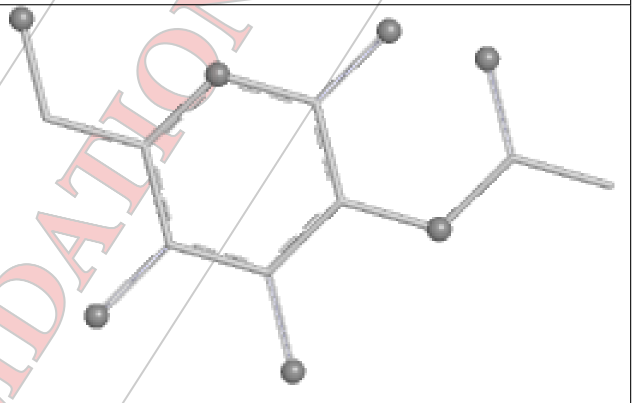 Rings

5.7 Other polymers (i)

There are no such residues in this entry.

5.8 Polymer linkage issues (i)

There are no chain breaks in this entry.

CONFIDENTIAL VALIDATION REPORT

6 Fit of model and data (i)

6.1 Protein, DNA and RNA chains (i)

In the following table, the column labelled '#RSRZ> 2' contains the number (and percentage) of RSRZ outliers, followed by percent RSRZ outliers for the chain as percentile scores relative to all X-ray entries and entries of similar resolution. The OWAB column contains the minimum, median, 95th percentile and maximum values of the occupancy-weighted average B-factor per residue. The column labelled 'Q< 0.9' lists the number of (and percentage) of residues with an average occupancy less than 0.9.

Mol	Chain	Analysed	<RSRZ>	#RSRZ>2			OWAB(Å^2)	Q<0.9
1	AAA	325/329 (98%)	0.08	10 (3%)	49	53	12, 19, 33, 46	0
1	BBB	322/329 (97%)	-0.19	3 (0%)	84	87	12, 17, 28, 37	0
All	All	647/658 (98%)	-0.05	13 (2%)	65	69	12, 18, 31, 46	0

All (13) RSRZ outliers are listed below:

Mol	Chain	Res	Type	RSRZ
1	AAA	3	SER	3.7
1	AAA	173	ALA	3.7
1	AAA	260	THR	3.6
1	AAA	8	ALA	3.1
1	BBB	327	VAL	3.1
1	AAA	4	THR	2.7
1	AAA	203	VAL	2.5
1	BBB	190	ARG	2.5
1	AAA	205	LEU	2.5
1	AAA	174	GLN	2.4
1	AAA	206	GLU	2.2
1	AAA	271	VAL	2.1
1	BBB	278	CYS	2.0

6.2 Non-standard residues in protein, DNA, RNA chains (i)

There are no non-standard protein/DNA/RNA residues in this entry.

6.3 Carbohydrates (i)

There are no monosaccharides in this entry.

CONFIDENTIAL VALIDATION REPORT

6.4 Ligands (i)

In the following table, the Atoms column lists the number of modelled atoms in the group and the number defined in the chemical component dictionary. The B-factors column lists the minimum, median, 95th percentile and maximum values of B factors of atoms in the group. The column labelled 'Q< 0.9' lists the number of atoms with occupancy less than 0.9.

Mol	Type	Chain	Res	Atoms	RSCC	RSR	B-factors(Å^2)	Q<0.9
5	NA	BBB	406	1/1	0.89	0.48	41,41,41,41	0
2	A2G	BBB	403[A]	15/15	0.92	0.10	14,22,30,30	30
2	A2G	BBB	402	15/15	0.95	0.08	15,21,30,31	4
2	A2G	AAA	402[A]	15/15	0.95	0.10	14,19,23,24	30
3	NGA	AAA	404[B]	15/15	0.95	0.10	13,15,23,23	30
3	NGA	BBB	404[B]	15/15	0.95	0.11	19,22,27,27	30
2	A2G	AAA	403	15/15	0.95	0.08	16,21,28,29	4
2	A2G	AAA	401	15/15	0.96	0.08	13,17,23,23	4
2	A2G	BBB	401	15/15	0.97	0.08	12,15,23,23	4
5	NA	BBB	405	1/1	0.98	0.10	23,23,23,23	0
4	CL	AAA	405	1/1	0.99	0.06	19,19,19,19	0

The following is a graphical depiction of the model fit to experimental electron density of all instances of the Ligand of Interest. In addition, ligands with molecular weight > 250 and outliers as shown on the geometry validation Tables will also be included. Each fit is shown from different orientation to approximate a three-dimensional view.

Electron density around A2G BBB 403 (A):

2mF_o-DF_c (at 0.7 rmsd) in gray
mF_o-DF_c (at 3 rmsd) in purple (negative)
and green (positive)

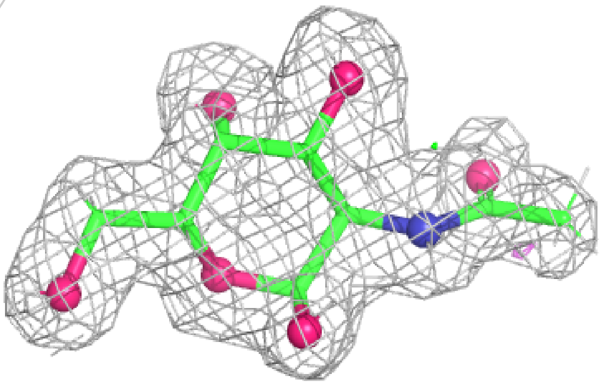

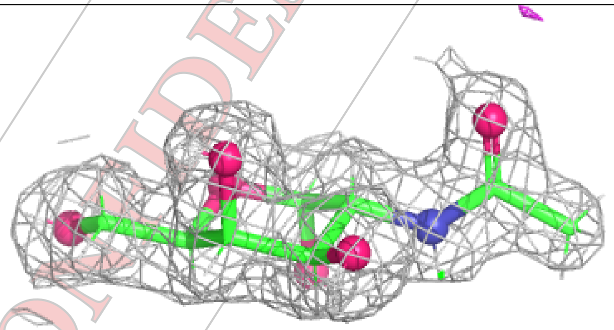

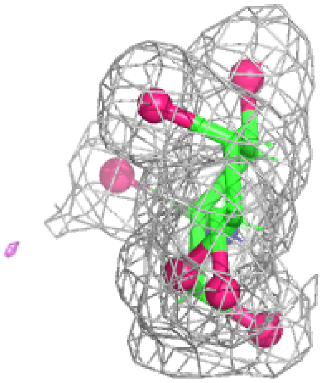

CONFIDENTIAL VALIDATION REPORT

Electron density around A2G BBB 402:

$2mF_o$-DF_c (at 0.7 rmsd) in gray
mF_o-DF_c (at 3 rmsd) in purple (negative)
and green (positive)

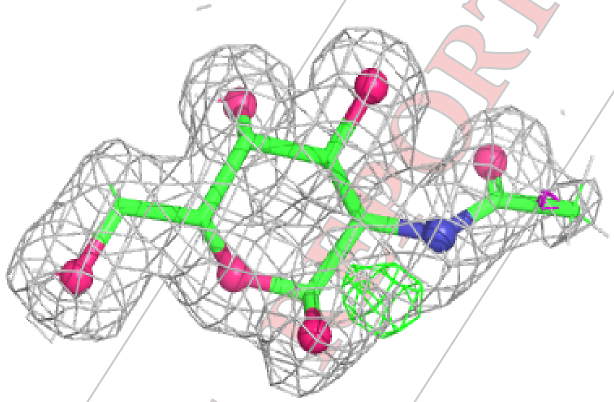

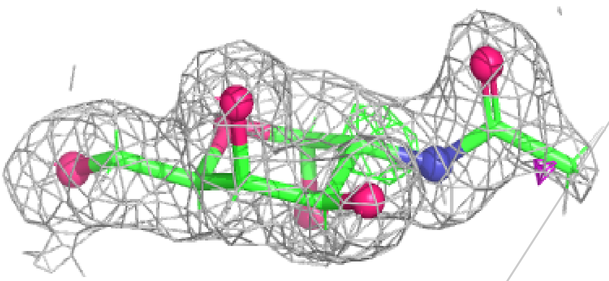

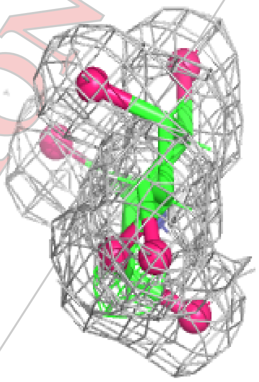

Electron density around A2G AAA 402 (A):

$2mF_o$-DF_c (at 0.7 rmsd) in gray
mF_o-DF_c (at 3 rmsd) in purple (negative)
and green (positive)

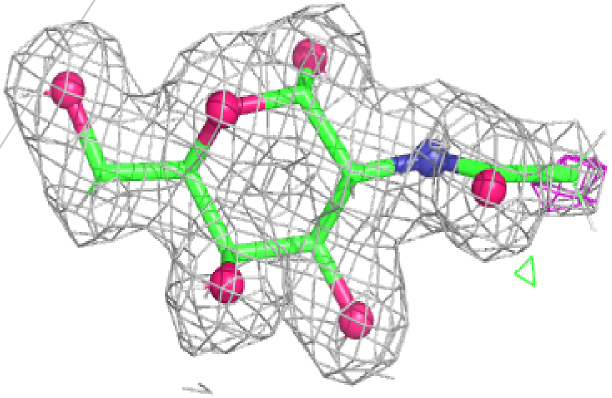

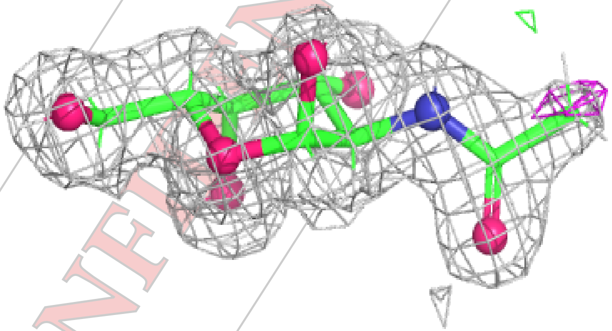

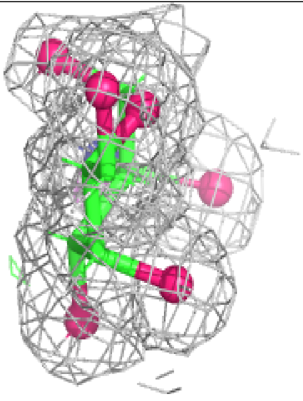

CONFIDENTIAL VALIDATION REPORT

Electron density around NGA AAA 404 (B):

2mF$_o$-DF$_c$ (at 0.7 rmsd) in gray
mF$_o$-DF$_c$ (at 3 rmsd) in purple (negative)
and green (positive)

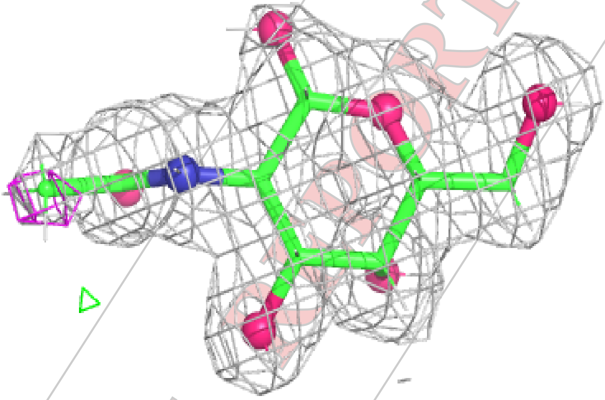

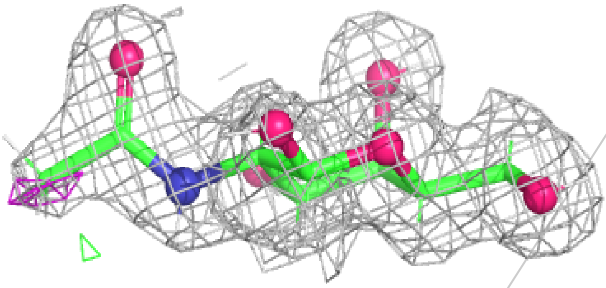

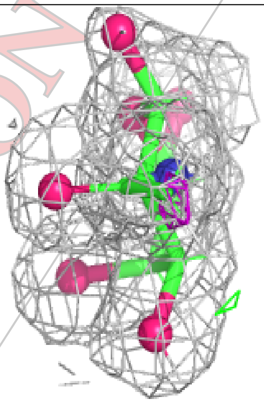

Electron density around NGA BBB 404 (B):

2mF$_o$-DF$_c$ (at 0.7 rmsd) in gray
mF$_o$-DF$_c$ (at 3 rmsd) in purple (negative)
and green (positive)

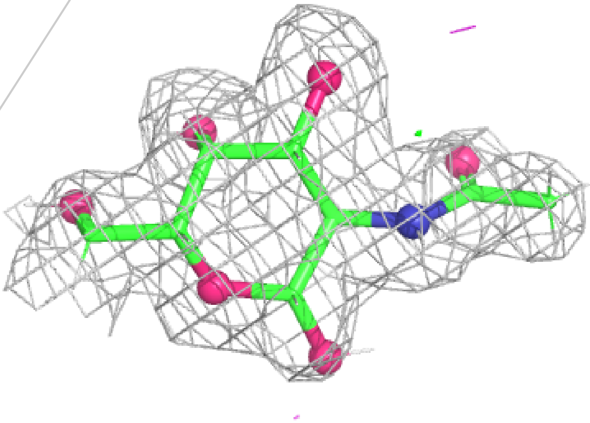

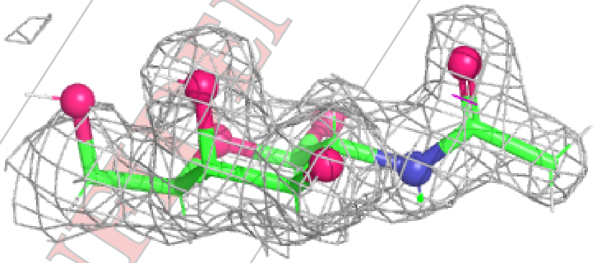

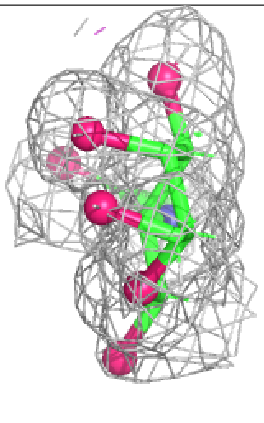

CONFIDENTIAL VALIDATION REPORT

Electron density around A2G AAA 403:

$2mF_o$-DF_c (at 0.7 rmsd) in gray
mF_o-DF_c (at 3 rmsd) in purple (negative)
and green (positive)

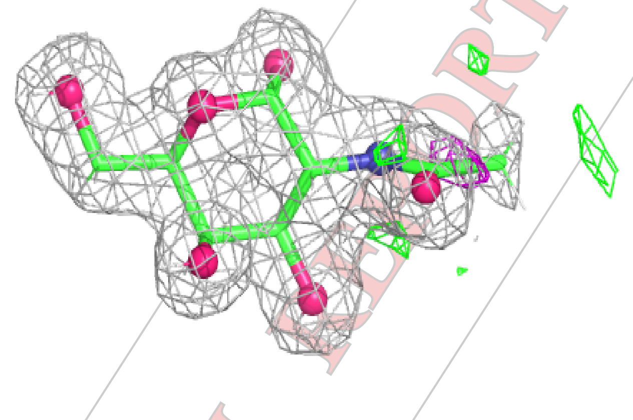

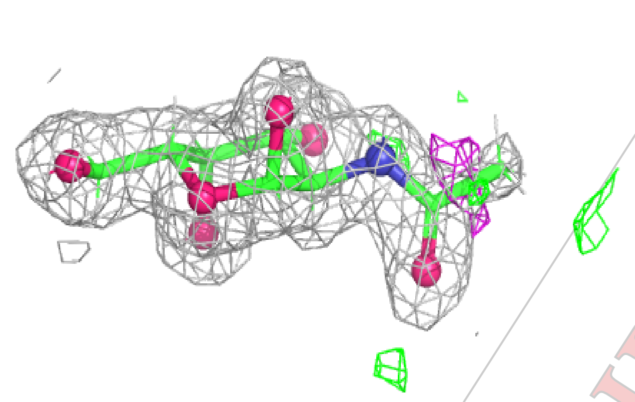

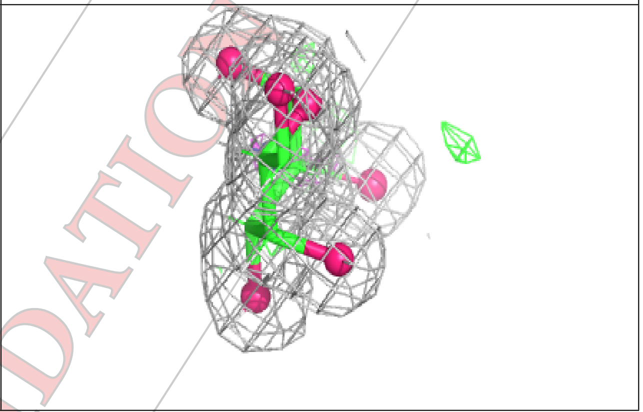

Electron density around A2G AAA 401:

$2mF_o$-DF_c (at 0.7 rmsd) in gray
mF_o-DF_c (at 3 rmsd) in purple (negative)
and green (positive)

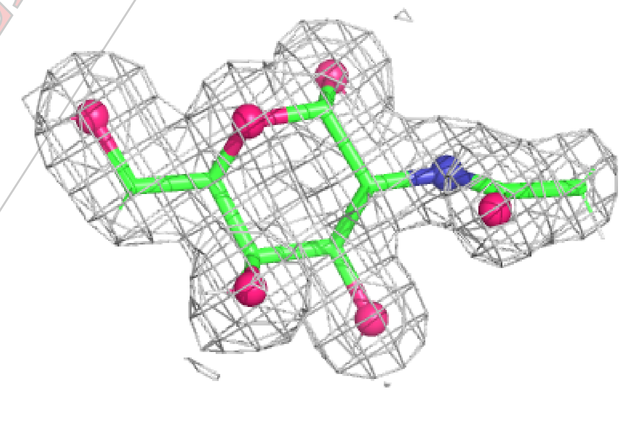

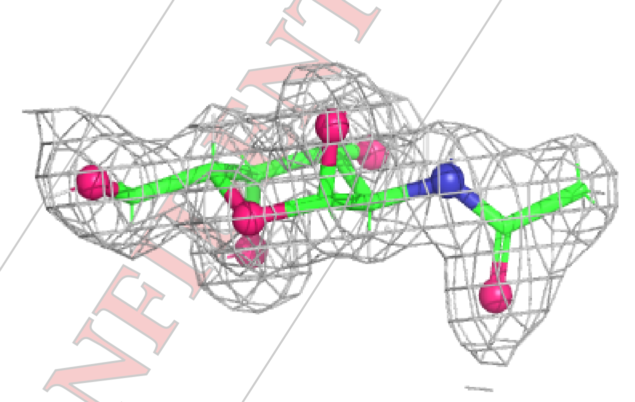

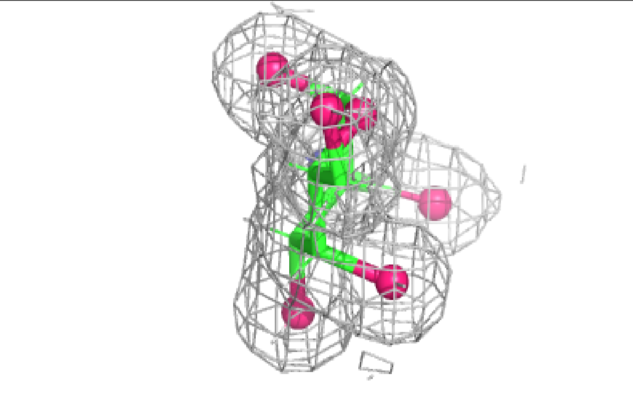

CONFIDENTIAL VALIDATION REPORT

Electron density around A2G BBB 401:

$2mF_o$-DF_c (at 0.7 rmsd) in gray
mF_o-DF_c (at 3 rmsd) in purple (negative)
and green (positive)

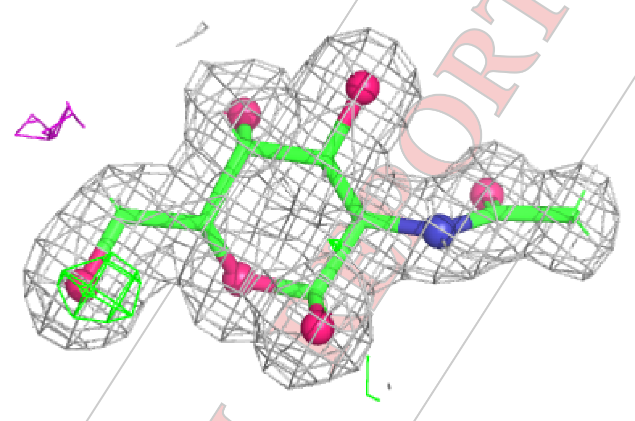

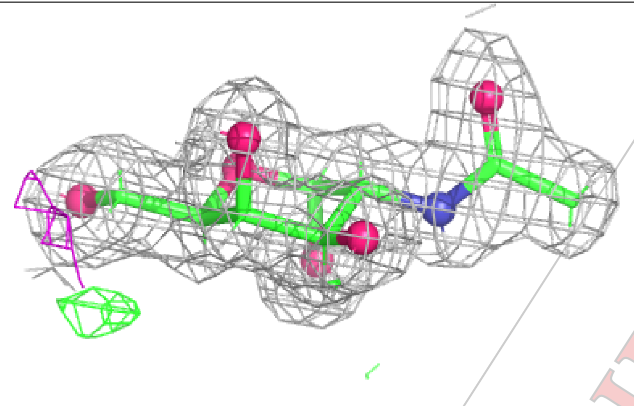

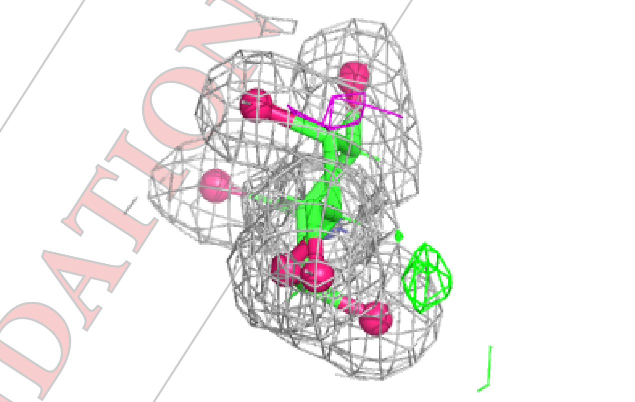

6.5 Other polymers ⓘ

There are no such residues in this entry.

CONFIDENTIAL VALIDATION REPORT
