# Supplementary material for: The choanoflagellate pore-forming lectin SaroL-1 punches holes in cancer cells by targeting the tumor-related glycosphingolipid Gb3
Source: Commun Biol. 2022 Sep 12;5:954. doi: 10.1038/s42003-022-03869-w (PMC9468336; doi:10.1038/s42003-022-03869-w)
Supplement: Supplementary file 7 — validation report 7R55 [file 42003_2022_3869_MOESM7_ESM.pdf]

Full wwPDB X-ray Structure Validation Report (i)

Feb 16, 2022 – 10:58 am GMT

PDB ID	:	7R55
Title	:	B-trefoil lectin from Salpingoeca rosetta in complex with Gb3
Deposited on	:	2022-02-10
Resolution	:	1.84 Å(reported)

This wwPDB validation report is for manuscript review

This is a Full wwPDB X-ray Structure Validation Report.

This report is produced by the wwPDB biocuration pipeline after annotation of the structure.

We welcome your comments at *validation@mail.wwpdb.org*
A user guide is available at
https://www.wwpdb.org/validation/2017/XrayValidationReportHelp
with specific help available everywhere you see the (i) symbol.

The following versions of software and data (see references (i)) were used in the production of this report:

MolProbity	:	4.02b-467
Mogul	:	1.8.4, CSD as541be (2020)
Xtriage (Phenix)	:	1.13
EDS	:	2.26
Percentile statistics	:	20191225.v01 (using entries in the PDB archive December 25th 2019)
Refmac	:	5.8.0267
CCP4	:	7.1.010 (Gargrove)
Ideal geometry (proteins)	:	Engh & Huber (2001)
Ideal geometry (DNA, RNA)	:	Parkinson et al. (1996)
Validation Pipeline (wwPDB-VP)	:	2.26

For Manuscript Review

1 Overall quality at a glance (i)

The following experimental techniques were used to determine the structure:
X-RAY DIFFRACTION

The reported resolution of this entry is 1.84 Å.

Percentile scores (ranging between 0-100) for global validation metrics of the entry are shown in the following graphic. The table shows the number of entries on which the scores are based.

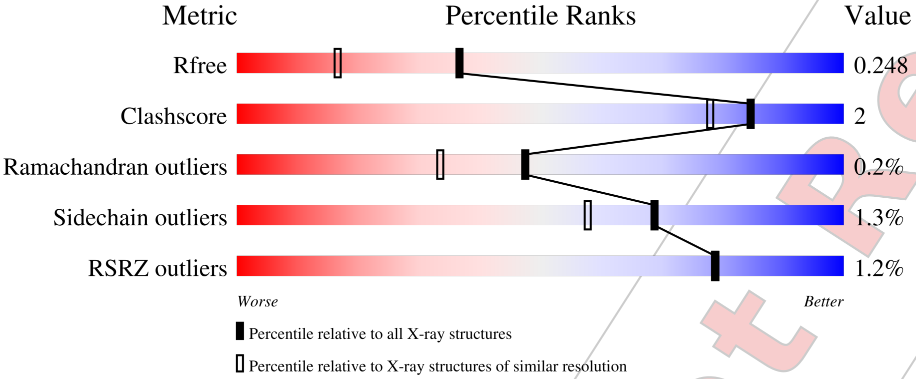

Metric	Whole archive (#Entries)	Similar resolution (#Entries, resolution range(Å))
R_{free}	130704	4003 (1.86-1.82)
Clashscore	141614	4233 (1.86-1.82)
Ramachandran outliers	138981	4185 (1.86-1.82)
Sidechain outliers	138945	4186 (1.86-1.82)
RSRZ outliers	127900	3957 (1.86-1.82)

The table below summarises the geometric issues observed across the polymeric chains and their fit to the electron density. The red, orange, yellow and green segments of the lower bar indicate the fraction of residues that contain outliers for >=3, 2, 1 and 0 types of geometric quality criteria respectively. A grey segment represents the fraction of residues that are not modelled. The numeric value for each fraction is indicated below the corresponding segment, with a dot representing fractions <=5% The upper red bar (where present) indicates the fraction of residues that have poor fit to the electron density. The numeric value is given above the bar.

Mol	Chain	Length	Quality of chain
1	A	329	2% / 91% 7% •
1	B	329	% / 91% 8% •
2	C	3	100%
2	D	3	100%
2	E	3	100%

Continued on next page...

For Manuscript Review

Continued from previous page...

Mol	Chain	Length	Quality of chain
2	F	3	67% 33%

For Manuscript Review

2 Entry composition (i)

There are 4 unique types of molecules in this entry. The entry contains 10902 atoms, of which 5083 are hydrogens and 0 are deuteriums.

In the tables below, the ZeroOcc column contains the number of atoms modelled with zero occupancy, the AltConf column contains the number of residues with at least one atom in alternate conformation and the Trace column contains the number of residues modelled with at most 2 atoms.

- Molecule 1 is a protein called Sarol-1.

Mol	Chain	Residues	Atoms						ZeroOcc	AltConf	Trace
			Total	C	H	N	O	S			
1	A	324	5023	1593	2480	454	488	8	120	9	0
1	B	325	4996	1586	2469	448	485	8	119	6	0

- Molecule 2 is an oligosaccharide called alpha-D-galactopyranose-(1-4)-beta-D-galactopyranose-(1-4)-alpha-D-glucopyranose.

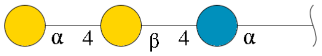

Mol	Chain	Residues	Atoms				ZeroOcc	AltConf	Trace
			Total	C	H	O			
2	C	3	66	18	32	16	11	0	0
2	D	3	66	18	32	16	11	0	0
2	E	3	66	18	32	16	11	0	0
2	F	3	66	18	32	16	11	0	0

- Molecule 3 is 1,2-ETHANEDIOL (three-letter code: EDO) (formula: $C_2H_6O_2$).

For Manuscript Review

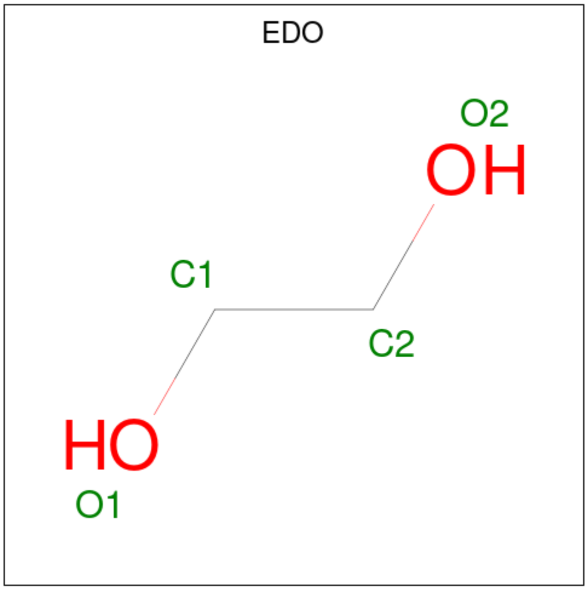

Mol	Chain	Residues	Atoms	ZeroOcc	AltConf
3	B	1	Total C H O<br>10 2 6 2	1	0

- Molecule 4 is water.

Mol	Chain	Residues	Atoms	ZeroOcc	AltConf
4	A	331	Total O<br>333 333	0	2
4	B	273	Total O<br>276 276	0	3

For Manuscript Review

3 Residue-property plots (i)

These plots are drawn for all protein, RNA, DNA and oligosaccharide chains in the entry. The first graphic for a chain summarises the proportions of the various outlier classes displayed in the second graphic. The second graphic shows the sequence view annotated by issues in geometry and electron density. Residues are color-coded according to the number of geometric quality criteria for which they contain at least one outlier: green = 0, yellow = 1, orange = 2 and red = 3 or more. A red dot above a residue indicates a poor fit to the electron density (RSRZ > 2). Stretches of 2 or more consecutive residues without any outlier are shown as a green connector. Residues present in the sample, but not in the model, are shown in grey.

• Molecule 1: Sarol-1

Chain A:

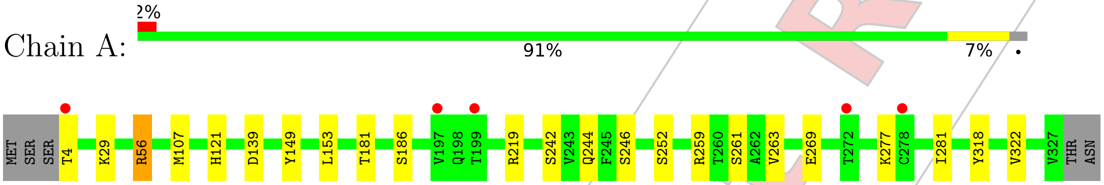

• Molecule 1: Sarol-1

Chain B:

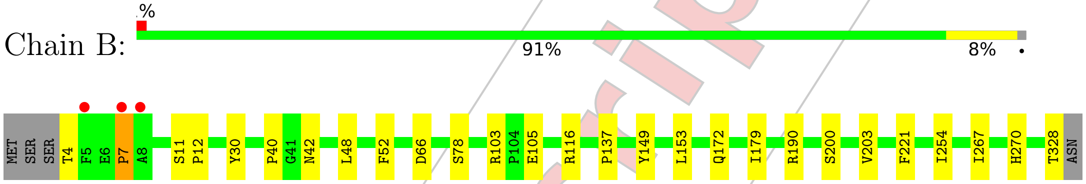

• Molecule 2: alpha-D-galactopyranose-(1-4)-beta-D-galactopyranose-(1-4)-alpha-D-glucopyranos e

Chain C:

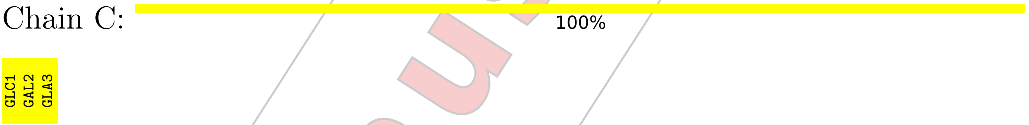

• Molecule 2: alpha-D-galactopyranose-(1-4)-beta-D-galactopyranose-(1-4)-alpha-D-glucopyranos e

Chain D:

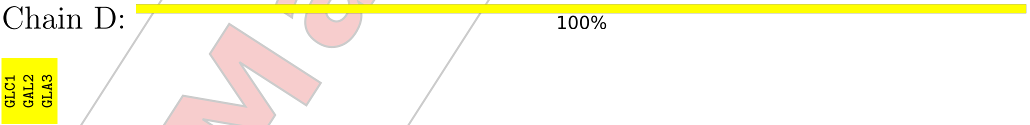

• Molecule 2: alpha-D-galactopyranose-(1-4)-beta-D-galactopyranose-(1-4)-alpha-D-glucopyranos e

Chain E:

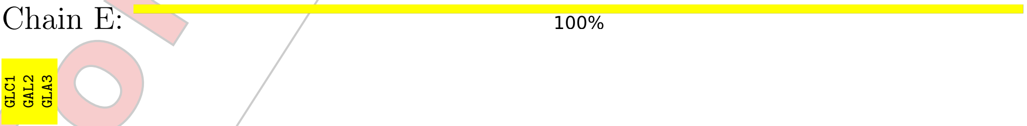

For Manuscript Review

• Molecule 2: alpha-D-galactopyranose-(1-4)-beta-D-galactopyranose-(1-4)-alpha-D-glucopyranose

Chain F: 67% 33%

GLC1 GAL2 GLA3

For Manuscript Review

4 Data and refinement statistics (i)

Property	Value	Source
Space group	P 21 21 21	Depositor
Cell constants<br>a, b, c, α, β, γ	57.34Å 59.00Å 210.31Å<br>90.00° 90.00° 90.00°	Depositor
Resolution (Å)	45.18 – 1.84<br>45.14 – 1.84	Depositor<br>EDS
% Data completeness<br>(in resolution range)	99.8 (45.18-1.84)<br>99.7 (45.14-1.84)	Depositor<br>EDS
R_{merge}	0.09	Depositor
R_{sym}	(Not available)	Depositor
$< I/\sigma(I) >$ [1]	1.54 (at 1.84Å)	Xtriage
Refinement program	REFMAC 5.8.0267, REFMAC 5.8.0267	Depositor
R, R_{free}	0.184 , 0.240<br>0.190 , 0.248	Depositor<br>DCC
R_{free} test set	2979 reflections (4.75%)	wwPDB-VP
Wilson B-factor (Å^2)	23.5	Xtriage
Anisotropy	0.580	Xtriage
Bulk solvent k_{sol}(e/Å^3), B_{sol}(Å^2)	(Not available) , (Not available)	EDS
L-test for twinning[2]	$< \|L\| > = 0.52$, $< L^2 > = 0.35$	Xtriage
Estimated twinning fraction	0.000 for k,h,-l	Xtriage
F_o,F_c correlation	0.96	EDS
Total number of atoms	10902	wwPDB-VP
Average B, all atoms (Å^2)	30.0	wwPDB-VP

Xtriage's analysis on translational NCS is as follows: *The analyses of the Patterson function reveals a significant off-origin peak that is 42.17 % of the origin peak, indicating pseudo-translational symmetry. The chance of finding a peak of this or larger height randomly in a structure without pseudo-translational symmetry is equal to 2.1221e-04. The detected translational NCS is most likely also responsible for the elevated intensity ratio.*

[1] Intensities estimated from amplitudes.
[2] Theoretical values of $< |L| >$, $< L^2 >$ for acentric reflections are 0.5, 0.333 respectively for untwinned datasets, and 0.375, 0.2 for perfectly twinned datasets.

For Manuscript Review

5 Model quality (i)

5.1 Standard geometry (i)

Bond lengths and bond angles in the following residue types are not validated in this section: GAL, GLA, GLC, EDO

The Z score for a bond length (or angle) is the number of standard deviations the observed value is removed from the expected value. A bond length (or angle) with $|Z| > 5$ is considered an outlier worth inspection. RMSZ is the root-mean-square of all Z scores of the bond lengths (or angles).

Mol	Chain	Bond lengths		Bond angles	
		RMSZ	#|Z| >5	RMSZ	#|Z| >5
1	A	0.94	1/2605 (0.0%)	1.05	2/3549 (0.1%)
1	B	0.95	0/2589	1.06	2/3533 (0.1%)
All	All	0.95	1/5194 (0.0%)	1.06	4/7082 (0.1%)

All (1) bond length outliers are listed below:

Mol	Chain	Res	Type	Atoms	Z	Observed(Å)	Ideal(Å)
1	A	318	TYR	C-O	5.14	1.33	1.23

All (4) bond angle outliers are listed below:

Mol	Chain	Res	Type	Atoms	Z	Observed(^{o})	Ideal(^{o})
1	A	56	ARG	NE-CZ-NH1	-7.28	116.66	120.30
1	A	56	ARG	NE-CZ-NH2	6.58	123.59	120.30
1	B	116	ARG	NE-CZ-NH2	-6.11	117.24	120.30
1	B	116	ARG	NE-CZ-NH1	5.33	122.97	120.30

There are no chirality outliers.

There are no planarity outliers.

5.2 Too-close contacts (i)

In the following table, the Non-H and H(model) columns list the number of non-hydrogen atoms and hydrogen atoms in the chain respectively. The H(added) column lists the number of hydrogen atoms added and optimized by MolProbity. The Clashes column lists the number of clashes within the asymmetric unit, whereas Symm-Clashes lists symmetry-related clashes.

Mol	Chain	Non-H	H(model)	H(added)	Clashes	Symm-Clashes
1	A	2543	2480	2424	10	1

Continued on next page...

For Manuscript Review

Continued from previous page...

Mol	Chain	Non-H	H(model)	H(added)	Clashes	Symm-Clashes
1	B	2527	2469	2415	13	1
2	C	34	32	30	0	0
2	D	34	32	30	0	0
2	E	34	32	30	0	0
2	F	34	32	30	1	0
3	B	4	6	6	0	0
4	A	333	0	0	1	0
4	B	276	0	0	4	0
All	All	5819	5083	4965	23	1

The all-atom clashscore is defined as the number of clashes found per 1000 atoms (including hydrogen atoms). The all-atom clashscore for this structure is 2.

All (23) close contacts within the same asymmetric unit are listed below, sorted by their clash magnitude.

Atom-1	Atom-2	Interatomic distance (Å)	Clash overlap (Å)
1:A:269:GLU:HG3	1:A:322:VAL:HG11	1.64	0.77
1:B:203[A]:VAL:HG22	1:B:267:ILE:HB	1.72	0.71
1:B:221:PHE:CD2	1:B:254[B]:ILE:HD11	2.29	0.68
1:B:105:GLU:OE2	4:B:501:HOH:O	2.12	0.68
1:A:29:LYS:HE3	1:A:139:ASP:OD1	1.95	0.67
1:A:219:ARG:O	1:A:252[A]:SER:OG	2.23	0.57
1:B:48:LEU:HD12	1:B:137:PRO:HG2	1.92	0.52
1:B:42:ASN:HD22	2:F:1:GLC:H3	1.77	0.50
1:B:66:ASP:HA	4:B:503:HOH:O	2.11	0.50
1:B:12:PRO:HG2	4:B:618:HOH:O	2.12	0.48
1:B:7:PRO:O	1:B:11:SER:HB3	2.13	0.48
1:A:149:TYR:HA	1:A:153:LEU:HD12	1.96	0.47
1:B:190:ARG:NH2	4:B:518:HOH:O	2.47	0.47
1:A:107:MET:O	1:A:121:HIS:HA	2.19	0.43
1:B:149:TYR:HA	1:B:153:LEU:HD12	2.01	0.43
1:A:186:SER:HB3	1:A:281:ILE:HG12	2.00	0.43
1:B:40:PRO:HD2	1:B:78:SER:HB3	2.00	0.42
1:B:200:SER:HB3	1:B:270:HIS:CD2	2.55	0.42
1:A:244:GLN:HB3	1:A:259:ARG:HD3	2.01	0.42
1:A:261:SER:OG	1:A:263:VAL:HG22	2.20	0.42
1:A:4:THR:N	4:A:420:HOH:O	2.54	0.41
1:B:30:TYR:CE1	1:B:52:PHE:HB2	2.56	0.41
1:A:277:LYS:HA	1:A:277:LYS:HD3	1.96	0.40

All (1) symmetry-related close contacts are listed below. The label for Atom-2 includes the sym-

For Manuscript Review

metry operator and encoded unit-cell translations to be applied.

Atom-1	Atom-2	Interatomic distance (Å)	Clash overlap (Å)
1:A:181:THR:HG1	1:B:179:ILE:H[1_565]	1.33	0.27

5.3 Torsion angles (i)

5.3.1 Protein backbone (i)

In the following table, the Percentiles column shows the percent Ramachandran outliers of the chain as a percentile score with respect to all X-ray entries followed by that with respect to entries of similar resolution.

The Analysed column shows the number of residues for which the backbone conformation was analysed, and the total number of residues.

Mol	Chain	Analysed	Favoured	Allowed	Outliers	Percentiles	
1	A	331/329 (101%)	324 (98%)	7 (2%)	0	100	100
1	B	329/329 (100%)	317 (96%)	11 (3%)	1 (0%)	41	27
All	All	660/658 (100%)	641 (97%)	18 (3%)	1 (0%)	47	33

All (1) Ramachandran outliers are listed below:

Mol	Chain	Res	Type
1	B	7	PRO

5.3.2 Protein sidechains (i)

In the following table, the Percentiles column shows the percent sidechain outliers of the chain as a percentile score with respect to all X-ray entries followed by that with respect to entries of similar resolution.

The Analysed column shows the number of residues for which the sidechain conformation was analysed, and the total number of residues.

Mol	Chain	Analysed	Rotameric	Outliers	Percentiles	
1	A	276/289 (96%)	272 (99%)	4 (1%)	67	55
1	B	275/289 (95%)	271 (98%)	4 (2%)	65	52
All	All	551/578 (95%)	543 (98%)	8 (2%)	69	52

All (8) residues with a non-rotameric sidechain are listed below:

For Manuscript Review

Mol	Chain	Res	Type
1	A	56	ARG
1	A	242[A]	SER
1	A	242[B]	SER
1	A	246	SER
1	B	4	THR
1	B	103	ARG
1	B	172	GLN
1	B	328	THR

Sometimes sidechains can be flipped to improve hydrogen bonding and reduce clashes. All (1) such sidechains are listed below:

Mol	Chain	Res	Type
1	B	195	GLN

5.3.3 RNA (i)

There are no RNA molecules in this entry.

5.4 Non-standard residues in protein, DNA, RNA chains (i)

There are no non-standard protein/DNA/RNA residues in this entry.

5.5 Carbohydrates (i)

12 monosaccharides are modelled in this entry.

In the following table, the Counts columns list the number of bonds (or angles) for which Mogul statistics could be retrieved, the number of bonds (or angles) that are observed in the model and the number of bonds (or angles) that are defined in the Chemical Component Dictionary. The Link column lists molecule types, if any, to which the group is linked. The Z score for a bond length (or angle) is the number of standard deviations the observed value is removed from the expected value. A bond length (or angle) with $|Z| > 2$ is considered an outlier worth inspection. RMSZ is the root-mean-square of all Z scores of the bond lengths (or angles).

Mol	Type	Chain	Res	Link	Bond lengths			Bond angles		
					Counts	RMSZ	$\#\|Z\| > 2$	Counts	RMSZ	$\#\|Z\| > 2$
2	GLC	C	1	2	12,12,12	0.76	0	17,17,17	1.14	2 (11%)
2	GAL	C	2	2	11,11,12	0.83	1 (9%)	15,15,17	1.60	2 (13%)
2	GLA	C	3	2	11,11,12	1.19	2 (18%)	15,15,17	1.16	0
2	GLC	D	1	2	12,12,12	1.63	4 (33%)	17,17,17	3.05	10 (58%)

For Manuscript Review

Mol	Type	Chain	Res	Link	Bond lengths			Bond angles		
					Counts	RMSZ	#|Z| > 2	Counts	RMSZ	#|Z| > 2
2	GAL	D	2	2	11,11,12	1.04	1 (9%)	15,15,17	1.92	6 (40%)
2	GLA	D	3	2	11,11,12	0.98	0	15,15,17	1.52	3 (20%)
2	GLC	E	1	2	12,12,12	1.24	2 (16%)	17,17,17	1.63	4 (23%)
2	GAL	E	2	2	11,11,12	0.90	0	15,15,17	1.58	3 (20%)
2	GLA	E	3	2	11,11,12	1.15	1 (9%)	15,15,17	1.05	0
2	GLC	F	1	2	12,12,12	1.55	4 (33%)	17,17,17	3.08	11 (64%)
2	GAL	F	2	2	11,11,12	1.19	1 (9%)	15,15,17	2.28	10 (66%)
2	GLA	F	3	2	11,11,12	1.04	1 (9%)	15,15,17	1.54	2 (13%)

In the following table, the Chirals column lists the number of chiral outliers, the number of chiral centers analysed, the number of these observed in the model and the number defined in the Chemical Component Dictionary. Similar counts are reported in the Torsion and Rings columns. '-' means no outliers of that kind were identified.

Mol	Type	Chain	Res	Link	Chirals	Torsions	Rings
2	GLC	C	1	2	-	2/2/22/22	0/1/1/1
2	GAL	C	2	2	-	0/2/19/22	0/1/1/1
2	GLA	C	3	2	-	0/2/19/22	0/1/1/1
2	GLC	D	1	2	-	2/2/22/22	0/1/1/1
2	GAL	D	2	2	-	1/2/19/22	0/1/1/1
2	GLA	D	3	2	-	0/2/19/22	0/1/1/1
2	GLC	E	1	2	-	2/2/22/22	0/1/1/1
2	GAL	E	2	2	-	0/2/19/22	0/1/1/1
2	GLA	E	3	2	-	0/2/19/22	0/1/1/1
2	GLC	F	1	2	-	2/2/22/22	0/1/1/1
2	GAL	F	2	2	-	0/2/19/22	0/1/1/1
2	GLA	F	3	2	-	0/2/19/22	0/1/1/1

All (17) bond length outliers are listed below:

Mol	Chain	Res	Type	Atoms	Z	Observed(Å)	Ideal(Å)
2	D	1	GLC	O1-C1	3.07	1.49	1.39
2	F	1	GLC	O4-C4	2.59	1.49	1.43
2	D	1	GLC	O4-C4	2.43	1.48	1.43
2	F	2	GAL	O5-C5	2.42	1.48	1.43
2	C	3	GLA	C2-C3	2.42	1.56	1.52
2	E	1	GLC	O5-C1	2.30	1.48	1.42
2	E	1	GLC	C3-C2	2.24	1.58	1.52
2	E	3	GLA	C2-C3	-2.24	1.49	1.52

Continued on next page...

For Manuscript Review

Continued from previous page...

Mol	Chain	Res	Type	Atoms	Z	Observed(Å)	Ideal(Å)
2	F	1	GLC	C4-C5	-2.23	1.48	1.53
2	F	1	GLC	O1-C1	2.21	1.46	1.39
2	C	2	GAL	O2-C2	-2.16	1.38	1.43
2	D	1	GLC	O2-C2	2.15	1.48	1.43
2	F	1	GLC	C3-C2	2.15	1.57	1.52
2	D	1	GLC	C1-C2	-2.14	1.47	1.52
2	F	3	GLA	O3-C3	-2.05	1.38	1.43
2	D	2	GAL	O5-C5	2.04	1.47	1.43
2	C	3	GLA	O5-C5	-2.02	1.39	1.43

All (53) bond angle outliers are listed below:

Mol	Chain	Res	Type	Atoms	Z	Observed(°)	Ideal(°)
2	F	1	GLC	O3-C3-C2	-6.89	94.42	110.35
2	D	1	GLC	O2-C2-C1	-5.67	96.01	109.16
2	D	1	GLC	O5-C5-C4	5.15	119.05	109.69
2	F	1	GLC	O4-C4-C3	4.75	121.34	110.35
2	D	1	GLC	O5-C1-C2	4.59	118.48	110.28
2	F	1	GLC	C3-C4-C5	-4.57	102.09	110.24
2	D	1	GLC	O1-C1-C2	-4.27	97.01	109.03
2	F	3	GLA	C1-O5-C5	-4.15	106.57	112.19
2	F	1	GLC	O5-C1-C2	-4.08	103.01	110.28
2	C	2	GAL	O6-C6-C5	-4.03	97.47	111.29
2	F	2	GAL	O2-C2-C1	-3.74	101.51	109.15
2	E	2	GAL	O6-C6-C5	-3.66	98.74	111.29
2	D	2	GAL	C1-O5-C5	-3.58	107.35	112.19
2	F	1	GLC	O6-C6-C5	-3.40	99.62	111.29
2	D	1	GLC	O3-C3-C2	-3.39	102.51	110.35
2	F	1	GLC	O2-C2-C3	3.22	117.79	110.35
2	D	3	GLA	C3-C4-C5	-3.20	104.53	110.24
2	F	2	GAL	O4-C4-C3	3.10	117.51	110.35
2	F	1	GLC	C1-O5-C5	2.97	119.27	113.66
2	E	1	GLC	C6-C5-C4	2.96	119.93	113.00
2	D	1	GLC	O6-C6-C5	-2.94	101.19	111.29
2	D	1	GLC	C4-C3-C2	-2.90	105.76	110.82
2	F	2	GAL	C1-C2-C3	2.89	113.22	109.67
2	C	1	GLC	O3-C3-C2	-2.86	103.73	110.35
2	F	2	GAL	C3-C4-C5	-2.85	105.16	110.24
2	F	1	GLC	O5-C5-C6	2.68	113.10	106.44
2	C	2	GAL	O4-C4-C5	-2.67	102.67	109.30
2	F	2	GAL	C1-O5-C5	-2.63	108.62	112.19
2	D	2	GAL	C3-C4-C5	-2.57	105.66	110.24

Continued on next page...

For Manuscript Review

Continued from previous page...

Mol	Chain	Res	Type	Atoms	Z	Observed(°)	Ideal(°)
2	F	3	GLA	O4-C4-C5	2.47	115.43	109.30
2	E	1	GLC	O2-C2-C1	-2.46	103.45	109.16
2	D	2	GAL	C1-C2-C3	2.43	112.65	109.67
2	F	2	GAL	C6-C5-C4	-2.43	107.32	113.00
2	D	1	GLC	O1-C1-O5	2.40	117.60	110.38
2	D	1	GLC	O4-C4-C3	2.40	115.91	110.35
2	F	2	GAL	O6-C6-C5	-2.33	103.28	111.29
2	D	2	GAL	O5-C1-C2	2.29	114.30	110.77
2	E	2	GAL	O4-C4-C3	2.25	115.54	110.35
2	E	1	GLC	O5-C1-C2	2.22	114.25	110.28
2	F	2	GAL	O3-C3-C2	2.21	114.22	109.99
2	F	2	GAL	O3-C3-C4	-2.20	105.26	110.35
2	D	3	GLA	O5-C5-C6	-2.19	103.77	107.20
2	D	1	GLC	O2-C2-C3	2.15	115.31	110.35
2	D	3	GLA	O3-C3-C2	2.14	114.08	109.99
2	E	2	GAL	O4-C4-C5	-2.12	104.02	109.30
2	F	2	GAL	O5-C5-C6	2.12	110.53	107.20
2	C	1	GLC	O6-C6-C5	-2.11	104.05	111.29
2	D	2	GAL	C6-C5-C4	-2.09	108.11	113.00
2	F	1	GLC	O2-C2-C1	-2.08	104.33	109.16
2	E	1	GLC	C1-O5-C5	-2.07	109.77	113.66
2	D	2	GAL	O5-C5-C6	-2.05	103.99	107.20
2	F	1	GLC	O5-C5-C4	2.03	113.37	109.69
2	F	1	GLC	O1-C1-O5	2.02	116.44	110.38

There are no chirality outliers.

All (9) torsion outliers are listed below:

Mol	Chain	Res	Type	Atoms
2	F	1	GLC	O5-C5-C6-O6
2	E	1	GLC	C4-C5-C6-O6
2	D	1	GLC	C4-C5-C6-O6
2	E	1	GLC	O5-C5-C6-O6
2	F	1	GLC	C4-C5-C6-O6
2	D	1	GLC	O5-C5-C6-O6
2	C	1	GLC	C4-C5-C6-O6
2	D	2	GAL	O5-C5-C6-O6
2	C	1	GLC	O5-C5-C6-O6

There are no ring outliers.

1 monomer is involved in 1 short contact:

For Manuscript Review

Mol	Chain	Res	Type	Clashes	Symm-Clashes
2	F	1	GLC	1	0

The following is a two-dimensional graphical depiction of Mogul quality analysis of bond lengths, bond angles, torsion angles, and ring geometry for oligosaccharide.

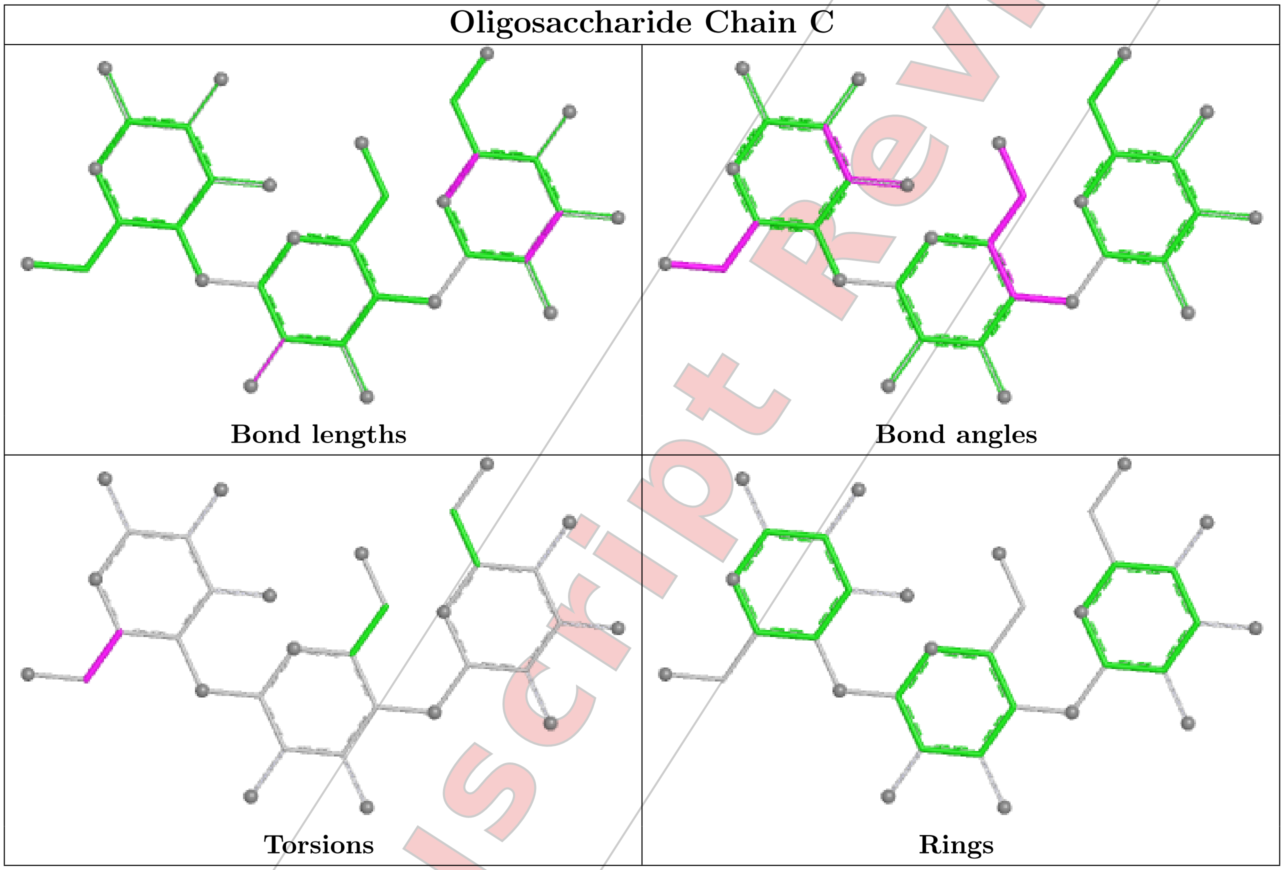

For Manuscript Review

Oligosaccharide Chain D

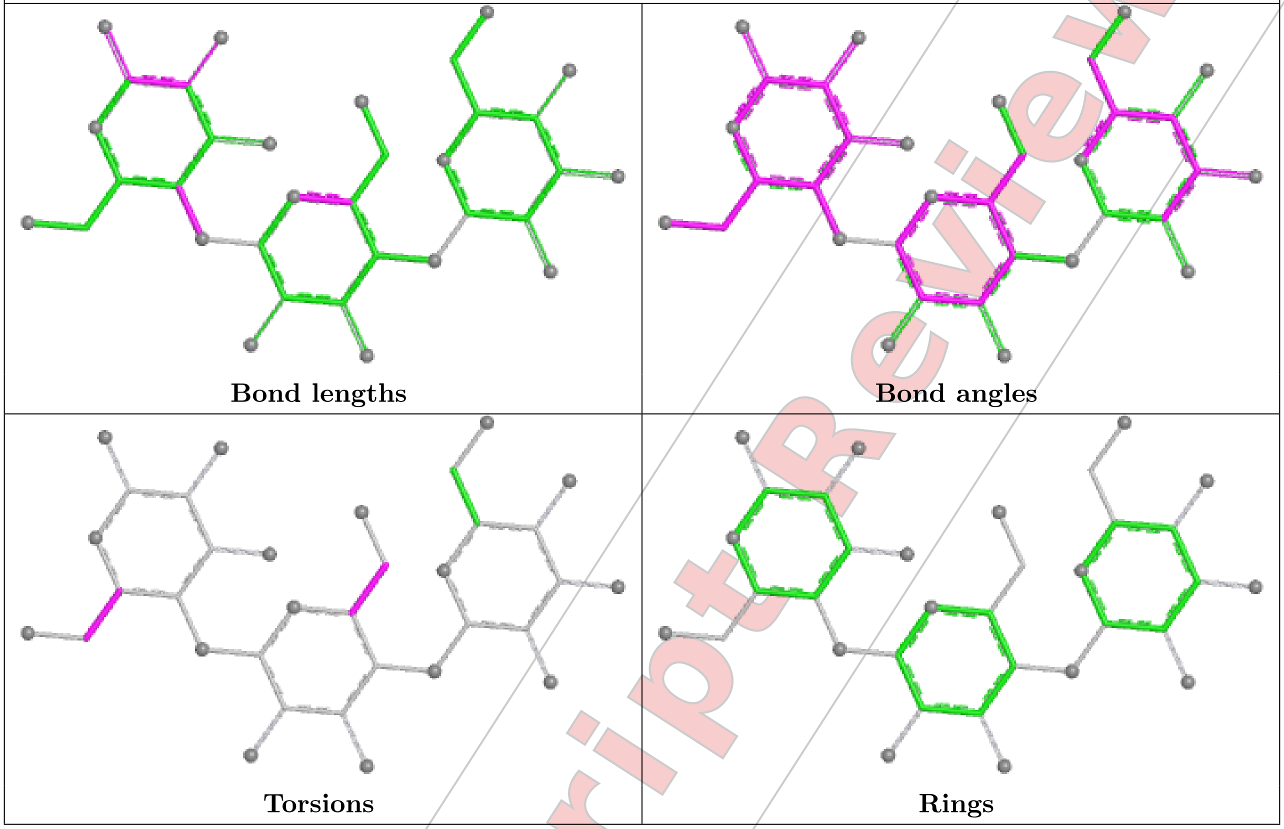

Oligosaccharide Chain E

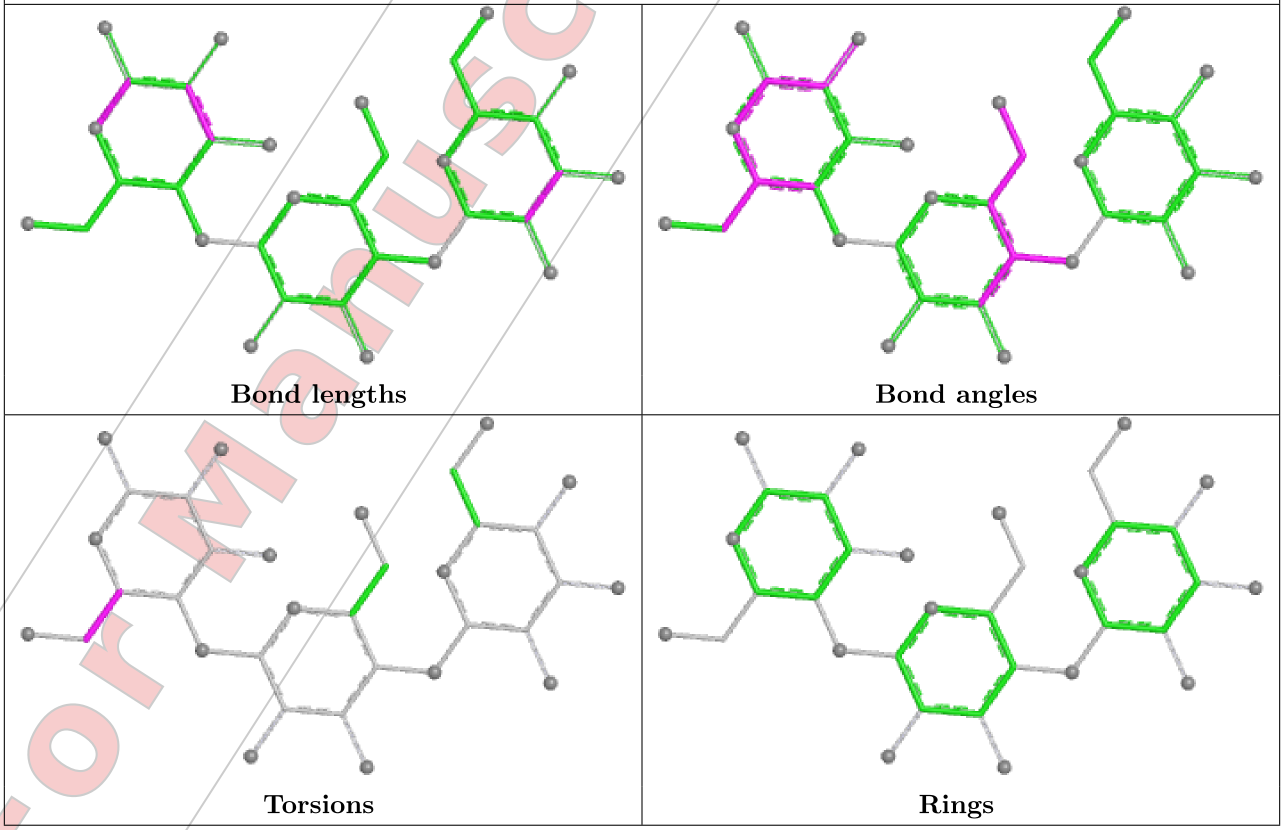

For Manuscript Review

Oligosaccharide Chain F

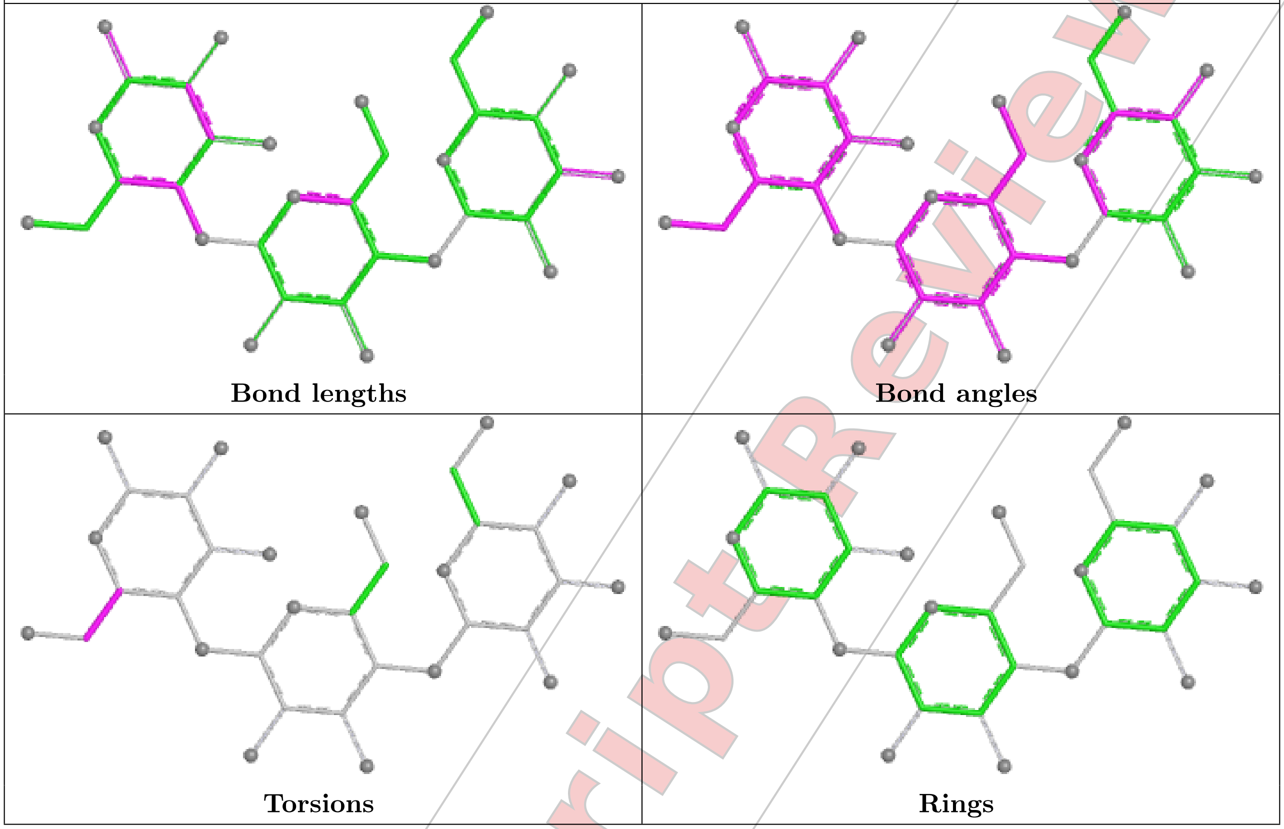

5.6 Ligand geometry (i)

1 ligand is modelled in this entry.

In the following table, the Counts columns list the number of bonds (or angles) for which Mogul statistics could be retrieved, the number of bonds (or angles) that are observed in the model and the number of bonds (or angles) that are defined in the Chemical Component Dictionary. The Link column lists molecule types, if any, to which the group is linked. The Z score for a bond length (or angle) is the number of standard deviations the observed value is removed from the expected value. A bond length (or angle) with $|Z| > 2$ is considered an outlier worth inspection. RMSZ is the root-mean-square of all Z scores of the bond lengths (or angles).

Mol	Type	Chain	Res	Link	Bond lengths			Bond angles		
					Counts	RMSZ	$\#|Z| > 2$	Counts	RMSZ	$\#|Z| > 2$
3	EDO	B	401	-	3,3,3	1.28	1 (33%)	2,2,2	0.47	0

In the following table, the Chirals column lists the number of chiral outliers, the number of chiral centers analysed, the number of these observed in the model and the number defined in the Chemical Component Dictionary. Similar counts are reported in the Torsion and Rings columns. '-' means no outliers of that kind were identified.

For Manuscript Review

Mol	Type	Chain	Res	Link	Chirals	Torsions	Rings
3	EDO	B	401	-	-	0/1/1/1	-

All (1) bond length outliers are listed below:

Mol	Chain	Res	Type	Atoms	Z	Observed(Å)	Ideal(Å)
3	B	401	EDO	C2-C1	-2.17	1.33	1.48

There are no bond angle outliers.

There are no chirality outliers.

There are no torsion outliers.

There are no ring outliers.

No monomer is involved in short contacts.

5.7 Other polymers (i)

There are no such residues in this entry.

5.8 Polymer linkage issues (i)

There are no chain breaks in this entry.

For Manuscript Review

6 Fit of model and data (i)

6.1 Protein, DNA and RNA chains (i)

In the following table, the column labelled '#RSRZ> 2' contains the number (and percentage) of RSRZ outliers, followed by percent RSRZ outliers for the chain as percentile scores relative to all X-ray entries and entries of similar resolution. The OWAB column contains the minimum, median, 95th percentile and maximum values of the occupancy-weighted average B-factor per residue. The column labelled 'Q< 0.9' lists the number of (and percentage) of residues with an average occupancy less than 0.9.

Mol	Chain	Analysed	<RSRZ>	#RSRZ>2			OWAB(Å^2)	Q<0.9
1	A	324/329 (98%)	-0.19	5 (1%)	73	73	18, 25, 48, 60	0
1	B	325/329 (98%)	-0.26	3 (0%)	84	84	19, 29, 49, 71	0
All	All	649/658 (98%)	-0.23	8 (1%)	79	79	18, 27, 49, 71	0

All (8) RSRZ outliers are listed below:

Mol	Chain	Res	Type	RSRZ
1	B	7	PRO	2.7
1	B	8	ALA	2.5
1	A	272	THR	2.5
1	A	197	VAL	2.3
1	B	5	PHE	2.3
1	A	199	THR	2.1
1	A	4	THR	2.0
1	A	278	CYS	2.0

6.2 Non-standard residues in protein, DNA, RNA chains (i)

There are no non-standard protein/DNA/RNA residues in this entry.

6.3 Carbohydrates (i)

In the following table, the Atoms column lists the number of modelled atoms in the group and the number defined in the chemical component dictionary. The B-factors column lists the minimum, median, 95th percentile and maximum values of B factors of atoms in the group. The column labelled 'Q< 0.9' lists the number of atoms with occupancy less than 0.9.

Mol	Type	Chain	Res	Atoms	RSCC	RSR	B-factors(Å^2)	Q<0.9
2	GLC	F	1	12/12	0.86	0.14	30,45,57,60	4

Continued on next page...

For Manuscript Review

Continued from previous page...

Mol	Type	Chain	Res	Atoms	RSCC	RSR	B-factors(Å^2)	Q<0.9
2	GLC	E	1	12/12	0.88	0.12	30,44,54,62	4
2	GLC	D	1	12/12	0.88	0.14	30,42,50,53	4
2	GLC	C	1	12/12	0.93	0.13	30,42,51,62	4
2	GAL	D	2	11/12	0.94	0.13	24,28,34,42	3
2	GAL	F	2	11/12	0.95	0.13	22,28,33,39	3
2	GAL	C	2	11/12	0.96	0.09	22,31,41,54	3
2	GAL	E	2	11/12	0.96	0.07	23,29,31,35	3
2	GLA	E	3	11/12	0.98	0.07	18,22,30,30	4
2	GLA	C	3	11/12	0.98	0.08	19,23,30,30	4
2	GLA	D	3	11/12	0.98	0.11	22,26,30,31	4
2	GLA	F	3	11/12	0.98	0.13	20,23,30,30	4

The following is a graphical depiction of the model fit to experimental electron density for oligosaccharide. Each fit is shown from different orientation to approximate a three-dimensional view.

Electron density around Chain C:

2mF$_o$-DF$_c$ (at 0.7 rmsd) in gray
mF$_o$-DF$_c$ (at 3 rmsd) in purple (negative) and green (positive)

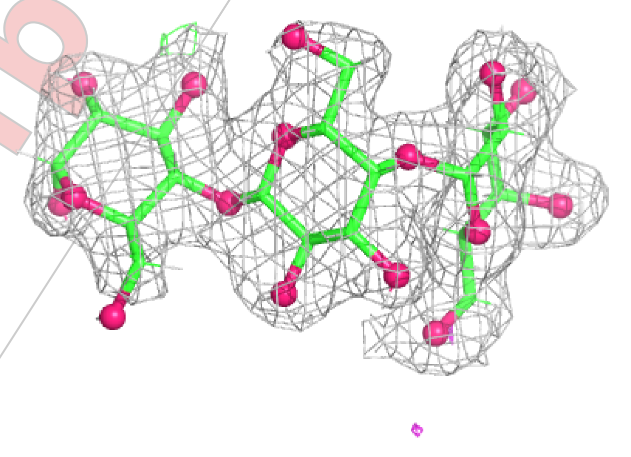

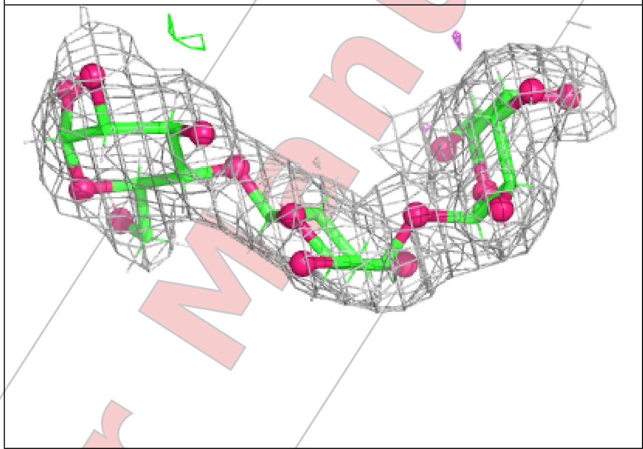

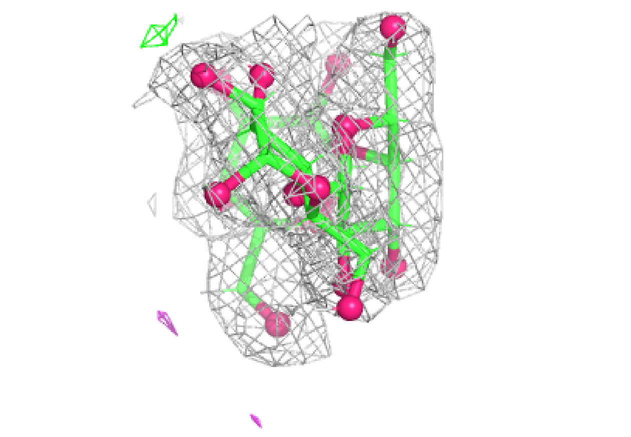

For Manuscript Review

Electron density around Chain D:

2mF$_o$-DF$_c$ (at 0.7 rmsd) in gray
mF$_o$-DF$_c$ (at 3 rmsd) in purple (negative)
and green (positive)

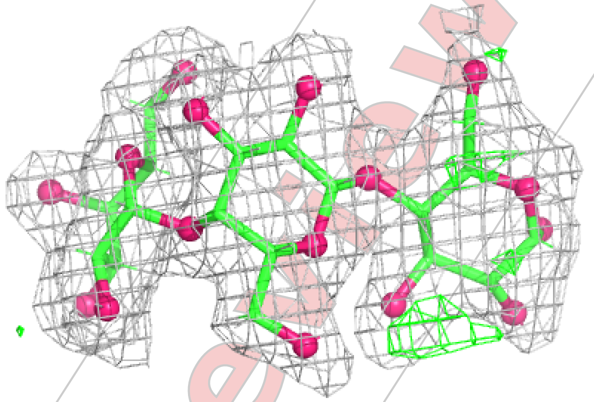

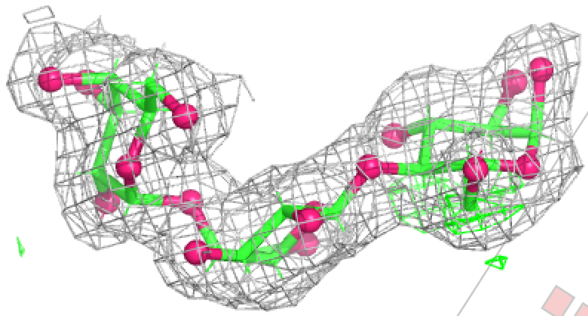

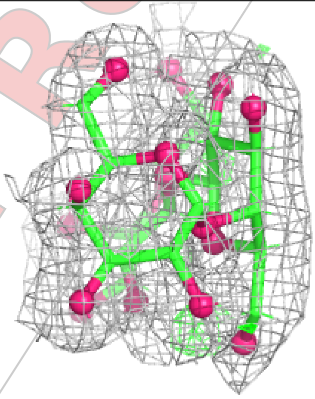

Electron density around Chain E:

2mF$_o$-DF$_c$ (at 0.7 rmsd) in gray
mF$_o$-DF$_c$ (at 3 rmsd) in purple (negative)
and green (positive)

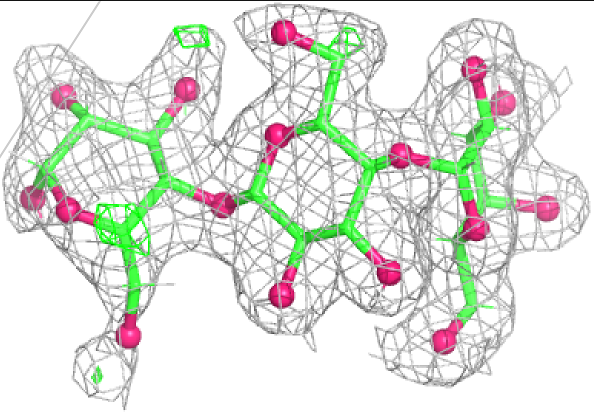

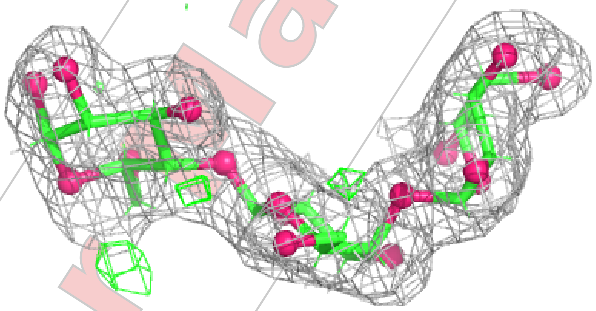

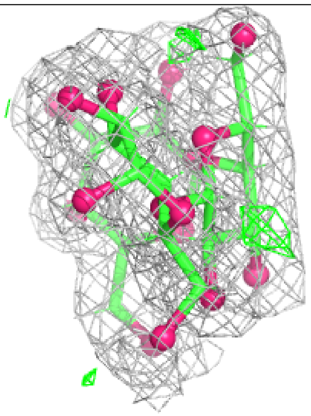

For Manuscript Review

Electron density around Chain F:

$2mF_o$-DF_c (at 0.7 rmsd) in gray
mF_o-DF_c (at 3 rmsd) in purple (negative)
and green (positive)

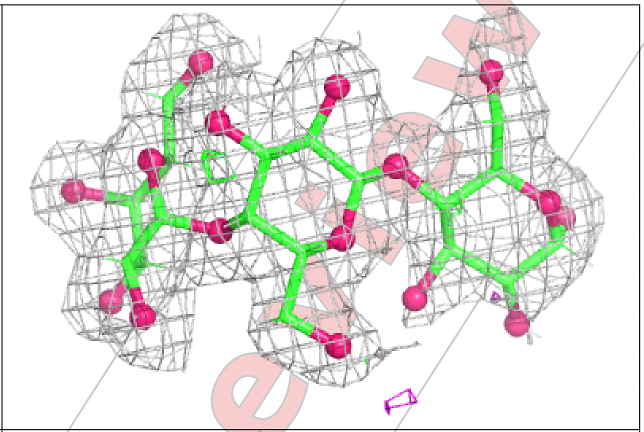

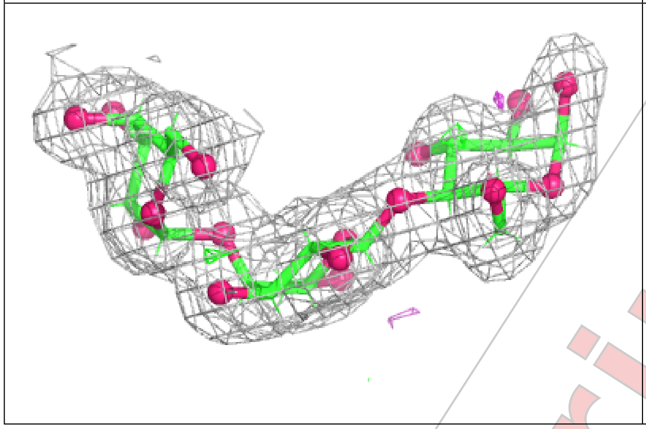

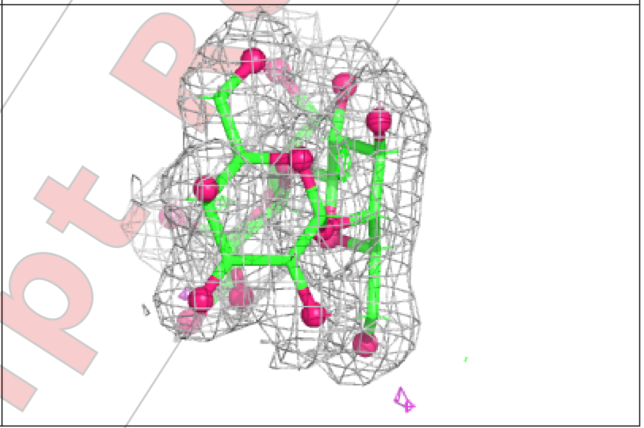

6.4 Ligands (i)

In the following table, the Atoms column lists the number of modelled atoms in the group and the number defined in the chemical component dictionary. The B-factors column lists the minimum, median, 95th percentile and maximum values of B factors of atoms in the group. The column labelled 'Q< 0.9' lists the number of atoms with occupancy less than 0.9.

Mol	Type	Chain	Res	Atoms	RSCC	RSR	B-factors(Å^2)	Q<0.9
3	EDO	B	401	4/4	0.95	0.11	28,30,31,37	1

6.5 Other polymers (i)

There are no such residues in this entry.

For Manuscript Review
